# Molecular Markers Specific for the *Pseudomonadaceae* Genera Provide Novel and Reliable Means for the Identification of Other *Pseudomonas* Strains/spp. Related to These Genera

**DOI:** 10.3390/genes16020183

**Published:** 2025-02-02

**Authors:** Bashudev Rudra, Radhey S. Gupta

**Affiliations:** Department of Biochemistry and Biomedical Sciences, McMaster University, Hamilton, ON L8N 3Z5, Canada; rudrab@mcmaster.ca

**Keywords:** taxon-specific molecular markers, genomic sequences, phylogenetic analysis, Appindels.com server: prediction of taxonomic affiliation, unclassified *Pseudomonas* spp./strains, *Pseudomonadaceae genera*, *Pseudomonas aeruginosa*

## Abstract

**Background/Objectives:** Taxon-specific conserved signature indels (CSIs) exhibit a strong predictive ability of being found in other members of specific taxa/genera. Recently, multiple exclusively shared CSIs were identified for several newly described *Pseudomonadaceae* genera (viz. *Aquipseudomonas*, *Atopomonas*, *Caenipseudomonas*, *Chryseomonas Ectopseudomonas*, *Geopseudomonas*, *Halopseudomonas*, *Metapseudomonas*, *Phytopseudomonas*, *Serpens*, *Stutzerimonas*, *Thiopseudomonas*, and *Zestomonas*). This study examines the potential applications of these CSIs for identifying other *Pseudomonas* spp. (strains) related to these genera. **Methods:** This work utilized the AppIndels.com server, which uses information regarding the presence of known taxon-specific CSIs in a genome for predicting its taxonomic affiliation. For this purpose, sequence information for different CSIs specific for the *Pseudomonadaceae* species/genera were added to the server’s database. **Results:** The AppIndels server was used to predict the taxonomic affiliation of 1972 genomes of unclassified *Pseudomonas* spp. (strains/isolates). Based upon finding a significant number of CSIs matching a specific taxon, the AppIndels server made positive predictions regarding the taxonomic affiliation of 299 examined genomes into the following clades/genera: *Pseudomonas sensu stricto* clade (46), *Pseudomonas aeruginosa* (64), *Ectopseudomonas* (46), *Chryseomonas* (32), *Stutzerimonas* (31), *Metapseudomonas* (22), *Aquipseudomonas* (21), *Phytopseudomonas* (17), *Halopseudomonas* (9), *Geopseudomonas* (4), *Thiopseudomonas* (3), *Serpens* (2), and *Caenipseudomonas* and *Zestomonas* (1 each). Phylogenetic studies confirmed that the taxonomic predictions by the server were 100% accurate. **Conclusions:** Our results demonstrate that the CSIs specific for *Pseudomonadaceae* species/genera, in conjunction with the AppIndels server, provides a novel and useful tool for identifying other species/strains affiliated with these species/genera. Phylogenetic studies suggest that many examined *Pseudomonas* strains constitute novel species in the indicated genera.

## 1. Introduction

The family *Pseudomonadaceae* harbors several genera of which the genus *Pseudomonas* is one of the largest and earliest known prokaryotic genera [1,2]. The genus *Pseudomonas* encompasses >300 species representing more than 2/3rd of the *Pseudomonadaceae* species. Extensive earlier work on *Pseudomonas* species, using phylogenetic trees constructed based on multiple different sets of genes/proteins, including core genomic proteins, has reliably established that the species from this genus do not form a monophyletic lineage. In phylogenetic trees, *Pseudomonas* species generally form three main groupings or lineages, referred to as the Pertucinogena, the Aeruginosa, and the Fluorescens lineages [3,4,5,6,7,8,9,10]. Additionally, species from both the Aeruginosa and Fluorescens lineages form multiple distinct genus-level clades, which are not specifically (i.e., evolutionarily) related to each other [5,9,11]. Species from other genera, including *Azomonas*, *Azotobacter*, *Chryseomonas*, *Entomomonas*, and *Thiopseudomonas*, branch in between these clades/lineages, demonstrating the polyphyletic nature of *Pseudomonas* species [3,4,5,6,7]. It is widely recognized that in accordance with the code governing the nomenclature of Prokaryotes [12], of the observed *Pseudomonas* species clades, only the species from the “Aeruginosa clade”, which contains the type species *P. aeruginosa* of the genus *Pseudomonas*, should be recognized as the genus *Pseudomonas* [4,6,9,11,13,14,15,16].

It is important to note that the nomenclature type of the genus *Pseudomonas*, *P. aeruginosa*, is an important human pathogen capable of causing a wide array of life-threatening acute and chronic diseases [17,18]. However, this genus also includes some animals and plant pathogenic species, as well as other economically and ecologically significant species [19,20,21,22,23]. Additionally, species from this genus also produce several medically and agriculturally important compounds and a multitude of biologically active secondary metabolites [24,25,26]. Thus, it is of much importance to develop a reliable and informative classification scheme for *Pseudomonas* species, where different monophyletic groups of organisms are reliably demarcated and suitably named to distinguish them from each other. Naming different groups of organisms by distinct names indicates (implies) that all species bearing a specific genus name are more closely related to each other, and they commonly share several genotypic, phenotypic, and other properties (e.g., pathogenicity profile or potential), which differentiate them from other genera [15,27,28,29,30]. Distinct genus names also convey useful information about organisms, including their involvement in disease causation (i.e., risk group category), outbreaks, and diagnostic and treatment strategies [29,30]. Thus, taxonomy provides the central framework regarding our current understanding of microorganisms.

With the availability of genome sequences, extensive work has been carried out in the past few years to clarify the evolutionary relationships and classification of *Pseudomonas* species using multiple genome sequence-based approaches. The approaches used include the construction of phylogenetic trees based upon different large datasets of core genomic proteins [4,5,6,7,11,13,16,31] and an assessment of the overall relatedness of species from different clades based on genomic similarity matrices such as average nucleotide identity (ANIb) [4,16], average amino acid identity (AAI) [4,11], and the percentage of conserved proteins (POCP) [4,11,31]. In addition, analyses of genome sequences have also proven instrumental in the identification of highly specific molecular markers, such as conserved signature indels (CSIs) in genes/proteins, which are uniquely shared characteristics of species from different clades and afford unambiguous means for both distinguishing and the demarcation of different specific clades [5,11,32,33,34]. Based upon the consistent evidence acquired using different genomic approaches, most of the *Pseudomonas* species from the Pertucinogena and Aeruginosa lineages have now been reclassified into several novel genera (viz. *Aquipseudomonas*, *Atopomonas*, *Caenipseudomonas*, *Ectopseudomonas*, *Geopseudomonas*, *Halopseudomonas*, *Metapseudomonas*, *Phytopseudomonas*, *Stutzerimonas*, and *Zestomonas*) [4,5,6,11] and some preexisting genera (*Chryseomonas*, *Paraburkholderia*, *Serpens*, *Stenotrophomonas*, *Thiopseudomonas*, and *Xanthomonas*) [35,36]. Importantly, these studies have led to the identification of multiple highly specific molecular markers (i.e., CSIs) that are uniquely shared characteristics of the species noted above. Additionally, several molecular markers have also been identified, which are exclusively found in the species from the genus *Pseudomonas sensu stricto*, *Azotobacter*, and *Azomonas* and for the species *P. aeruginosa*.

Due to the presence of *Pseudomonas*-related species in diverse niches and environments, including soil, water, and plant and animal tissues [10,37], and as its type species, *P. aeruginosa*, is an important human pathogen [17,18], species related to this genus are subjects of extensive studies and novel species and strains related to this genus are continually being discovered at a rapid pace [38]. Since 2022 alone, more than 100 novel species related to *Pseudomonas* are listed in the List of Prokaryotic Names with Standing in Nomenclature (LPSN) server [38]. However, in addition to the species with validly published names, the NCBI server holds genome sequences for >2000 uncharacterized *Pseudomonas* spp. (strains or isolates). Several of these uncharacterized strains/isolates will likely be identified as novel species. However, there is no information available at present regarding their taxonomic affiliation. In our earlier work on *Bacillus* related and other genera we have provided convincing evidence that the CSIs specific for different genera exhibit a high degree of predictive ability to be found in other members of these genera, and the presence of known taxon-specific CSIs in a genome sequence can be used to predict its taxonomic affiliation. The predictive abilities of the CSIs to be found in other related species form the basis of the recently developed AppIndels.com server, which based upon the presence of known taxon-specific CSIs in a submitted genome sequence can predict its taxonomic affiliation [39].

In this study, we have used the AppIndels.com server to determine whether based upon the information for the CSIs specific to different *Pseudomonadaceae* genera it can predict the phylogenetic/taxonomic affiliations of several of the unclassified *Pseudomonas* spp. (strains). The results of these studies presented here show that based upon the information for identified *Pseudomonadaceae* CSIs, the server was able to predict the taxonomic affiliation of 299 unclassified *Pseudomonas* strains/isolates into 14 *Pseudomonadaceae* clades/genera. Phylogenetic studies conducted on these strains show that the predictions made by the server regarding the taxonomic affiliations of these 299 strains were 100% accurate. Thus, the identified CSIs specific for the *Pseudomonadaceae* genera provide a novel and useful means for the identification of other novel or unclassified *Pseudomonas* species/strains related to these genera.

## 2. Materials and Methods

### Analysis of Pseudomonas spp. Using the AppIndels Server

Sequence information for the CSIs specific to different *Pseudomonadaceae* clades/genera was added to the database of the AppIndels server (https://appindels.com/, accessed on 11 March 2024) [39]. Genome sequences for 2000 unclassified strains/isolates of *Pseudomonas* spp. were downloaded (in .faa format) from the NCBI Genome Database (https://www.ncbi.nlm.nih.gov/datasets/genome/, accessed on 1 June 2024) [39]) [40]. Details of these downloaded genomes, including their strain numbers, accession numbers, GC content, and genome sizes, are given in Supplementary Tables S1–S3. Of these genomes, some genomes that contained either <100 Kb sequence information or were indicated as contaminated were excluded from analyses. The remaining 1972 genomes were analyzed using the AppIndels server one at a time as indicated in earlier work and on the server’s main page. The predictions made by the server regarding the taxonomic affiliation of the submitted sequence and the number of CSIs identified in it specific for the predicted genus were recorded.

A maximum-likelihood phylogenetic tree for the *Pseudomonas* spp. strains for which taxonomic assignments were made by the server, along with sequences of representative species from different examined *Pseudomonadaceae* genera, was constructed based on the concatenated sequences for 118 conserved proteins comprising the phyloeco set for the class Gammaproteobacteria [41]. The tree was constructed using an internally developed pipeline, as described in our recent work [5,34]. The tree was labeled and formatted using MEGA X [42].

## 3. Results

### 3.1. Predictive Ability of a CSI Specific for the Genus Halopseudomonas

**Figure 1:**
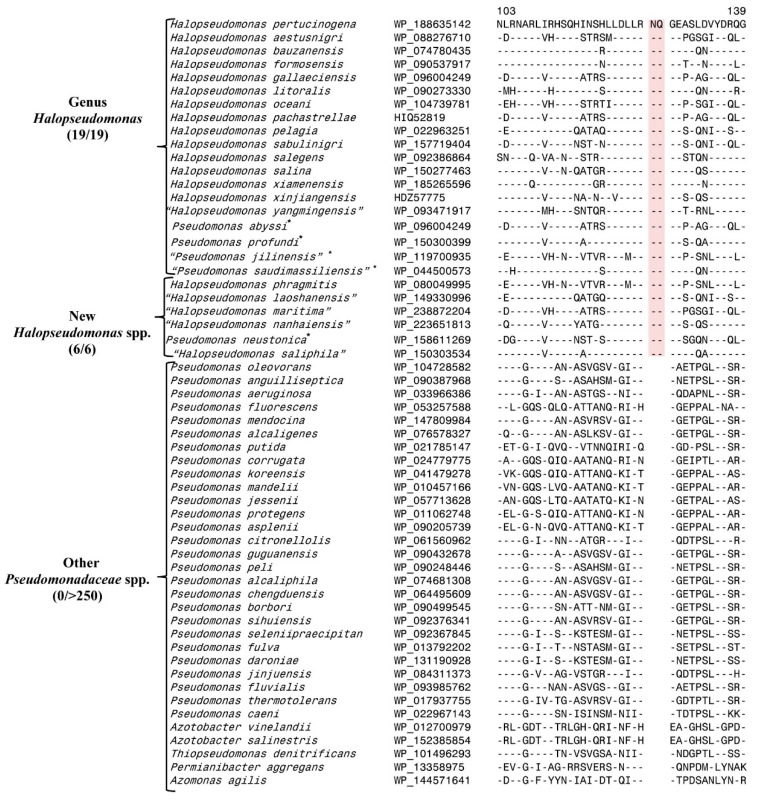
Partial sequence alignment showing a two amino acid insertion (CSI) in the flagellar FlgN protein (highlighted in pink color) described in our earlier work [5], which is specific for the genus *Halopseudomonas*. Sequences for six new *Halopseudomonas*-related species have since become available, and all of them share this CSI, demonstrating the predictive ability of this CSI. The species marked with the symbol * have not yet been reclassified as *Halopseudomonas* due to the lack of availability of type strains in two different culture collections, or some species are listed in the LPSN under the genus *Neopseudomonas*, which is a synonym of *Halopseudomonas* [38]. Quotation marks “ ” surrounding a species name indicates that this name is not yet validly published. The dashes (-) in the alignment indicate identity with the amino acids on the top line. Accession numbers for different sequences are indicated in the second column, and the numbers at the top indicate the position of this sequence fragment within the protein sequences.

Earlier work on CSIs specific for multiple prokaryotic taxa provides compelling evidence that these molecular characteristics exhibit a high degree of predictive ability to be found in other species related to a specific taxon. To illustrate, in Figure 1, we show the results for a CSI specific for the genus *Halopseudomonas* [5]. This genus was created in 2021 by the reclassification of *Pseudomonas* species, which corresponded to the Pertucinogena lineage. More than 20 CSIs specific to the genus *Halopseudomonas* were identified in this earlier study, and the example depicted in Figure 1 shows the results for one of these CSIs, where a 2 aa insert in a conserved region of the flagellar protein FlgN was present exclusively in all 19 *Pseudomonas* species that corresponded to the genus *Halopseudomonas*.

Two of these species in this figure are listed as “*Pseudomonas*” as they have not yet been reclassified as *Halopseudomonas* due to the lack of availability of the type strains in two different culture collections. Since the publication of this work, six other species related to *Halopseudomonas* have been described [4,6,43,44]. Some of these species presently are either not validly published (indicated by their placement within “ ”) or they are misclassified into the genus *Neopseudomonas* [6], which is a homotypic synonym of *Halopseudomonas* [38]. Nonetheless, as shown in Figure 1, the 2 aa CSI specific for the *Halopseudomonas* is commonly and uniquely shared by all six newly described species related to *Halopseudomonas*, but it is not found in any other *Pseudomonadaceae* species. In a phylogenetic tree that we have constructed, all species share this CSI group reliably within a clade corresponding to the genus *Halopseudomonas* (Figure S1). These results provide further evidence supporting the predictive ability of taxon-specific CSIs to be found in other species/strains that are related to them.

### 3.2. Examining the Usefulness of the CSIs Specific for the Pseudomonadaceae Genera for Determining the Taxonomic Affiliation of Unclassified Pseudomonas spp. Using the AppIndels.com Server

As indicated earlier, in addition to the genomes for >300 *Pseudomonas* species with validly published names, the NCBI database also holds genome sequences for >2000 unclassified strains/isolates of *Pseudomonas* spp. An earlier study by Hess et al. [7] provides evidence that these unclassified strains encompass enormous genetic diversity, which remains to be understood. Thus, it is important to develop novel means or tools by which the genetic diversity and taxonomic affiliation of these unclassified strains could be assessed. In this work, we have investigated whether the identified CSIs specific to several *Pseudomonadaceae* genera can be used for identifying unclassified *Pseudomonas* spp./strains that are related to these genera. These analyses were carried out using the AppIndels.com server, which has been specifically created to take advantage of the predictive abilities of the known taxon-specific CSIs, to identify other species/strains related to them. The working of the AppIndels.com server has been described in detail elsewhere [39], but it is briefly explained below.

The AppIndels.com server is a web-based tool that uses sequence information for validated CSIs specific for known prokaryotic taxa for determining the presence of these molecular characteristics in any input genome sequence [39]. Based on the taxon-specificities of the CSIs in the server’s database and their predictive ability to be found in other members of these taxa, if the server identifies that the input genome sequence contains significant numbers of CSIs matching a specific taxon, it predicts that the analyzed genome (strain/species) is affiliated with that taxon. The AppIndels server database presently contains sequence information for >1000 previously identified CSIs specific to different (>100) prokaryotic genera [39]. We have now added to this database the sequence information for different identified CSIs specific to the *Pseudomonadaceae* genera [5,11]. In Table 1, we have provided information regarding the *Pseudomonadaceae* genera/taxa for which the CSIs have been identified and the numbers of CSIs, which are specific for each of these genera or taxa. This list also includes several CSIs that are specific for the species *P. aeruginosa* and *P. paraeruginosa* [45].

The last column in Table 1 indicates the weight values given to individual CSIs from different taxa. The rationale of giving weight values to different CSIs is discussed in detail in earlier work [39]. However, its main purpose is to increase the specificity of taxon prediction by the AppIndels server by requiring that multiple CSIs specific to a given taxon be present before a positive identification is made. When a genome sequence is uploaded or submitted to the AppIndels.com server, it conducts BLASTp searches on the submitted genome against the sequences of all CSIs in its database. Based on these searches, the server identifies matching sequences in the submitted genome where the indels of specific lengths are present in protein sequences in the exact location as present in the protein sequences in the CSI database. The server then gathers information regarding the taxon specificities of different matching CSIs. If the combined weight of all CSIs matching a specific taxon exceeds the threshold value of 1.0, the server makes a positive prediction that the submitted genome is affiliated with the indicated taxon. As all CSIs specific for the *Pseudomonadaceae* genera/clades have a weight value of 0.4 or less (Table 1), the server will make a positive identification for any *Pseudomonadaceae* genus/clade only when three or more CSIs matching that taxon are found in the submitted genome. As all described CSIs for the *Pseudomonadaceae* species/genera exhibit a high degree of specificity for the indicated taxon (barring an isolated exception) [5,11,45], the possibility of finding three CSIs matching a specific genus/taxon in the genome of an unrelated species/strain is considered highly unlikely.

To test the usefulness of identified CSIs using the AppIndels server, genome sequences were downloaded for 2000 stains/isolates of *Pseudomonas* spp. from the NCBI genome database. Of these, 28 genomes, where the genome sequence consisted of <100 Kb, or was indicated as contaminated, were not further analyzed. Of the remaining 1972 genomes, 266 genomes were chromosomes or complete (Table S1), 1197 consisted of contigs (Table S2), and 509 were scaffolds (Table S3). Some information regarding these genomes, including their strain numbers, accession numbers, assembly stage, G-C content (mol%), and genome sizes, is provided in the Supplementary Material (Tables S1–S3). The analyses on these genomes were conducted using the AppIndels server by uploading the sequences of these genomes, one at a time, onto the server. The server checks the uploaded genome sequence for the presence of CSIs matching different taxa in its database. If the server identifies significant numbers of CSIs matching any specific taxon, then the result from the server shows a positive match to that taxon. In such cases, the server also provides information regarding the number of CSIs matching the predicted taxon. However, if the submitted genome corresponds to a taxon/genus for which no CSIs are present in the server or if the total weight of the identified CSIs is less than the threshold value of 1.0, then the server shows a negative “None” result.

**Figure 2:**
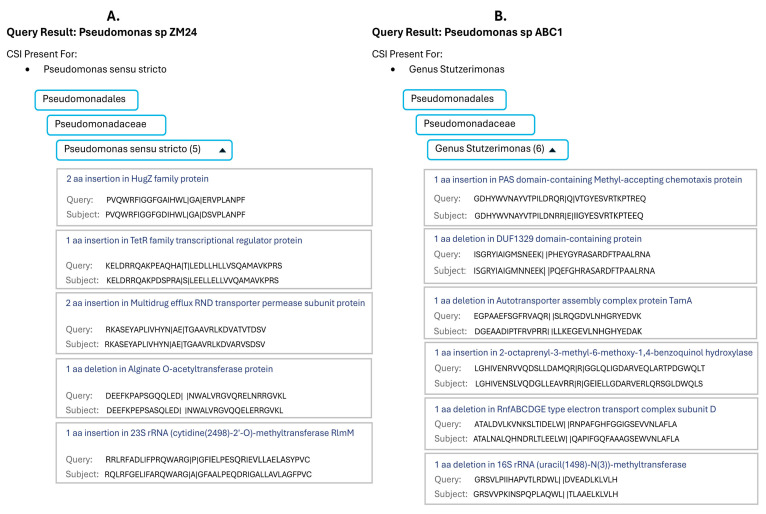
The results from the AppIndels server for the genome sequences of two representative unclassified *Pseudomonas* spp./strains. (**A**) The genome of *Pseudomonas* strain ZM24 is predicted by the server as affiliated with the *Pseudomonas sensu stricto* clade, and it contained five CSIs specific for this clade. (**B**) The *Pseudomonas* strain ABC1 was identified by the server as belonging to the genus *Stutzerimonas*, and it contained six CSIs specific to this genus.

In Figure 2, we show the results obtained from the server for two *Pseudomonas* strains/isolates. The server indicates that the strain ZM24 is related to the *Pseudomonas sensu stricto* clade, and its genome contained five CSIs specific for this clade (Figure 2A). On the other hand, the server predicted that the genome of strain ABC1 is related to the genus *Stutzerimonas*, and its genome contained six CSIs specific for this genus (Figure 2B). In addition to indicating the numbers of CSIs specific to the predicted taxon, the server also provides sequence information for all matching CSIs, which can be viewed upon clicking the down arrow beside the number of CSIs.

Based on the analysis of genome sequences for 1972 examined *Pseudomonas* strains/isolates, the server made specific predictions regarding the taxonomic affiliations of 299 of the examined genomes to specific *Pseudomonadaceae* genera. The results from the server for the genomes of all 299 *Pseudomonas* strains/isolates for which specific predictions were made are shown in Table S4, and a summary of these results is presented in Table 2. In Table 2, we have organized the results from the server for different strains according to their predicted affiliation for the *Pseudomonadaceae* genera. Table 2 also shows the numbers of CSIs (range) specific for the indicated genus/species, which were identified in the analyzed genomes.

As seen from Table 2, in all cases, the predicted affiliation of any genome to a specific *Pseudomonadaceae* species/genera is based on the shared presence of a minimum of three CSIs specific to that taxon. The numbers of CSIs identified for different genera (or species) in the analyzed genomes varied from a low of 3 for the genus *Serpens* to more than 20 for *Halopseudomonas*. This variation is solely due to the differences in the number of CSIs that have been identified for different genera (see Table 1) [5,11,45]. The results presented in Table 2 show that of the genomes for which the server made specific predictions, about 20% corresponded to the species *P. aeruginosa*. Other *Pseudomonadaceae* genera to which large numbers of analyzed strains (genomes) belonged included the *Pseudomonas sensu stricto* clade (46 strains), *Ectopseudomonas* (46 strains), *Chryseomonas* (32 strains), *Stutzerimonas* (31 strains), *Metapseudomonas* (22 strains), *Aquipseudomonas* (21 strains), *Phytopseudomonas* (17 strains), *Halopseudomonas* (9 strains), and *Geopseudomonas* (4 strains). The server also predicted that a limited number of strains are affiliated with the genera *Caenipseudomonas*, *Serpens*, *Thiopseudomonas*, and *Zestomonas*, which consist of only a few species [5,11].

**Figure 3:**
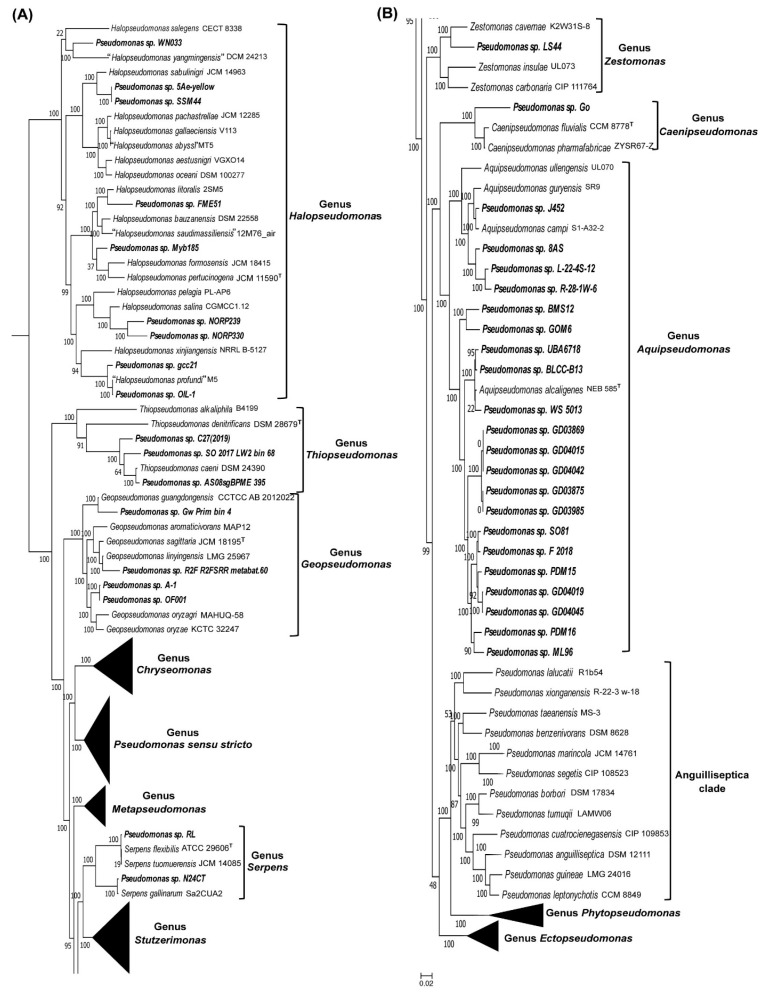
A phylogenetic tree based on genome sequences for the representative species, including type species of different *Pseudomonadaceae* genera and genomes of different *Pseudomonas* spp. (strains/isolates) for which positive predictions were made by the server regarding affiliation with specific clades/genera (Table 2 and Table S4). For the ease of visualization of information for different strains, the clades for some genera, viz. *Chryseomonas*, *Ectopseudomonas*, *Metapseudomonas*, *Phytopseudomonas*, *Pseudomonas sensu stricto*, and *Stutzerimonas*, are compressed in this figure. The figure is shown in two parts (**A**,**B**), and part B is a continuation of (**A**). All *Pseudomonas* strains for which the server made taxonomic predictions branched with 100% accuracy with the indicated genera in these trees.

**Figure 4:**
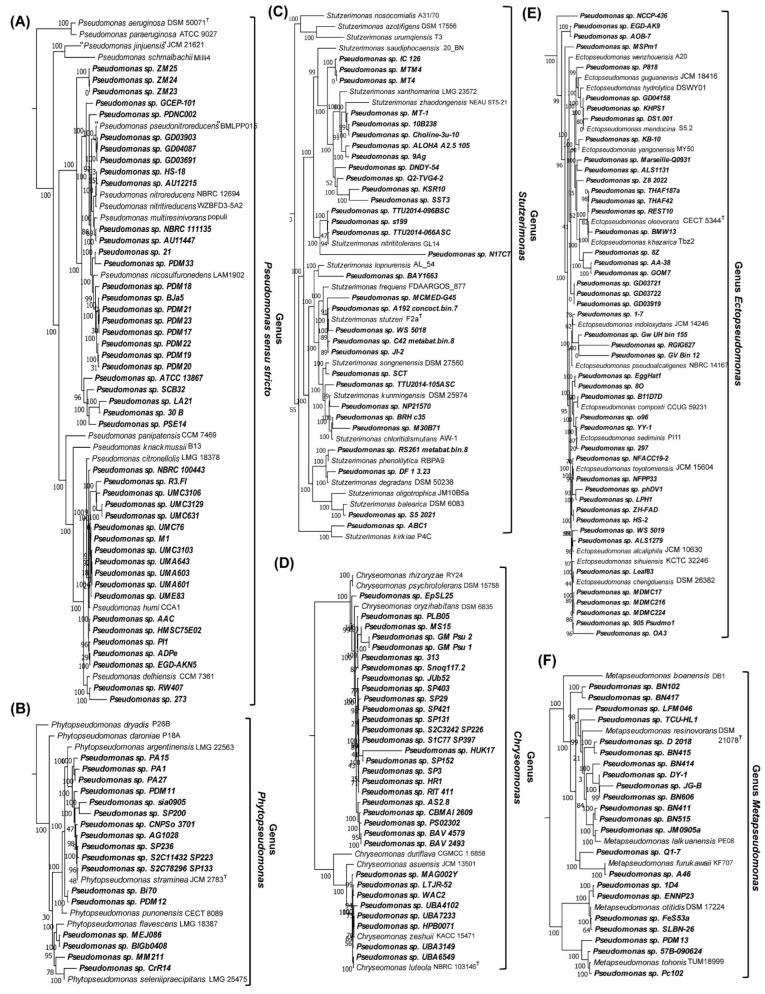
Phylogenetic branching of *Pseudomonas* spp. (strains/isolates), which based upon the results obtained from AppIndels server (Table 2 and Table S4) were predicted to be related to the genera (**A**) *Pseudomonas sensu stricto*, (**B**) *Phytopseudomonas*, (**C**) *Stutzerimonas*, (**D**) *Chryseomonas*, (**E**) *Ectopseudomonas*, and (**F**) *Metapseudomonas*. All strains for which the server made taxonomic predictions branched with 100% accuracy with the indicated genera in this tree.

We have examined the reliability of taxon predictions by the server by constructing a phylogenomic tree based on genome sequences of different *Pseudomonas* strains for which the server made taxonomic predictions. This tree was constructed based on concatenated sequences of 118 conserved proteins (corresponding to the phyloeco set for the class *Gammaproteobacteria*), and it also included the sequences of representative species from relevant *Pseudomonadaceae* genera. We show the results from this tree in Figure 3. Due to the considerable number of strains in this tree, we have compressed the clades for some *Pseudomonadaceae* genera in Figure 3. However, the uncompressed results for these clades are presented in Figure 4. In the phylogenetic trees shown in Figure 3 and Figure 4, all *Pseudomonas* strains/isolates for which the server made taxonomic predictions grouped reliably (100% concordance) with the other species from the indicated genera (Figure 3, Figure 4 and Figure S2). Based upon the branching of different *Pseudomonas* strains/isolates in Figure 3 and Figure 4, while many unclassified strains are closely related to the known species, several other strains branched distinctly from the known species. Thus, many of these strains may constitute novel species within the indicated genera.

## 4. Discussion

Members of the genus *Pseudomonas*, which are genetically and evolutionarily highly diverse, are widely distributed in different environments. This group includes species that are opportunistic pathogens of humans, animals, and plants and other species of economic and ecological significance [17,18,19,46,47]. For example, the type species of this genus, *P. aeruginosa*, which is one of the most researched species, is an opportunistic multidrug-resistant human pathogen capable of infecting multiple tissues, especially in individuals with weakened immune systems, and is often responsible for serious illnesses, such as ventilator-associated pneumonia and several sepsis syndromes [48,49,50]. *P. aeruginosa* infections in patients with cystic fibrosis cause significant economic burden in the health care industry [18]. Due to its resistance to different antibiotics, the World Health Organization recognizes *P. aeruginosa* as one of the six important pathogens posing greatest threats to humans in terms of antibiotic resistance [50,51]. On the other hand, some *Pseudomonas* species, such as *P. syringae*, are pathogenic to plants [19], whereas other species, such as *P. fluorescens*, are beneficial to plants and have been used in the agriculture industry for sustainable plant growth as well as disease management [24]. Several other *Pseudomonas* species have found significant roles as biocontrol agents, as bioremediation agents, as detectors of food spoilage agents in milk [25], and in the degradation of anthropogenic pollutants [26].

In view of the importance of these species from clinical and other perspectives, this group of species is extensively studied, and it constitutes one of the fastest growing groups of bacteria [4]. In recent years, extensive work using genomic approaches has been carried out to more reliably delineate the evolutionary relationships and classification scheme for *Pseudomonas* and related species. These studies have led to the reclassification of >150 *Pseudomonas* species into 14 novel genera [5,11]. Members of all these newly described genera can be reliably distinguished from each other based upon multiple highly specific molecular markers (i.e., CSIs) that are uniquely shared characteristics of the species from these genera. Similarly, the clade corresponding to the genus *Pseudomonas sensu stricto*, which harbors *P. aeruginosa*, can also be reliably distinguished from all other *Pseudomonas* based on multiple exclusively shared CSIs [11]. However, the genetic diversity of *Pseudomonas* extends far beyond the known species (>300) with validly published names. The NCBI [40] harbors genomes for >2000 uncharacterized strains/isolates of *Pseudomonas* species, for which no information is available regarding their phylogenetic affiliation. As these uncharacterized strains are likely to harbor many novel species related to both the known *Pseudomonadaceae* genera as well as other novel taxa related to these bacteria [6], it is important to characterize them. However, there is no easy-to-use methods available for dependably identifying strains that are related to the existing *Pseudomonadaceae* genera.

Therefore, the objective of this study was to determine whether the CSIs specific for different *Pseudomonadaceae* genera, due to their known predictive ability to be found in other group members, can be used to identify other unclassified *Pseudomonas* (spp.) strains that are related to these genera. These investigations were greatly facilitated by the recent development of AppIndels.com server, which based upon the presence of known taxon-specific CSIs in a genome sequence, can predict its taxonomic affiliation [39]. In this work, the AppIndels server was used after supplementing its database with the sequence information for different CSIs specific to the *Pseudomonadaceae* genera for predicting the taxonomic affiliations of genome sequences for 1972 unclassified *Pseudomonas* strains/isolates. The results presented here show that based upon the identified CSIs for the *Pseudomonadaceae* genera, the server was able to predict the taxonomic affiliation of 299 of these unclassified *Pseudomonas* strains into 14 distinct clades of *Pseudomonadaceae* species/genera. The genera or species groups into which these unclassified *Pseudomonas* strains/isolates were assigned included the *Pseudomonas sensu stricto* clade (46 strains), *Ectopseudomonas* (46 strains), *Chryseomonas* (32 strains), *Stutzerimonas* (31 strains), *Metapseudomonas* (22 strains), *Aquipseudomonas* (21 strains), *Phytopseudomonas* (17 strains), *Halopseudomonas* (9 strains), *Geopseudomonas* (4 strains), *Thiopseudomonas* (3 strains), *Serpens* (2 strains), *Caenipseudomonas* (1 strain), and *Zestomonas* (1 strain). In addition, 64 *Pseudomonas* strains/isolates were identified as *P. aeruginosa*. In all cases, the assignment of *Pseudomonas* strains to different *Pseudomonadaceae* genera (or to *P. aeruginosa*) was based on the shared presence of multiple (minimum 3) CSIs, which are exclusive characteristics of the indicated genera. The results of phylogenetic studies conducted here confirm that the taxonomic predictions made by the server were 100% in agreement with the branching of these strains with the species from the indicated genera. These results provide further strong evidence regarding (i) the predictive abilities of the taxon-specific CSIs to be found in other (unclassified) members of these taxa and (ii) the conclusion that the use of these molecular markers provides a novel and trustworthy means for the identification of other species/strains related to these genera [39].

Although the AppIndels server accurately predicted the taxonomic affiliations of 299 *Pseudomonas* strains, it provided no results for the remainder of the strains. This is not surprising because the AppIndels server can make taxonomic predictions for only those strains that are related to the taxa for which CSIs are known and present in the server’s database [39]. As noted previously, the genus *Pseudomonas* is a very large and diverse grouping of microorganisms, harboring >300 validly named species that form multiple distinct clades/lineages [4,6,7,8,9,11,16]. Thus far, CSIs have been identified for only a limited number of these groupings, consisting mainly of the genus *Halopseudomonas* and some clades/genera within the Aeruginosa lineage. However, a vast majority of the *Pseudomonas* species, representing more than two-thirds of named species, are part of the Fluorescens lineage, which is composed of multiple distinct genus-level clades and subclades [7,9,13,16,31,37]. No CSIs are known at present for the species from different clades and subclades of the Fluorescens lineage. In addition, no CSIs have been identified for the Anguilliseptica clade of species and many other species within the Aeruginosa lineage (viz. *P. benzenivorans*, *P. cuatrocienegasensis*, *P. indica*, *P. kuykendallii*, *P. lalucatii*, *P. mangiferae*, *P. mangrovi*, *P. matsuisoli*, and *P. pohangensis*), which branch distinctly from the described clades/genera. In view of the paucity of CSIs for these other groups/clades of *Pseudomonas* species, if an examined strain (genome) is affiliated with these species clades/genera, the server will not be able to make any taxonomic predictions for those strains. Therefore, as indicated on the server’s website, while the absence of any taxonomic prediction by the server is not very informative, a specific prediction by the server regarding taxonomic affiliation is a highly trustworthy result.

It should be noted that the genomes of *Pseudomonas* spp./strains for which the server was able to make correct taxonomic predictions consisted of different assembly stages ranging from chromosome and complete to contigs and scaffolds (see Tables S1–S3). Previously, we have also shown that based on genome sequence information, the server can also predict the taxonomic affiliation of uncultured strains/isolates [52]. These results and observations indicate that the AppIndels server provides a valuable and easy-to-use tool for the identification and taxonomic characterization of cultured and uncultured strains/isolates for the species/genera for which CSIs are known. Based upon the phylogenetic branching of *Pseudomonas* spp./strains for which taxonomic predictions were made by the server, several of these strains branched distinctly from the other known species within these genera (Figure 3 and Figure 4). Thus, upon further characterization, a number of these strains would likely constitute novel species within these genera. This should lead to a considerable increase in the genetic diversity of species within these genera advancing our understanding of the *Pseudomonas*-related species/genera.

It should be noted that the AppIndels server, in addition to its demonstrated utility for predicting the taxonomic affiliation of any genome-sequenced strains/isolate, also provides a novel and useful diagnostic tool. Amongst the *Pseudomonas* species, *P. aeruginosa* is of particular significance, as it can cause numerous life-threatening diseases in humans [17,18]. Hence, an accurate identification of this species from other closely related species is of considerable importance in clinical settings. The results presented here show that based upon the identified CSIs, the server can reliably distinguish *P. aeruginosa* from all other *Pseudomonas*-related species, including other species from the *Pseudomonas sensu stricto* clade. Of particular importance is the fact that the server can also reliably distinguish *P. aeruginosa* from *P. paraeruginosa*. The latter species was recently created from *P. aeruginosa* by the transfer of several strains, which lacked the Type III secretion system (i.e., differing in terms of pathogenicity) and produced various biosurfactants [53], into this new species [45]. However, *P. paraeruginosa* is genetically closely related to *P. aeruginosa*, and it is difficult to distinguish between these two species with the most available diagnostic methods [45]. However, the AppIndels server provides a rapid and easy-to-use method to reliably detect the presence of *P. aeruginosa* based on genome sequence information. Additionally, based on genome sequence information, the server can also rapidly and reliably detect the presence of any species/strains related to the *Pseudomonas sensu stricto* clade, which due to being part of this monophyletic clade may share its pathogenicity traits for humans [29]. Similarly, based upon the CSIs specific for other species/genera, the server can also reliably detect the presence of other related species based on genome sequence information.

Lastly, based upon earlier work on CSIs in genes/proteins sequences, these molecular characteristics, in addition to their specificity and predictive abilities for reliable identification of species from different clades, also play important/essential functions in the group of organisms for which they are specific [11,33,54,55,56,57]. Hence, genetic, biochemical, and functional studies on the CSIs specific for different genera provide means for the identification of novel biochemical and other characteristics that are specific to these organisms.

## Figures and Tables

**Table 1:** List of *Pseudomonadaceae* Genera for which CSIs have been identified.

Genera/Species Name	No. of Identified CSIs	Weight Value of Each CSI
*Aquipseudomonas*	6	0.4
*Atopomonas*	22	0.2
*Azomonas*	5	0.4
*Azotobacter*	10	0.4
*Caenipseudomonas*	8	0.4
*Chryseomonas*	11	0.3
*Ectopseudomonas*	5	0.4
*Geopseudomonas*	15	0.3
*Halopseudomonas*	24	0.2
*Metapseudomonas*	5	0.4
*Phytopseudomonas*	12	0.3
*Pseudomonas sensu stricto*	6	0.4
*Serpens*	3	0.5
*Stutzerimonas*	7	0.4
*Thiopseudomonas*	6	0.3
*Zestomonas*	5	0.4
*P. aeruginosa*	7	0.3
*P. paraeruginosa*	5	0.4

**Table 2:** The results from the AppIndels Server regarding the taxonomic affiliations of the genome sequences of 299 unclassified *Pseudomonas* spp.

Genera/Species	No. of Strains	Range of CSIs	*Pseudomonas* spp. Strain Nos.
*Pseudomonas sensu stricto*	46	5–6	21, 273, 30_B, AAC, ADPe, ATCC 13867, AU11447, AU12215, BJa5, EGD-AKN5, GCEP-101, GD03691, GD03903, GD04087, HMSC75E02, HS-18, LA21, M1, NBRC 111135, NBRC100443, PDM17, PDM18, PDM19, PDM20, PDM21, PDM22, PDM23, PDM33, PDNC002, PI1, PSE14, R3.Fl, RW407, SCB32, UMA601, UMA603, UMA643, UMC3103, UMC3106, UMC3129, UMC631, UMC76, UME83, ZM23, ZM24, ZM25.
*P. aeruginosa*	64	5–7	203-8, 17023526, 17023671, 17033095, 17053182, 17053418, 17053703, 17063399, 17072548, 17073326, 17102422, 17103552, 17104299, 18073667, 18082547, 18081308, 18082551, 18082574, 18083194, 18083202, 18083259, 18083286, 18084127, 18092229, 18093371, 18101001-2, 18102011, 18103014, 18113298, 19062259, 19064969, 19072337-2, 19082381, 2VD, 3PA37B6, AF1, AFW1, AK6U, B111, BDPW, BIS, BIS1, CP-1, FDAARGOS_761, HMSC057H01, HMSC072F09, HMSC16B01, HMSC076A11, HMSC060F12, HMSC065H01, HMSC066A08, HMSC065H02, HMSC067G02, HMSC063H08, HMSC058C05, P179, P20, P22, PAH14, Pseudomonas_assembly, PS1(2021), RGIG3665, S33, S68.
*Aquipseudomonas*	21	4–6	8AS, BLCC-B13, BMS12, F(2018), GD03869, GD03875, GD03985, GD04015, GD04019, GD04042, GD04045, GOM6, J452, L-22-4S-12, ML96, PDM15, PDM16, R-28-1W-6, UBA6718, SO81,WS 5013.
*Caenipseudomonas*	1	7	Go_SlPrim_bin_81
*Chryseomonas*	32	6–11	313, AS2.8, BAV 2493, BAV 4579, GM_Psu_1, GM_Psu_2, HUK17, LTJR-52, MAG002Y, PS02302, RIT 411, S1C77_SP397, S2C3242, SP152, SP29, SP3, SP403, SP421, WAC2, HPB0071, Snoq117.2, MS15, JUb52, EpSL25, PLB05, HR1, CBMAI 2609,UBA6549, UBA7233, UBA3149, UBA4102.
*Ectopseudomonas*	46	3–5	297, 07-Jan, 905_Psudmo1, AA-38, ALS1131, ALS1279, AOB-7, B11D7D, BMW13, DS1.001, EGD-AK9, EggHat1, GD03721, GD03722, GD03919, GD04158, GOM7, GV_Bin_12, Gw_UH_bin_155, HS-2, KB-10, KHPS1, LPH1, Leaf83, MDMC17, MDMC216, MDMC224, MSPm1, Marseille-Q0931, NCCP-436, NFACC19-2, NFPP33, o96, OA3, P818, 8O, 8Z, REST10, RGIG627, THAF187a, THAF42, WS 5019, YY-1, Z8(2022), ZH-FAD, phDV1.
*Geopseudomonas*	4	4–15	A-1, OF001, R2F_R2FSRR_metabat.60, Gw_Prim_bin_4.
*Halopseudomonas*	9	20–24	5Ae-yellow, FME51, MYb185, NORP239, NORP330, OIL-1, SSM44, WN033, gcc21.
*Metapseudomonas*	22	3–5	57B-090624, 1D4, A46, BN102, BN411, BN414, BN415, BN417, BN515, BN606, D(2018), DY-1, ENNP23, FeS53a, JG-B, JM0905a, LFM046, PDM13, Pc102, Q1-7, SLBN-26, TCU-HL1.
*Phytopseudomonas*	17	9–12	AG1028, Bi70, BIGb0408, CrR14, CNPSo 3701, MEJ086, MM211, PDM11, PDM12, S2C11432_SP223, S2C78296_SP133, sia0905, SP200_1_metabat2_ genome_mining.44, SP236_1_metabat2_genome_mining.8, PA1, PA15, PA27.
*Serpens*	2	3	N24CT, RL.
*Stutzerimonas*	31	4–7	10B238, 9Ag, A192_concoct.bin.7, ABC1, ALOHA_A2.5_105, BAY1663, BRH_c35, C42_metabat.bin.8, Choline-3u-10, DF_1_3.23, DNDY-54, IC_126, JI-2, KSR10, M30B71, MCMED-G45, MT-1, MT4, MTM4, N17CT, NP21570, Q2-TVG4-2, RS261_metabat.bin.8, S5(2021), SCT, SST3, TTU2014-066ASC, TTU2014-096BSC, TTU2014-105ASC, WS 5018, s199.
*Thiopseudomonas*	3	4–5	AS08sgBPME_395, C27(2019), SO_2017_LW2 bin 68.
*Zestomonas*	1	3	LS44

## Data Availability

Genome sequences for different *Pseudomonas* spp./strains, whose accession numbers are given in Supplementary Tables S1–S3, were downloaded from the NCBI Genome Database (https://www.ncbi.nlm.nih.gov/datasets/genome/, accessed on 30 January 2025).

## Supplementary Materials

The following supporting information can be downloaded at https://www.mdpi.com/article/10.3390/genes16020183/s1, Table S1. List of 266 downloaded *Pseudomonas* spp. genomes (chromosome and complete) for analysis in this study. Table S2. List of 1197 downloaded *Pseudomonas* spp. genomes (contigs) to analyze in this study. Table S3. List of 510 downloaded *Pseudomonas* spp. genomes (scaffold) to analyze in this study. Table S4. Information on the genome sequences of 299 uncharacterized *Pseudomonas* spp. whose taxonomic affiliations were predicted by the AppIndels web server. Figure S1. A maximum-likelihood tree, constructed using concatenated sequences of 118 conserved proteins, depicts the branching of all newly identified species or species with new name combinations that share the CSIs specific to the genus *Halopseudomonas*. Newly described species are highlighted in bold, and non-validly published species are shown within “ ”. Figure S2. A maximum-likelihood tree based on concatenated sequences of 118 conserved proteins showing all strains from the Aeruginosa clade (Genus *Pseudomonas sensu stricto*).
