# Supplementary material for: Molecular Markers Specific for the Pseudomonadaceae Genera Provide Novel and Reliable Means for the Identification of Other Pseudomonas Strains/spp. Related to These Genera
Source: Genes (Basel). 2025 Feb 2;16(2):183. doi: 10.3390/genes16020183 (PMC11855360; doi:10.3390/genes16020183)
Supplement: Supplementary file 1 [file genes-16-00183-s001.zip › genes-3429855-supplementary.pdf]

Supplemental Data for the Article **"Molecular Markers Specific for the *Pseudomonadaceae* Genera Provide Novel and Reliable Means for the Identification of Other *Pseudomonas* strains/spp. Related to These Genera"**

Bashudev Rudra and Radhey S. Gupta*

Department of Biochemistry and Biomedical Sciences,

McMaster University, Hamilton, Ontario CA L8N 3Z5

Table of Contents		
**Figure/Table No.**	**Title of the Figures and Tables**	**Page no.**
**Figure-S1**	A maximum-likelihood tree, constructed using concatenated sequences of 118 conserved proteins, depicts the branching of all newly identified species/or species with new name combinations that share the CSIs specific to the genus *Halopseudomonas*. Newly described species are highlighted in bold and non-validly published species are shown within " ".	**3**
**Figure-S2**	A Maximum-likelihood tree based on concatenated sequences of 118 conserved proteins showing all strains from Aeruginosa clade (Genus *Pseudomonas sensu stricto*)	**4**
**List of Supplementary Tables**		
**Table S1**	List of downloaded 266 *Pseudomonas* spp. genomes (Chromosome and Complete) for analysis in this study	**5**
**Table S2**	List of downloaded 1197 *Pseudomonas* spp. genomes (Contigs) to analyze in this study	**11**
**Table S3**	List of downloaded 509 *Pseudomonas* spp. genomes (Scaffold) to analyze in this study	**38**
**Table S4**	Information on Genome sequences of 299 uncharacterized *Pseudomonas* spp. whose taxonomic affiliations were predicted by the AppIndels web server	**49**

Figure S1: A maximum-likelihood tree, constructed using concatenated sequences of 118 conserved proteins, depicts the branching of all newly identified species/or species with new name combinations that share the CSIs specific to the genus *Halopseudomonas*. Newly described species are highlighted in bold and non-validly published species are shown within " ".

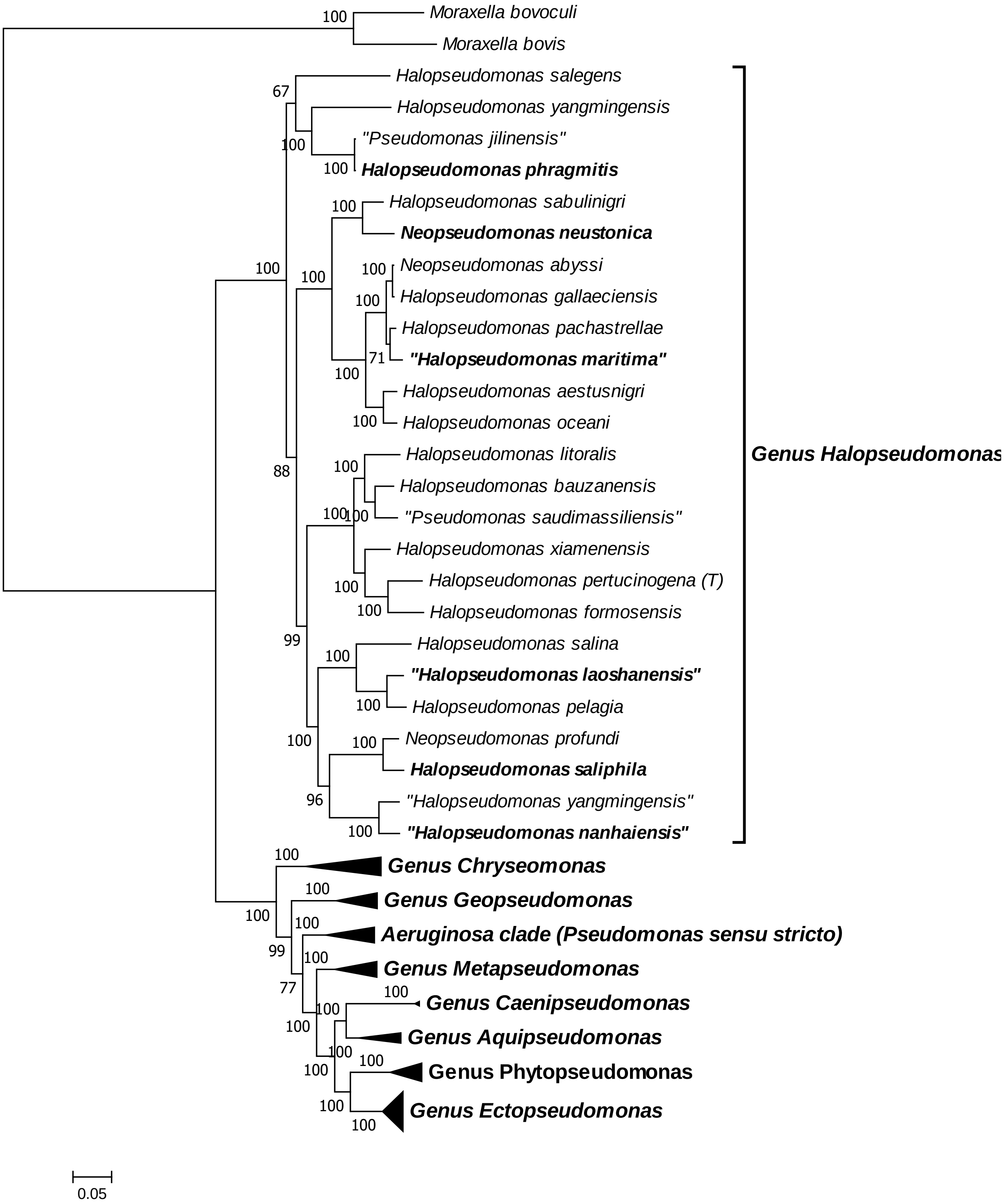

Figure-S2: A Maximum-likelihood tree based on concatenated sequences of 118 conserved proteins. All clades have been compressed except the genus *Pseudomonas sensu stricto*. All 46 strains from the genus *Pseudomonas sensu stricto* and 64 strains of *P. aeruginosa* species are shown in the tree.

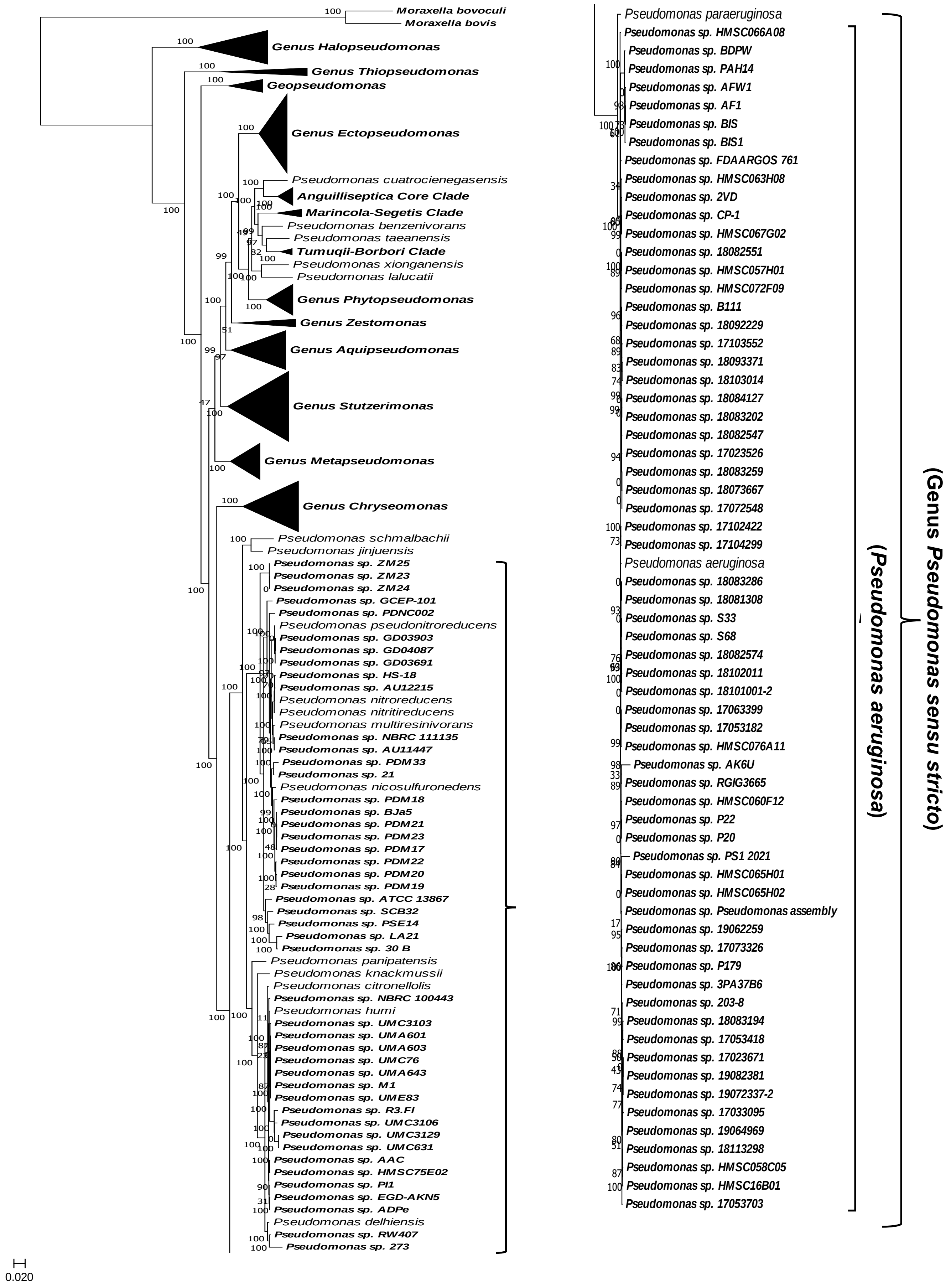

Table S1:
List of downloaded 266 *Pseudomonas* spp. genomes (Chromosome and Complete) for analysis in this study

Species name	Strain	Accession no	GC Content (%)	Genome Length (mb)
**Chromosome index**				
Pseudomonas sp.	02C 26	GCA_002843585.1	61.8	5.4
Pseudomonas sp.	09C 129	GCA_002843625.1	63.1	6.9
Pseudomonas sp.	A214	GCA_900156295.1	60.2	6.1
Pseudomonas sp.	ACM7	GCA_004136015.1	58.7	6.6
Pseudomonas sp.	AO-1	GCA_019775695.1	59.8	6.9
Pseudomonas sp.	B10	GCA_900156235.1	60.0	5.9
Pseudomonas sp.	BIGb0427	GCA_012726325.2	62.7	6.0
Pseudomonas sp.	BS2935	GCA_900105085.1	60.2	5.9
Pseudomonas sp.	BT-42-2	GCA_025847435.1	62.0	5.5
Pseudomonas sp.	BW7P1	GCA_024981055.1	60.5	6.1
Pseudomonas sp.	Colony2	GCA_019382755.1	60.1	7.2
Pseudomonas sp.	IB20	GCA_009707325.1	60.5	5.3
Pseudomonas sp.	IsoF	GCA_023101365.1	62.6	5.9
Pseudomonas sp.	J452	GCA_024666525.1	63.2	4.9
Pseudomonas sp.	LPB260	GCA_013388395.1	65.6	6.4
Pseudomonas sp.	MSPm1	GCA_014109765.1	62.4	5.7
Pseudomonas sp.	MT-1	GCA_000828755.1	60.2	4.9
Pseudomonas sp.	NS1	GCA_002269425.1	60.3	6.7
Pseudomonas sp.	REST10	GCA_029542605.1	65.0	5.1
Pseudomonas sp.	S09G 359	GCA_002843605.1	60.9	6.8
Pseudomonas sp.	SCA2728.1_7	GCA_018138145.1	59.1	6.8
Pseudomonas sp.	SL4(2022)	GCA_026625725.1	59.7	4.4
Pseudomonas sp.	WCS374	GCA_000698295.1	60.0	6.1
Pseudomonas sp.	Y5-11	GCA_022368695.1	59.9	6.5
Pseudomonas sp.	Y39-6	GCA_015888165.1	60.7	6.9
Pseudomonas sp.	Z003-0.4C(8344-21)	GCA_900104975.1	60.0	6.1
**Complete Genome Index**				
Pseudomonas sp.	2hn	GCA_019646095.1	64.7	5.9
Pseudomonas sp.	02-Mar	GCA_019710535.1	60.5	6.4
Pseudomonas sp.	7-41	GCA_021233075.1	61.1	6.8
Pseudomonas sp.	15A4	GCA_016925575.1	60.5	5.7
Pseudomonas sp.	29A	GCA_014117365.1	59.0	6.8
Pseudomonas sp.	31-Dec	GCA_003151075.1	59.1	6.7
Pseudomonas sp.	43A	GCA_013363935.1	59.0	6.8
Pseudomonas sp.	273	GCA_029201425.1	67.5	7.5
Pseudomonas sp.	905_Psudmo1	GCA_029691665.1	62.5	5.5
Pseudomonas sp.	13159349	GCA_013305685.1	62.5	6.0
Pseudomonas sp.	A2	GCA_023100865.1	62.4	6.2
Pseudomonas sp.	A34-9	GCA_029543085.1	59.2	6.5

Pseudomonas sp.	AA-38	GCA_029457475.1	63.3	5.5
Pseudomonas sp.	ABC1	GCA_013395055.1	62.3	4.0
Pseudomonas sp.	ADAK2	GCA_012935755.1	59.6	7.1
Pseudomonas sp.	ADAK7	GCA_012935675.1	59.6	7.1
Pseudomonas sp.	ADAK13	GCA_012935715.1	61.0	7.3
Pseudomonas sp.	ADAK18	GCA_012935695.1	59.2	6.5
Pseudomonas sp.	ADAK20	GCA_012935775.1	60.7	6.0
Pseudomonas sp.	ADAK21	GCA_012935735.1	60.7	6.0
Pseudomonas sp.	ADAK22	GCA_012935655.1	60.7	6.5
Pseudomonas sp.	ADPe	GCA_014843515.1	66.9	7.2
Pseudomonas sp.	AK6U	GCA_002843285.1	65.8	6.9
Pseudomonas sp.	AN-B15	GCA_019056475.1	60.1	6.3
Pseudomonas sp.	ArH3a	GCA_022648765.1	59.9	6.8
Pseudomonas sp.	ATCC 13867	GCA_000349845.1	65.2	5.7
Pseudomonas sp.	ATCC 43928	GCA_005938665.2	59.4	6.5
Pseudomonas sp.	B11D7D	GCA_013410155.2	62.5	5.4
Pseudomonas sp.	B14-6	GCA_013260315.1	59.1	6.8
Pseudomonas sp.	B21-009	GCA_024749345.1	62.8	5.9
Pseudomonas sp.	B21-010	GCA_024749325.1	60.8	6.5
Pseudomonas sp.	B21-012	GCA_024749305.1	62.7	5.9
Pseudomonas sp.	B21-015	GCA_024749285.1	59.0	6.6
Pseudomonas sp.	B21-017	GCA_024749245.1	59.2	6.6
Pseudomonas sp.	B21-019	GCA_024749225.1	59.4	6.4
Pseudomonas sp.	B21-021	GCA_024749205.1	60.6	6.5
Pseudomonas sp.	B21-023	GCA_024749165.1	64.0	5.9
Pseudomonas sp.	B21-028	GCA_024749045.1	60.7	6.5
Pseudomonas sp.	B21-031	GCA_024748985.1	62.4	6.0
Pseudomonas sp.	B21-032	GCA_024748965.1	62.7	5.9
Pseudomonas sp.	B21-035	GCA_024748945.1	62.8	5.9
Pseudomonas sp.	B21-036	GCA_024748925.1	64.3	5.7
Pseudomonas sp.	B21-041	GCA_024748675.1	59.2	6.4
Pseudomonas sp.	B21-044	GCA_024748735.1	64.2	5.7
Pseudomonas sp.	B21-047	GCA_024748775.1	62.6	5.9
Pseudomonas sp.	B21-048	GCA_024748615.1	58.5	5.7
Pseudomonas sp.	B21-051	GCA_024748795.1	60.3	6.0
Pseudomonas sp.	B21-053	GCA_026016365.1	59.5	7.1
Pseudomonas sp.	B21-054	GCA_026016345.1	60.5	6.9
Pseudomonas sp.	B21-056	GCA_026016325.1	60.9	6.4
Pseudomonas sp.	B21-059	GCA_026016285.1	62.3	6.8
Pseudomonas sp.	B111	GCA_026428275.1	66.1	6.7
Pseudomonas sp.	BC42	GCA_021560055.1	63.6	6.9
Pseudomonas sp.	BIOMIG1BAC	GCA_001705995.2	62.1	7.7
Pseudomonas sp.	BJP69	GCA_007633355.1	62.2	5.6
Pseudomonas sp.	Boi14	GCA_018326365.1	63.4	7.0
Pseudomonas sp.	BSw22131	GCA_026810445.1	58.2	5.7
Pseudomonas sp.	BYT-1	GCA_023611805.1	61.9	5.9

Pseudomonas sp.	BYT-5	GCA_023546785.1	61.9	6.0
Pseudomonas sp.	C27(2019)	GCA_008807395.1	48.6	3.2
Pseudomonas sp.	Cab53	GCA_018325965.1	62.2	6.3
Pseudomonas sp.	CBS	GCA_024734645.1	60.3	7.2
Pseudomonas sp.	CBSPAW29	GCA_029215855.1	60.3	7.2
Pseudomonas sp.	CBSPBW29	GCA_029215525.1	60.3	7.2
Pseudomonas sp.	CBSPCAW29	GCA_029215895.1	60.3	7.2
Pseudomonas sp.	CBSPCBW29	GCA_029215915.1	60.3	7.3
Pseudomonas sp.	CBSPCGW29	GCA_029215835.1	60.3	7.2
Pseudomonas sp.	CBSPGW29	GCA_029215815.1	60.3	7.2
Pseudomonas sp.	CC6-YY-74	GCA_002025205.1	61.7	5.0
Pseudomonas sp.	CCOS 191	GCA_001007005.1	64.2	6.0
Pseudomonas sp.	CFA	GCA_014706535.1	61.7	6.1
Pseudomonas sp.	CFSAN084952	GCA_009648855.1	59.9	6.3
Pseudomonas sp.	CIP-10	GCA_020911845.1	61.3	6.5
Pseudomonas sp.	CMR5c	GCA_003850545.1	63.5	6.8
Pseudomonas sp.	CYM-20-01	GCA_023169845.1	60.8	6.7
Pseudomonas sp.	D3	GCA_029320995.1	60.6	6.4
Pseudomonas sp.	DG56-2	GCA_004803755.1	58.7	5.8
Pseudomonas sp.	DNDY-54	GCA_019880365.1	60.2	4.4
Pseudomonas sp.	DR 5-09	GCA_001655595.1	60.5	6.4
Pseudomonas sp.	DR48	GCA_019823085.1	58.9	7.3
Pseudomonas sp.	DR208	GCA_019823005.1	60.9	6.3
Pseudomonas sp.	DTU12.1	GCA_009873475.1	60.7	5.9
Pseudomonas sp.	DTU12.3	GCA_004124275.1	59.5	6.3
Pseudomonas sp.	DY-1	GCA_003626975.1	62.9	5.9
Pseudomonas sp.	Eqa60	GCA_018326405.1	63.3	6.9
Pseudomonas sp.	FDAARGOS_380	GCA_002591235.1	59.7	6.5
Pseudomonas sp.	FDAARGOS_761	GCA_013343155.1	66.4	6.4
Pseudomonas sp.	FGI182	GCA_000511325.1	63.3	5.9
Pseudomonas sp.	FJ2-5-13	GCA_029201605.1	60.2	6.1
Pseudomonas sp.	G2-4	GCA_030064125.1	60.4	6.7
Pseudomonas sp.	G11	GCA_029457515.1	60.4	7.6
Pseudomonas sp.	gcc21	GCA_012844345.1	58.3	4.0
Pseudomonas sp.	GCEP-101	GCA_025133575.1	66.3	6.2
Pseudomonas sp.	GD03721	GCA_029814955.1	62.0	4.7
Pseudomonas sp.	GD03919	GCA_029814935.1	62.0	4.7
Pseudomonas sp.	GM17	GCA_000282175.2	62.8	6.9
Pseudomonas sp.	GOM7	GCA_026723825.1	63.4	5.4
Pseudomonas sp.	GR 6-02	GCA_001655615.1	59.4	6.5
Pseudomonas sp.	GXZC	GCA_026967615.1	60.3	7.3
Pseudomonas sp.	HD6515	GCA_024971855.1	61.3	6.7
Pseudomonas sp.	HLS-6	GCA_002753995.1	59.9	5.3
Pseudomonas sp.	HN2	GCA_020736465.1	60.2	6.2
Pseudomonas sp.	HN2-3	GCA_020691745.1	62.5	6.0
Pseudomonas sp.	HN8-3	GCA_020790195.1	59.9	6.0

Pseudomonas sp.	HN11	GCA_021390155.1	59.7	6.3
Pseudomonas sp.	HS6	GCA_023375815.1	60.1	6.5
Pseudomonas sp.	HS-18	GCA_020401845.1	64.6	6.6
Pseudomonas sp.	I3-I5	GCA_022699445.1	61.6	4.8
Pseudomonas sp.	IAC-BECa141	GCA_020544405.1	60.5	6.2
Pseudomonas sp.	IzPS59	GCA_014863505.1	60.6	6.4
Pseudomonas sp.	J380	GCA_009827115.1	59.7	6.3
Pseudomonas sp.	JS425	GCA_018223845.1	62.1	6.4
Pseudomonas sp.	JY-Q	GCA_001655295.1	61.3	6.2
Pseudomonas sp.	KBS0707	GCA_005937945.2	57.9	6.1
Pseudomonas sp.	KBS0802	GCA_005937845.2	61.5	6.3
Pseudomonas sp.	KHPS1	GCA_024205205.1	64.4	5.1
Pseudomonas sp.	KNUC1026	GCA_021650705.1	63.3	5.2
Pseudomonas sp.	KU26590	GCA_026153515.1	60.2	6.2
Pseudomonas sp.	KUIN-1	GCA_009176725.1	59.2	6.0
Pseudomonas sp.	L5B5	GCA_020520285.1	63.2	6.8
Pseudomonas sp.	LBUM920	GCA_003852315.1	60.7	6.5
Pseudomonas sp.	Leaf58	GCA_003627215.1	61.3	6.3
Pseudomonas sp.	LG1D9	GCA_002949995.1	60.5	6.3
Pseudomonas sp.	LG1E9	GCA_003290225.1	60.2	6.2
Pseudomonas sp.	LH1G9	GCA_002949975.1	61.0	6.5
Pseudomonas sp.	LPH1	GCA_002037565.1	62.7	5.2
Pseudomonas sp.	LRP2-20	GCA_024349685.1	62.9	6.0
Pseudomonas sp.	LS.1a	GCA_022533585.1	63.1	5.7
Pseudomonas sp.	LS44	GCA_024730785.1	62.4	4.5
Pseudomonas sp.	LS1212	GCA_024741815.1	60.2	5.7
Pseudomonas sp.	LTGT-11-2Z	GCA_003691445.1	61.7	6.1
Pseudomonas sp.	LTJR-52	GCA_003691465.1	55.2	5.5
Pseudomonas sp.	Lz4W	GCA_000346225.2	58.7	5.0
Pseudomonas sp.	M1	GCA_022760135.1	67.3	7.0
Pseudomonas sp.	M2(2023)	GCA_030263955.1	62.7	5.7
Pseudomonas sp.	M30-35	GCA_002163625.1	54.3	4.9
Pseudomonas sp.	Marseille-Q3773	GCA_916618955.1	63.2	5.6
Pseudomonas sp.	MM211	GCA_020386635.1	61.5	5.3
Pseudomonas sp.	MM213	GCA_020423045.1	59.4	6.7
Pseudomonas sp.	MM221	GCA_947090825.1	61.9	6.7
Pseudomonas sp.	MM223	GCA_947090765.1	61.9	6.7
Pseudomonas sp.	MM227	GCA_947090715.1	62.2	4.6
Pseudomonas sp.	MPC6	GCA_006094435.1	59.8	7.2
Pseudomonas sp.	MPDS	GCA_013283895.1	60.3	6.2
Pseudomonas sp.	MPFS	GCA_022537035.1	62.3	7.1
Pseudomonas sp.	MRSN12121	GCA_000931465.1	63.7	7.0
Pseudomonas sp.	MTM4	GCA_019355055.1	60.7	4.7
Pseudomonas sp.	MYb193	GCA_002966775.1	59.9	6.2
Pseudomonas sp.	N3-W	GCA_024970185.1	59.7	6.7
Pseudomonas sp.	NC02	GCA_002874965.1	61.1	6.9

Pseudomonas sp.	NEEL19	GCA_028751975.1	62.7	5.3
Pseudomonas sp.	NIBRBAC000502773	GCA_006517195.1	61.3	6.7
Pseudomonas sp.	NIBR-H-19	GCA_021228675.1	58.6	6.2
Pseudomonas sp.	NY5710	GCA_013305665.1	62.3	5.6
Pseudomonas sp.	NY11382	GCA_027595145.1	62.2	6.3
Pseudomonas sp.	NyZ480	GCA_029537255.1	61.9	5.4
Pseudomonas sp.	OE 28.3	GCA_014268205.2	60.0	6.6
Pseudomonas sp.	OIL-1	GCA_010669185.1	58.3	4.2
Pseudomonas sp.	Os17	GCA_001547895.1	63.5	6.9
Pseudomonas sp.	Ost2	GCA_018326065.1	62.9	7.4
Pseudomonas sp.	OST1909	GCA_015277575.1	59.6	6.3
Pseudomonas sp.	p1(2021b)	GCA_020151015.1	63.1	5.6
Pseudomonas sp.	P13	GCA_029952805.1	60.4	5.9
Pseudomonas sp.	Pc102	GCA_018408275.1	66.8	6.7
Pseudomonas sp.	PDNC002	GCA_016919445.1	64.9	6.4
Pseudomonas sp.	phDV1	GCA_003408635.1	62.3	4.7
Pseudomonas sp.	PIA16	GCA_023509015.1	62.0	6.7
Pseudomonas sp.	PMCC200344	GCA_030053095.1	58.6	6.5
Pseudomonas sp.	PMCC200367	GCA_030053115.1	58.7	6.4
Pseudomonas sp.	PONIH3	GCA_002934345.1	63.4	6.3
Pseudomonas sp.	PP3	GCA_905336995.1	59.2	6.4
Pseudomonas sp.	PS1(2021)	GCA_020405165.1	65.5	7.8
Pseudomonas sp.	PSE14	GCA_029203285.1	65.2	6.0
Pseudomonas sp.	PSKL.D1	GCA_028898945.1	61.7	5.9
Pseudomonas sp.	Q1-7	GCA_028010285.1	64.8	5.8
Pseudomonas sp.	R1-43-08	GCA_003852255.1	60.6	5.6
Pseudomonas sp.	R2-7-07	GCA_003852355.1	60.4	5.8
Pseudomonas sp.	R2-37-08W	GCA_003851705.1	60.4	5.8
Pseudomonas sp.	R2-60-08W	GCA_003852335.1	60.4	5.8
Pseudomonas sp.	R3-18-08	GCA_003852065.1	60.4	5.8
Pseudomonas sp.	R3-52-08	GCA_003851745.1	60.3	5.9
Pseudomonas sp.	R4-34-07	GCA_003852375.1	60.4	5.9
Pseudomonas sp.	R4-35-07	GCA_003852235.1	60.3	5.8
Pseudomonas sp.	R4-39-08	GCA_003852275.1	60.4	5.9
Pseudomonas sp.	R5-89-07	GCA_003851685.1	60.6	6.0
Pseudomonas sp.	R11-23-07	GCA_003852295.1	60.6	5.7
Pseudomonas sp.	R32	GCA_009866705.1	62.4	5.6
Pseudomonas sp.	R76	GCA_009834565.1	59.9	6.8
Pseudomonas sp.	R84	GCA_009834515.1	59.1	6.6
Pseudomonas sp.	RC3H12	GCA_018596375.1	64.0	5.8
Pseudomonas sp.	RtIB026	GCA_016861355.2	64.6	5.9
Pseudomonas sp.	RU47	GCA_004011755.1	59.2	6.7
Pseudomonas sp.	S04	GCA_009834545.1	60.9	6.1
Pseudomonas sp.	S06B 330	GCA_002845275.2	59.6	5.6
Pseudomonas sp.	S07E 245	GCA_019599025.1	61.9	5.4
Pseudomonas sp.	S11A 273	GCA_019599005.1	59.6	5.7

Pseudomonas sp.	S19	GCA_009866725.1	60.9	6.1
Pseudomonas sp.	S34	GCA_009866745.1	59.8	6.3
Pseudomonas sp.	S35	GCA_009866765.1	60.1	6.6
Pseudomonas sp.	S49	GCA_009866785.1	59.2	6.7
Pseudomonas sp.	S150	GCA_004526365.1	60.2	6.3
Pseudomonas sp.	SC3(2021)	GCA_019856535.1	60.2	6.3
Pseudomonas sp.	SCB32	GCA_009189165.1	64.6	6.3
Pseudomonas sp.	SDM007	GCA_016925675.1	60.1	6.3
Pseudomonas sp.	Seg1	GCA_018326005.1	59.2	6.6
Pseudomonas sp.	SGAir0191	GCA_002812565.2	61.4	5.1
Pseudomonas sp.	Shine	GCA_001511755.1	59.2	6.4
Pseudomonas sp.	SK	GCA_012975065.1	63.3	5.7
Pseudomonas sp.	SK2	GCA_016772395.1	61.9	5.5
Pseudomonas sp.	SK3(2021)	GCA_016772375.1	63.0	6.5
Pseudomonas sp.	So3.2b	GCA_019464415.1	60.5	6.2
Pseudomonas sp.	SO81	GCA_030374125.1	65.4	4.8
Pseudomonas sp.	SORT22	GCA_018417635.1	62.9	5.8
Pseudomonas sp.	St29	GCA_001547915.1	63.3	6.8
Pseudomonas sp.	St290	GCA_018325805.1	60.8	6.8
Pseudomonas sp.	St316	GCA_018325905.1	60.5	6.8
Pseudomonas sp.	St386	GCA_018325865.1	60.8	6.8
Pseudomonas sp.	StFLB209	GCA_000829415.1	60.7	6.3
Pseudomonas sp.	SW-3	GCA_016615645.1	59.4	6.5
Pseudomonas sp.	SWI6	GCA_002947975.1	61.8	5.7
Pseudomonas sp.	SWI7	GCA_006351945.1	61.6	4.9
Pseudomonas sp.	SWI36	GCA_002948105.1	61.8	6.2
Pseudomonas sp.	SWI44	GCA_002947915.1	61.8	5.9
Pseudomonas sp.	SXM-1	GCA_004379315.1	60.8	7.2
Pseudomonas sp.	T8	GCA_024610635.1	62.6	7.2
Pseudomonas sp.	TCU-HL1	GCA_001708505.1	63.2	6.3
Pseudomonas sp.	THAF42	GCA_009363475.1	64.8	5.3
Pseudomonas sp.	THAF187a	GCA_009363395.1	64.8	5.3
Pseudomonas sp.	TKP	GCA_000508205.1	60.5	7.0
Pseudomonas sp.	TMW 2.1634	GCA_001703595.1	58.9	5.7
Pseudomonas sp.	Tri1	GCA_017968885.1	60.5	6.7
Pseudomonas sp.	UW4	GCA_000316175.1	60.1	6.2
Pseudomonas sp.	VLB120	GCA_000494915.1	61.5	6.0
Pseudomonas sp.	WJP1	GCA_028471945.1	60.3	7.0
Pseudomonas sp.	XWY-1	GCA_002953115.1	61.5	6.3
Pseudomonas sp.	Y39-6	GCA_023651955.1	60.8	7.0
Pseudomonas sp.	Yellow	GCA_001511775.1	61.7	4.8
Pseudomonas sp.	YeP6b	GCA_025642995.1	59.7	6.7
Pseudomonas sp.	Z8(2022)	GCA_025837155.1	62.9	4.5
Pseudomonas sp.	ZM23	GCA_026686735.1	64.0	6.2

Table S2
List of downloaded 1197 *Pseudomonas* spp. genomes (Contigs) to analyze in this study

Species name	Strain	Accession no	GC Content (%)	Genome Length (mb)
Pseudomonas sp.	2FE	GCA_005503735.1	62.1	4.1
Pseudomonas sp.	2FG	GCA_005502935.1	62.5	4.2
Pseudomonas sp.	5P_3.1_Bac2	GCA_025465995.1	59.0	4.7
Pseudomonas sp.	008	GCA_016861935.1	59.1	6.9
Pseudomonas sp.	11/12A	GCA_000800055.1	59.6	6.8
Pseudomonas sp.	13-2922	GCA_023242435.1	61.3	7.2
Pseudomonas sp.	14A	GCA_018132745.1	60.0	6.0
Pseudomonas sp.	20S_6.2_Bac1	GCA_025465975.1	61.4	7.5
Pseudomonas sp.	21TX0197	GCA_028307245.1	61.7	6.3
Pseudomonas sp.	29	GCA_002754355.1	59.3	6.5
Pseudomonas sp.	43NM1	GCA_002836905.1	59.5	6.1
Pseudomonas sp.	75	GCA_030016165.1	63.6	6.8
Pseudomonas sp.	250J	GCA_001259595.1	64.7	5.9
Pseudomonas sp.	681	GCA_029955235.1	59.8	6.7
Pseudomonas sp.	770NI	GCA_004210235.1	60.2	5.9
Pseudomonas sp.	1079	GCA_016925755.1	60.2	5.9
Pseudomonas sp.	1239	GCA_002157495.1	64.0	6.0
Pseudomonas sp.	2822-15	GCA_002742515.1	59.8	6.7
Pseudomonas sp.	2822-17	GCA_002742485.1	59.3	6.5
Pseudomonas sp.	2995-1 (Jan 95)	GCA_002742505.1	59.1	6.4
Pseudomonas sp.	2995-3 (Mar 95)	GCA_002742565.1	59.8	6.5
Pseudomonas sp.	24516wA6_ntrB -del410-421	GCA_020093335.1	60.5	6.7
Pseudomonas sp.	24516wB5_ntrBA289C	GCA_020093345.1	60.5	6.7
Pseudomonas sp.	24516wB6_glnK-del84	GCA_020093375.1	60.5	6.7
Pseudomonas sp.	17023526	GCA_024718355.1	66.2	6.8
Pseudomonas sp.	17023671	GCA_024714085.1	66.2	6.8
Pseudomonas sp.	17033095	GCA_024714025.1	66.1	6.7
Pseudomonas sp.	17053182	GCA_024714065.1	66.1	6.8
Pseudomonas sp.	17053418	GCA_024718335.1	65.9	7.1
Pseudomonas sp.	17053703	GCA_024714105.1	66.5	6.3
Pseudomonas sp.	17063399	GCA_024718315.1	66.1	6.9
Pseudomonas sp.	17072548	GCA_024714045.1	66.1	6.9
Pseudomonas sp.	17073326	GCA_024714155.1	66.2	6.4
Pseudomonas sp.	17102422	GCA_024714005.1	66.5	6.3
Pseudomonas sp.	17103552	GCA_024718395.1	65.9	7.0
Pseudomonas sp.	17104299	GCA_024714125.1	66.4	6.4
Pseudomonas sp.	18073667	GCA_024714305.1	66.1	7.0
Pseudomonas sp.	18082547	GCA_024714345.1	66.0	6.9
Pseudomonas sp.	18082551	GCA_024718465.1	66.0	6.8
Pseudomonas sp.	18082574	GCA_024718505.1	65.6	7.0

Pseudomonas sp.	18083194	GCA_024714255.1	66.3	6.6
Pseudomonas sp.	18083202	GCA_024714225.1	66.0	7.0
Pseudomonas sp.	18083259	GCA_024714235.1	66.2	6.6
Pseudomonas sp.	18083286	GCA_024714245.1	66.4	6.4
Pseudomonas sp.	18084127	GCA_024714165.1	66.0	7.1
Pseudomonas sp.	18092229	GCA_024718485.1	66.0	6.8
Pseudomonas sp.	18093371	GCA_024714315.1	65.9	6.3
Pseudomonas sp.	18101001-2	GCA_024714145.1	65.6	7.0
Pseudomonas sp.	18102011	GCA_024718435.1	65.6	7.0
Pseudomonas sp.	18103014	GCA_024714205.1	65.9	6.9
Pseudomonas sp.	18113298	GCA_024714365.1	66.5	6.3
Pseudomonas sp.	19062259	GCA_024714385.1	66.1	6.3
Pseudomonas sp.	19064969	GCA_024714425.1	66.1	6.7
Pseudomonas sp.	19072337-2	GCA_024714405.1	66.1	6.8
Pseudomonas sp.	19082381	GCA_024714445.1	66.1	6.8
Pseudomonas sp.	A-1	GCA_004801855.1	68.2	4.4
Pseudomonas sp.	A-B-26	GCA_019956595.1	58.9	6.5
Pseudomonas sp.	ABFPK	GCA_001586765.1	62.2	6.2
Pseudomonas sp.	ACN5	GCA_002303975.1	59.9	6.7
Pseudomonas sp.	AF03-9	GCA_020515815.1	61.2	6.0
Pseudomonas sp.	AG1028	GCA_007993955.1	63.2	5.0
Pseudomonas sp.	ALS1131	GCA_007049925.1	62.4	5.6
Pseudomonas sp.	ALS1279	GCA_007049955.1	62.5	5.3
Pseudomonas sp.	AN3A02	GCA_011765255.1	60.5	6.8
Pseudomonas sp.	ANT_H4	GCA_008369295.1	58.6	6.1
Pseudomonas sp.	ANT_H12B	GCA_008369325.1	58.6	6.3
Pseudomonas sp.	ANT_J12	GCA_008370045.1	59.8	7.2
Pseudomonas sp.	Ant30-3	GCA_000690905.1	58.6	6.1
Pseudomonas sp.	AOB-7	GCA_003696305.1	66.6	4.9
Pseudomonas sp.	AP42	GCA_001728935.1	59.8	6.8
Pseudomonas sp.	AS2.8	GCA_014190935.1	66.3	4.9
Pseudomonas sp.	ATCC 13985	GCA_006322025.2	62.7	7.0
Pseudomonas sp.	ATCC PTA-122608	GCA_001952855.1	60.2	7.0
Pseudomonas sp.	AU11048	GCA_001806145.1	58.8	5.1
Pseudomonas sp.	Au-Pse12	GCA_021341665.1	63.2	7.0
Pseudomonas sp.	B1(2017)	GCA_002113135.1	60.7	6.2
Pseudomonas sp.	B1	GCA_003097365.1	60.3	6.7
Pseudomonas sp.	B2M1-30	GCA_025447575.1	61.1	6.4
Pseudomonas sp.	B14	GCA_012030485.1	60.3	6.7
Pseudomonas sp.	B20	GCA_020515755.1	59.4	5.8
Pseudomonas sp.	B21(2017)	GCA_002113735.1	60.7	6.2
Pseudomonas sp.	B25(2017)	GCA_002113705.1	60.8	6.2
Pseudomonas sp.	B27(2017)	GCA_002113055.1	60.8	6.2
Pseudomonas sp.	B29(2017)	GCA_002112985.1	60.7	6.2
Pseudomonas sp.	B31(2017)	GCA_002112965.1	60.7	6.4
Pseudomonas sp.	B33(2017)	GCA_002113655.1	60.7	6.2

Pseudomonas sp.	B36(2017)	GCA_002112905.1	60.7	6.4
Pseudomonas sp.	B37(2017)	GCA_002112885.1	60.7	6.4
Pseudomonas sp.	B39(2017)	GCA_002112835.1	60.7	6.4
Pseudomonas sp.	BAV 4579	GCA_009765395.1	65.4	5.5
Pseudomonas sp.	BAY1663	GCA_000582595.1	65.0	4.9
Pseudomonas sp.	BCA-13	GCA_004519435.1	60.2	5.9
Pseudomonas sp.	BCA-14	GCA_004519425.1	60.2	6.3
Pseudomonas sp.	BCA-17	GCA_004519405.1	60.2	6.3
Pseudomonas sp.	Bc-h	GCA_002080045.1	59.5	7.0
Pseudomonas sp.	BF-B-15	GCA_019946675.1	59.6	6.4
Pseudomonas sp.	BF-B-18	GCA_019947205.1	59.5	6.7
Pseudomonas sp.	BF-R-01	GCA_019946615.1	59.5	6.8
Pseudomonas sp.	BF-R-05	GCA_019946655.1	59.8	6.3
Pseudomonas sp.	BF-R-26	GCA_019947395.1	58.9	6.6
Pseudomonas sp.	BF-RE-01	GCA_019946585.1	59.6	6.3
Pseudomonas sp.	BF-RE-02	GCA_019947175.1	59.6	6.3
Pseudomonas sp.	BF-RE-14	GCA_019955755.1	59.5	6.7
Pseudomonas sp.	Bi123	GCA_918697945.1	59.4	6.4
Pseudomonas sp.	Bi130	GCA_918697935.1	59.4	6.6
Pseudomonas sp.	BIGb0164	GCA_024810135.1	61.4	6.7
Pseudomonas sp.	BIGb0176	GCA_024810175.1	63.5	6.9
Pseudomonas sp.	BIGb0278	GCA_024809895.1	62.0	5.3
Pseudomonas sp.	BIGb0381	GCA_024809815.1	60.6	6.5
Pseudomonas sp.	BIGb0408	GCA_025961395.1	62.2	6.0
Pseudomonas sp.	BJa3	GCA_026343815.1	63.8	5.9
Pseudomonas sp.	BJa5	GCA_030272875.1	64.9	6.9
Pseudomonas sp.	BLCC-B13	GCA_028550635.1	65.4	4.5
Pseudomonas sp.	BMW13	GCA_013357275.1	65.0	5.2
Pseudomonas sp.	BP6	GCA_017875935.1	61.6	5.9
Pseudomonas sp.	BP7	GCA_017875875.1	61.6	6.0
Pseudomonas sp.	BP8	GCA_017875955.1	61.8	6.0
Pseudomonas sp.	BS3759	GCA_900103395.1	59.2	6.5
Pseudomonas sp.	BS3767	GCA_900099665.1	59.1	6.1
Pseudomonas sp.	CBZ-4	GCA_000346755.1	61.2	6.5
Pseudomonas sp.	CDFA 610	GCA_021147865.1	59.1	5.8
Pseudomonas sp.	CFBP2511	GCA_030209255.1	58.7	6.2
Pseudomonas sp.	CFBP8770	GCA_014841705.1	62.1	4.7
Pseudomonas sp.	CFBP8772	GCA_014841665.1	60.5	5.9
Pseudomonas sp.	CFBP8773	GCA_014841625.1	62.1	4.7
Pseudomonas sp.	CFBP13506	GCA_005233685.1	60.8	5.9
Pseudomonas sp.	CFBP13508	GCA_005233515.1	60.0	6.0
Pseudomonas sp.	CFBP13509	GCA_005233485.1	60.9	6.8
Pseudomonas sp.	CFBP13602	GCA_014842725.1	62.2	4.8
Pseudomonas sp.	CFBP13710	GCA_014842235.1	62.2	4.7
Pseudomonas sp.	CFBP13711	GCA_014842265.1	60.2	6.2
Pseudomonas sp.	CFBP13715	GCA_014842125.1	60.2	6.2

Pseudomonas sp.	CFBP13719	GCA_014842095.1	60.6	5.7
Pseudomonas sp.	CFBP13727	GCA_014841915.1	62.4	4.6
Pseudomonas sp.	CFII64	GCA_000416235.1	58.9	6.4
Pseudomonas sp.	CFT9	GCA_000416255.1	59.8	6.2
Pseudomonas sp.	Choline-02u-1	GCA_002836515.1	59.7	6.0
Pseudomonas sp.	Choline-3u-10	GCA_002836195.1	60.3	5.0
Pseudomonas sp.	ChxA	GCA_030263855.1	58.9	8.0
Pseudomonas sp.	CMR5c	GCA_001269545.1	63.6	6.8
Pseudomonas sp.	CNPSo 3701	GCA_028674975.1	63.1	5.0
Pseudomonas sp.	CRE Jenny 4	GCA_030369035.1	60.2	5.9
Pseudomonas sp.	CROZ-RG-20F-R04-06	GCA_022662355.1	59.2	6.7
Pseudomonas sp.	CROZ-RG-20F-R04-15	GCA_022662375.1	59.2	6.7
Pseudomonas sp.	CVAP#3	GCA_018986375.1	58.7	6.3
Pseudomonas sp.	D1HM	GCA_019266105.1	58.4	4.9
Pseudomonas sp.	DCB_BG	GCA_026241005.1	61.9	6.0
Pseudomonas sp.	DCB_BI	GCA_026240995.1	62.1	5.9
Pseudomonas sp.	DE0039	GCA_007680285.1	61.7	4.9
Pseudomonas sp.	DP16D-T1	GCA_017351905.1	61.7	6.6
Pseudomonas sp.	DP-17	GCA_022380625.1	64.9	6.5
Pseudomonas sp.	dw_358	GCA_018449655.1	62.0	5.5
Pseudomonas sp.	dw_612	GCA_018449725.1	59.7	7.4
Pseudomonas sp.	Eb3	GCA_024385385.1	62.2	6.0
Pseudomonas sp.	ef1	GCA_007293365.1	59.7	6.2
Pseudomonas sp.	efr-133-R2A-59	GCA_030209265.1	58.9	6.2
Pseudomonas sp.	efr-133-TYG-5	GCA_030209175.1	62.2	6.1
Pseudomonas sp.	efr-133-TYG-103a	GCA_030209235.1	60.4	6.1
Pseudomonas sp.	EggHat1	GCA_014892995.1	62.5	5.3
Pseudomonas sp.	EMN2	GCA_009650555.1	63.8	5.8
Pseudomonas sp.	EYE_354	GCA_023148575.1	60.7	5.7
Pseudomonas sp.	F(2018)	GCA_005508865.1	65.6	4.7
Pseudomonas sp.	FFUP_PS_41	GCA_002858645.1	62.2	6.5
Pseudomonas sp.	FH4	GCA_000510915.2	60.1	6.0
Pseudomonas sp.	Fig-3	GCA_006151705.1	60.2	6.2
Pseudomonas sp.	Fl4BN1	GCA_009908285.1	61.5	7.3
Pseudomonas sp.	Fl5BN2	GCA_009908205.1	61.5	7.2
Pseudomonas sp.	fls2-241-R2A-110	GCA_030209165.1	59.0	6.8
Pseudomonas sp.	FME51	GCA_014897305.1	54.2	4.8
Pseudomonas sp.	FR229a	GCA_030209115.1	60.2	5.9
Pseudomonas sp.	FSL A6-1183	GCA_009600565.1	57.9	5.5
Pseudomonas sp.	FSL R10-0399	GCA_009600165.1	57.6	6.3
Pseudomonas sp.	FSL R10-1339	GCA_009600545.1	58.0	5.2
Pseudomonas sp.	FSL R10-2398	GCA_009600285.1	57.9	6.0
Pseudomonas sp.	FSL W7-0098	GCA_005930585.1	58.3	5.0
Pseudomonas sp.	FW215-L1	GCA_017351895.1	60.3	6.5
Pseudomonas sp.	FW215-L2	GCA_002882795.1	60.9	7.3
Pseudomonas sp.	FW215-R2	GCA_002882455.1	60.3	6.5

Pseudomonas sp.	FW300-N1A5	GCA_017351755.1	58.8	6.3
Pseudomonas sp.	FW300-N1B4	GCA_017351775.1	58.8	6.6
Pseudomonas sp.	FW300-N2A2	GCA_017351735.1	59.7	6.4
Pseudomonas sp.	FW300-N2C3	GCA_017351715.1	61.3	7.1
Pseudomonas sp.	FW300-N2E2	GCA_017351695.1	60.5	6.9
Pseudomonas sp.	FW300-N2E3	GCA_017351665.1	58.8	6.4
Pseudomonas sp.	FW300-N2F2	GCA_017351655.1	61.1	7.1
Pseudomonas sp.	FW301-21B01	GCA_002882605.1	60.4	7.1
Pseudomonas sp.	FW301-21B01	GCA_017351635.1	60.4	7.1
Pseudomonas sp.	FW301-21D1A	GCA_017351585.1	60.3	7.1
Pseudomonas sp.	FW305-3-2-15-C-TSA2	GCA_002882915.1	60.8	6.6
Pseudomonas sp.	FW305-28	GCA_017351395.1	61.3	7.0
Pseudomonas sp.	FW306-02-H05-AB	GCA_017350695.1	58.8	6.3
Pseudomonas sp.	FW306-2-2C-D06B	GCA_002883535.1	61.7	6.7
Pseudomonas sp.	FW306-2-11AA	GCA_017350655.1	58.8	6.3
Pseudomonas sp.	FW306-2-11AB	GCA_017350615.1	58.8	6.2
Pseudomonas sp.	FW507-14TSA	GCA_017350515.1	63.6	6.9
Pseudomonas sp.	GD03746	GCA_029843065.1	61.6	5.7
Pseudomonas sp.	GD03842	GCA_029841165.1	60.3	5.8
Pseudomonas sp.	GD03903	GCA_029840045.1	64.8	6.9
Pseudomonas sp.	GD03944	GCA_029839275.1	64.8	5.1
Pseudomonas sp.	GD04019	GCA_029837695.1	65.0	4.8
Pseudomonas sp.	GD04042	GCA_029837175.1	66.4	5.4
Pseudomonas sp.	GD04091	GCA_029836165.1	64.7	5.8
Pseudomonas sp.	GD04158	GCA_029834875.1	64.6	5.0
Pseudomonas sp.	GGS8	GCA_024168645.1	59.0	6.7
Pseudomonas sp.	GL-R-26	GCA_019947365.1	59.9	6.4
Pseudomonas sp.	GM_Psu_1	GCA_026544185.1	66.0	5.0
Pseudomonas sp.	GM_Psu_2	GCA_026541025.1	66.0	5.3
Pseudomonas sp.	GM21	GCA_000282215.1	58.5	6.6
Pseudomonas sp.	GM30	GCA_000282275.2	60.3	6.2
Pseudomonas sp.	GM33	GCA_000282295.1	60.1	6.7
Pseudomonas sp.	GM41(2012)	GCA_000282315.2	59.0	6.7
Pseudomonas sp.	GM48	GCA_000282335.1	59.3	6.4
Pseudomonas sp.	GM60	GCA_000282415.1	59.6	6.4
Pseudomonas sp.	GM78	GCA_000282475.1	60.2	7.3
Pseudomonas sp.	GM80	GCA_000282515.1	59.2	6.8
Pseudomonas sp.	GOM6	GCA_029537485.1	62.8	4.3
Pseudomonas sp.	GP01-A3	GCA_017350395.1	60.9	7.3
Pseudomonas sp.	GP01-A3	GCA_017350395.1	60.9	7.3
Pseudomonas sp.	GP01-A6	GCA_002881835.1	60.9	7.4
Pseudomonas sp.	GP01-A8	GCA_002881675.1	60.9	7.4
Pseudomonas sp.	GP01-A8	GCA_017350315.1	60.9	7.3
Pseudomonas sp.	GP01-A9	GCA_002881645.1	60.8	7.4
Pseudomonas sp.	GP01-A9	GCA_017350235.1	60.9	7.3
Pseudomonas sp.	GP01-A11	GCA_002882805.1	60.9	7.4

Pseudomonas sp.	GP01-A13	GCA_002881755.1	60.9	7.4
Pseudomonas sp.	GP01-A15	GCA_002882705.1	60.9	7.4
Pseudomonas sp.	GTC 16473	GCA_001753855.1	61.8	6.2
Pseudomonas sp.	GTC 16481	GCA_001753875.1	62.5	5.4
Pseudomonas sp.	GTC 16482	GCA_001319995.1	61.5	6.8
Pseudomonas sp.	GV071	GCA_003050835.1	61.4	5.7
Pseudomonas sp.	GW101-3H06	GCA_017350295.1	58.9	6.7
Pseudomonas sp.	GW456-11-11-14-LB1	GCA_017350255.1	58.8	6.2
Pseudomonas sp.	GW456-L13	GCA_017350115.1	60.0	5.9
Pseudomonas sp.	GW456-L15	GCA_017350055.1	63.3	7.0
Pseudomonas sp.	GW460-12	GCA_017349855.1	60.4	7.4
Pseudomonas sp.	GW460-12	GCA_002882595.1	60.3	7.4
Pseudomonas sp.	GW460-12-1-14-TSB6	GCA_017349795.1	59.3	6.8
Pseudomonas sp.	GW460-E12	GCA_017349695.1	59.2	7.1
Pseudomonas sp.	GW704-E3	GCA_017349575.1	60.9	6.6
Pseudomonas sp.	GX19020	GCA_023371075.1	62.7	4.9
Pseudomonas sp.	H1h	GCA_000633255.1	60.3	6.4
Pseudomonas sp.	H9	GCA_004353925.1	59.8	5.9
Pseudomonas sp.	HAR-UPW-AIA-41	GCA_002304225.1	62.3	3.7
Pseudomonas sp.	HB05	GCA_016756915.1	61.1	5.9
Pseudomonas sp.	HP2	GCA_006386585.1	68.8	4.6
Pseudomonas sp.	HUK17	GCA_001566765.1	65.5	2.6
Pseudomonas sp.	IB20	GCA_002263605.1	59.8	6.5
Pseudomonas sp.	IC_126	GCA_004331835.1	60.8	4.6
Pseudomonas sp.	ICBG1301	GCA_016918385.1	60.5	6.1
Pseudomonas sp.	ICMP 564	GCA_002699785.1	60.1	6.1
Pseudomonas sp.	ICMP 19500	GCA_001467145.1	59.9	6.4
Pseudomonas sp.	ID233	GCA_028657135.1	60.7	6.4
Pseudomonas sp.	ID357	GCA_028657095.1	62.1	6.9
Pseudomonas sp.	ID387	GCA_028657035.1	60.8	6.3
Pseudomonas sp.	ID386	GCA_028657055.1	60.8	6.4
Pseudomonas sp.	ID642	GCA_028656995.1	59.2	6.6
Pseudomonas sp.	ID1044	GCA_028656895.1	59.1	6.5
Pseudomonas sp.	ID1048	GCA_028656875.1	62.4	6.6
Pseudomonas sp.	ISL-88	GCA_018612965.1	45.2	3.2
Pseudomonas sp.	ITA	GCA_029953685.1	60.0	6.6
Pseudomonas sp.	J237	GCA_001749735.1	57.2	5.2
Pseudomonas sp.	JM0905a	GCA_014700075.1	64.0	5.8
Pseudomonas sp.	JMN-1	GCA_004519345.1	60.2	6.3
Pseudomonas sp.	JR33AA	GCA_021378985.1	62.0	5.4
Pseudomonas sp.	JUb42	GCA_024807945.1	59.7	7.4
Pseudomonas sp.	JUb96	GCA_025961295.1	61.6	5.7
Pseudomonas sp.	JV241A	GCA_900275665.1	62.5	5.9
Pseudomonas sp.	KB-10	GCA_018221585.1	63.0	5.2
Pseudomonas sp.	KBS0710	GCA_005938045.2	60.1	6.6
Pseudomonas sp.	KCA11	GCA_021378935.1	63.1	5.6

Pseudomonas sp.	KK4	GCA_001984065.1	60.8	6.4
Pseudomonas sp.	LA21	GCA_022819105.1	65.3	6.0
Pseudomonas sp.	LAM2023	GCA_016620375.1	63.3	5.4
Pseudomonas sp.	LD120	GCA_009861525.1	61.6	6.7
Pseudomonas sp.	Leaf15	GCA_001421425.1	60.2	6.6
Pseudomonas sp.	Leaf58	GCA_001422615.1	61.4	6.3
Pseudomonas sp.	Leaf59	GCA_920984685.1	61.4	5.3
Pseudomonas sp.	Leaf98	GCA_003258395.1	60.2	6.5
Pseudomonas sp.	Leaf434	GCA_001425545.1	60.1	5.9
Pseudomonas sp.	LF19	GCA_021378965.1	62.6	5.8
Pseudomonas sp.	LLC-1	GCA_002992185.1	62.4	5.9
Pseudomonas sp.	LM20	GCA_021378925.1	63.1	5.6
Pseudomonas sp.	LY10J	GCA_015620965.1	59.5	4.9
Pseudomonas sp.	lyk4-40-TSB-59a	GCA_030209135.1	60.6	6.4
Pseudomonas sp.	lyk4-R2A-8	GCA_030209095.1	61.1	5.5
Pseudomonas sp.	lyk4-R2A-10	GCA_030209075.1	60.7	6.3
Pseudomonas sp.	lyk4-TYG-107	GCA_030209055.1	60.2	6.1
Pseudomonas sp.	M2	GCA_015751845.1	61.8	5.7
Pseudomonas sp.	M5	GCA_016907075.1	61.9	5.4
Pseudomonas sp.	MAG002Y	GCA_019400845.1	55.4	5.5
Pseudomonas sp.	Marseille-P8916	GCA_916618875.1	64.6	5.4
Pseudomonas sp.	Marseille-P9655	GCA_916618925.1	61.9	6.3
Pseudomonas sp.	Marseille-P9899	GCA_903166565.1	62.9	7.1
Pseudomonas sp.	Marseille-Q0931	GCA_940789645.1	62.8	5.2
Pseudomonas sp.	Marseille-Q5115	GCA_916618905.1	61.8	5.5
Pseudomonas sp.	Marseille-Q5117	GCA_946901175.1	59.8	6.2
Pseudomonas sp.	Marseille-Q5299	GCA_943737035.1	61.9	5.5
Pseudomonas sp.	MDMC216	GCA_003269355.2	62.7	5.5
Pseudomonas sp.	MDMC224	GCA_003269315.1	62.5	5.6
Pseudomonas sp.	MEB105	GCA_030209015.1	60.4	6.1
Pseudomonas sp.	MF2846	GCA_016405325.1	60.1	6.2
Pseudomonas sp.	MF2857	GCA_016613045.1	60.1	6.2
Pseudomonas sp.	MF4836	GCA_002018875.1	62.0	7.5
Pseudomonas sp.	MF5691	GCA_016405645.1	60.4	6.2
Pseudomonas sp.	MF6747	GCA_016613205.1	60.0	6.8
Pseudomonas sp.	MF6754	GCA_016613195.1	60.3	6.3
Pseudomonas sp.	MF6755	GCA_016405635.1	59.5	6.6
Pseudomonas sp.	MF6767	GCA_016405475.1	59.7	6.5
Pseudomonas sp.	MF6772	GCA_016405505.1	60.7	6.2
Pseudomonas sp.	MF6776	GCA_016613125.1	58.5	6.7
Pseudomonas sp.	MF6784	GCA_016405585.1	60.7	6.0
Pseudomonas sp.	MF6787	GCA_016405525.1	60.3	6.3
Pseudomonas sp.	MF7451	GCA_016405175.1	59.9	6.3
Pseudomonas sp.	MF7453	GCA_016405455.1	59.6	6.5
Pseudomonas sp.	MIACH	GCA_001269925.1	60.7	6.8
Pseudomonas sp.	MIS38	GCA_019891575.1	60.3	6.2

Pseudomonas sp.	MMS21 TM103	GCA_022132145.1	62.3	4.9
Pseudomonas sp.	MONT-RG-20F-20-E-7-02	GCA_022662395.1	60.4	6.5
Pseudomonas sp.	MONT-RG-20F-R14-05	GCA_022662435.1	60.4	6.4
Pseudomonas sp.	MPBC4-3	GCA_002882535.1	60.3	7.2
Pseudomonas sp.	MPR-ANB1	GCA_002883355.1	60.3	7.4
Pseudomonas sp.	MPR-R2A6	GCA_017349375.1	60.4	7.4
Pseudomonas sp.	MPR-R2A7	GCA_002882515.1	60.3	7.5
Pseudomonas sp.	MPR-R5A	GCA_002883325.1	59.9	7.3
Pseudomonas sp.	MPR-R5B	GCA_002884045.1	60.3	7.2
Pseudomonas sp.	MSSRFD41	GCA_014230245.1	63.5	6.9
Pseudomonas sp.	MWU 12-2029	GCA_023242425.1	60.7	6.3
Pseudomonas sp.	MWU 12-3088	GCA_023242405.1	60.7	6.3
Pseudomonas sp.	MWU 13-2517	GCA_023242545.1	60.2	6.1
Pseudomonas sp.	MWU 13-2862	GCA_023242495.1	61.3	7.2
Pseudomonas sp.	MWU12-2020	GCA_023100605.1	60.3	6.3
Pseudomonas sp.	MWU12-2037	GCA_023242345.1	61.3	7.2
Pseudomonas sp.	MWU12-2311	GCA_024435475.1	61.2	7.3
Pseudomonas sp.	MWU12-2319	GCA_023242525.1	61.2	7.3
Pseudomonas sp.	MWU12-2323	GCA_009295765.1	61.0	7.9
Pseudomonas sp.	MWU12-2345	GCA_023242385.1	61.2	7.4
Pseudomonas sp.	MWU12-3091	GCA_023242465.1	60.7	6.3
Pseudomonas sp.	MWU12-3103b	GCA_023100615.1	60.2	6.3
Pseudomonas sp.	MWU13-2105	GCA_023242315.1	61.5	7.2
Pseudomonas sp.	MWU13-2924	GCA_023277215.1	61.3	7.2
Pseudomonas sp.	MWU13-3659	GCA_023757525.1	63.9	6.3
Pseudomonas sp.	MWU16-30316	GCA_020515645.1	59.2	6.0
Pseudomonas sp.	MWU16-30317	GCA_020515695.1	61.4	6.6
Pseudomonas sp.	MWU16-30322	GCA_020515535.1	60.4	6.2
Pseudomonas sp.	MWU16-30323	GCA_020515595.1	60.9	6.7
Pseudomonas sp.	MWU318	GCA_016756945.1	60.3	6.0
Pseudomonas sp.	MWU347	GCA_017947305.1	60.3	6.1
Pseudomonas sp.	MWU349	GCA_017947325.1	63.3	6.7
Pseudomonas sp.	MWU354	GCA_017947285.1	60.3	6.1
Pseudomonas sp.	MYb187	GCA_002979185.1	61.5	5.7
Pseudomonas sp.	NBRC 111117	GCA_001320045.1	64.5	5.5
Pseudomonas sp.	NBRC 111118	GCA_001320085.1	61.0	6.4
Pseudomonas sp.	NBRC 111120	GCA_001753895.1	62.4	5.5
Pseudomonas sp.	NBRC 111126	GCA_001753935.1	62.3	5.6
Pseudomonas sp.	NBRC 111128	GCA_001320385.1	62.2	5.7
Pseudomonas sp.	NBRC 111129	GCA_001320435.1	62.1	5.7
Pseudomonas sp.	NBRC 111138	GCA_001320835.1	60.0	6.1
Pseudomonas sp.	NBRC 111140	GCA_001320885.1	62.1	6.3
Pseudomonas sp.	NBRC 111144	GCA_001321005.1	62.4	5.4
Pseudomonas sp.	NFACC19-2	GCA_900119125.1	62.8	5.1
Pseudomonas sp.	NFIX51	GCA_900177395.1	63.1	6.5
Pseudomonas sp.	NFPP28	GCA_900115535.1	61.0	6.4

Pseudomonas sp.	NKUCC02_KPG	GCA_019345465.1	58.2	5.2
Pseudomonas sp.	NMI542_15	GCA_021283425.1	61.7	6.6
Pseudomonas sp.	NMI1173_11	GCA_021283105.1	61.7	6.5
Pseudomonas sp.	NMI4491_12	GCA_021282925.1	61.6	6.6
Pseudomonas sp.	OTU750018	GCA_011636575.1	59.9	4.4
Pseudomonas sp.	OV546	GCA_900116845.1	60.5	6.8
Pseudomonas sp.	P1.8	GCA_001269805.1	58.8	6.4
Pseudomonas sp.	P1.31	GCA_001269815.1	59.0	6.3
Pseudomonas sp.	p21	GCA_001642705.1	63.2	5.5
Pseudomonas sp.	p50	GCA_015351605.1	58.5	6.9
Pseudomonas sp.	P97.38	GCA_001269745.1	62.4	6.1
Pseudomonas sp.	P129	GCA_020312195.1	59.2	6.0
Pseudomonas sp.	P135	GCA_020312125.1	59.3	6.1
Pseudomonas sp.	P818	GCA_000418555.1	63.4	5.1
Pseudomonas sp.	P867	GCA_019891455.1	59.7	6.2
Pseudomonas sp.	PA-1-6G	GCA_021609515.1	60.1	6.0
Pseudomonas sp.	PA-3-6E	GCA_021607165.1	59.6	6.6
Pseudomonas sp.	PA-5-4A	GCA_021606205.1	60.0	6.7
Pseudomonas sp.	PA-5-4F	GCA_021606235.1	60.0	6.7
Pseudomonas sp.	PA-6-4F	GCA_021605745.1	60.0	6.4
Pseudomonas sp.	PAMC 26793	GCA_000313235.1	60.6	6.8
Pseudomonas sp.	PAMC 26818	GCA_019456775.1	60.0	6.1
Pseudomonas sp.	PAMC 29040	GCA_003994535.1	58.6	5.3
Pseudomonas sp.	PB100	GCA_009764255.1	60.5	5.9
Pseudomonas sp.	PB106	GCA_009764645.1	59.4	6.1
Pseudomonas sp.	PCH44	GCA_018304925.1	62.2	6.4
Pseudomonas sp.	PCH199	GCA_026192335.1	58.4	6.7
Pseudomonas sp.	PDM01	GCA_014852325.1	59.7	6.4
Pseudomonas sp.	PDM02	GCA_014852285.1	59.6	6.4
Pseudomonas sp.	PDM03	GCA_014852385.1	59.5	6.5
Pseudomonas sp.	PDM04	GCA_014851765.1	61.5	6.5
Pseudomonas sp.	PDM05	GCA_014851685.1	61.1	6.0
Pseudomonas sp.	PDM06	GCA_014851795.1	61.2	7.0
Pseudomonas sp.	PDM07	GCA_014852395.1	59.4	6.5
Pseudomonas sp.	PDM08	GCA_014852405.1	59.4	6.6
Pseudomonas sp.	PDM09	GCA_014852305.1	59.0	6.3
Pseudomonas sp.	PDM10	GCA_014852295.1	59.0	6.5
Pseudomonas sp.	PDM11	GCA_014851505.1	63.3	4.7
Pseudomonas sp.	PDM12	GCA_014852485.1	63.5	5.2
Pseudomonas sp.	PDM15	GCA_014851565.1	64.8	4.8
Pseudomonas sp.	PDM16	GCA_014851865.1	63.2	5.1
Pseudomonas sp.	PDM17	GCA_014851985.1	65.2	6.6
Pseudomonas sp.	PDM18	GCA_014852585.1	64.9	6.4
Pseudomonas sp.	PDM19	GCA_014852215.1	65.3	6.8
Pseudomonas sp.	PDM20	GCA_014852575.1	65.4	6.7
Pseudomonas sp.	PDM21	GCA_014852505.1	65.3	6.7

Pseudomonas sp.	PDM22	GCA_014851965.1	65.4	6.7
Pseudomonas sp.	PDM23	GCA_014852225.1	65.3	6.7
Pseudomonas sp.	PDM24	GCA_019218905.1	59.0	6.3
Pseudomonas sp.	PDM25	GCA_019219045.1	58.5	6.9
Pseudomonas sp.	PDM26	GCA_019219185.1	59.3	6.8
Pseudomonas sp.	PDM27	GCA_019219205.1	60.4	6.5
Pseudomonas sp.	PDM28	GCA_019219115.1	60.1	6.6
Pseudomonas sp.	PDM29	GCA_019219145.1	59.8	6.3
Pseudomonas sp.	PDM30	GCA_019218985.1	59.9	6.3
Pseudomonas sp.	PDM31	GCA_019218965.1	59.3	6.1
Pseudomonas sp.	PDM32	GCA_019219225.1	60.1	6.6
Pseudomonas sp.	PDM33	GCA_019219265.1	65.5	6.4
Pseudomonas sp.	PH1b	GCA_000633395.1	62.9	7.4
Pseudomonas sp.	PS01270	GCA_029959775.1	59.8	6.2
Pseudomonas sp.	PS01297	GCA_029959305.1	58.2	5.8
Pseudomonas sp.	PS01298	GCA_029959745.1	61.7	7.1
Pseudomonas sp.	PS01302	GCA_029959405.1	60.0	6.5
Pseudomonas sp.	PS01856	GCA_029959505.1	59.9	6.5
Pseudomonas sp.	PS02288	GCA_029959715.1	65.3	4.9
Pseudomonas sp.	PS02290	GCA_029960075.1	60.3	6.3
Pseudomonas sp.	PS02302	GCA_029959585.1	65.5	5.4
Pseudomonas sp.	PSB1	GCA_014596625.1	60.2	6.2
Pseudomonas sp.	PSB18	GCA_014596565.1	61.1	6.2
Pseudomonas sp.	Pse1	GCA_021341665.1	63.2	7.0
Pseudomonas sp.	F16(2018)	GCA_900291905.1	64.0	5.7
Pseudomonas sp.	FEN	GCA_904067045.1	61.1	6.8
Pseudomonas sp.	Kh7	GCA_900291035.1	61.9	5.8
Pseudomonas sp.	Kh13	GCA_900291015.1	62.8	5.5
Pseudomonas sp.	Kh14	GCA_900291005.1	61.9	5.8
Pseudomonas sp.	Pseudomonas_assembly	GCA_950101725.1	66.5	6.3
Pseudomonas sp.	PSNVIR1	GCA_921519095.1	62.3	5.7
Pseudomonas sp.	PvP007	GCA_017832075.1	59.1	6.0
Pseudomonas sp.	PvP009	GCA_017832995.1	59.2	5.9
Pseudomonas sp.	PvP025	GCA_017832155.1	61.1	5.7
Pseudomonas sp.	PvP027	GCA_017833005.1	59.1	5.9
Pseudomonas sp.	PvP028	GCA_017832255.1	59.0	6.0
Pseudomonas sp.	PvP088	GCA_017875115.1	61.7	6.8
Pseudomonas sp.	PvP089	GCA_017875165.1	61.7	6.8
Pseudomonas sp.	PvP100	GCA_017833055.1	59.1	6.0
Pseudomonas sp.	Q2-TVG4-2	GCA_013620795.1	60.6	4.5
Pseudomonas sp.	Q12-87	GCA_001269755.1	60.7	6.3
Pseudomonas sp.	QC2	GCA_002859085.1	60.7	6.5
Pseudomonas sp.	QS1027	GCA_002814235.1	61.4	7.5
Pseudomonas sp.	R3.Fl	GCA_023516765.1	67.2	7.5
Pseudomonas sp.	R22(2017)	GCA_002112605.1	60.6	6.4
Pseudomonas sp.	R31(2017)	GCA_002113405.1	60.3	6.3

Pseudomonas sp.	R32(2017)	GCA_002112485.1	60.6	6.4
Pseudomonas sp.	R39(2017)	GCA_002112385.1	60.3	6.3
Pseudomonas sp.	R45(2017)	GCA_002112305.1	60.7	6.3
Pseudomonas sp.	R47(2017)	GCA_002112315.1	60.6	6.4
Pseudomonas sp.	RC2C2	GCA_018390435.1	62.3	5.6
Pseudomonas sp.	RC4D1	GCA_018390395.1	62.1	6.8
Pseudomonas sp.	RG1	GCA_025910115.1	60.1	6.0
Pseudomonas sp.	RGM2987	GCA_022952855.1	61.3	6.2
Pseudomonas sp.	RIT 409	GCA_003052515.2	60.1	5.9
Pseudomonas sp.	RIT 411	GCA_003057735.2	66.2	5.3
Pseudomonas sp.	RIT 412	GCA_003057685.2	60.1	5.9
Pseudomonas sp.	RIT288	GCA_000631985.1	60.0	6.3
Pseudomonas sp.	RIT357	GCA_000632245.1	59.8	6.2
Pseudomonas sp.	RIT-PI-a	GCA_001187875.1	62.3	4.7
Pseudomonas sp.	RIT-PI-o	GCA_001297215.1	60.0	6.1
Pseudomonas sp.	RIT-PI-q	GCA_001297125.1	59.1	7.4
Pseudomonas sp.	RIT-PI-r	GCA_001297015.1	59.3	6.5
Pseudomonas sp.	RIT-PI-S	GCA_025642975.1	63.0	4.9
Pseudomonas sp.	RSAGOLDFG	GCA_901007645.1	60.2	6.2
Pseudomonas sp.	RW3S2	GCA_014268955.1	63.0	5.8
Pseudomonas sp.	S3E12	GCA_001702295.1	60.9	7.1
Pseudomonas sp.	S3E17	GCA_024168685.1	61.0	6.8
Pseudomonas sp.	S5(2021)	GCA_020075685.1	64.7	4.4
Pseudomonas sp.	S5D5	GCA_003013355.1	61.6	7.0
Pseudomonas sp.	S5F11	GCA_019641515.1	60.3	6.0
Pseudomonas sp.	S09F 262	GCA_002798475.1	60.8	6.5
Pseudomonas sp.	S9	GCA_000222125.2	56.5	4.8
Pseudomonas sp.	S10E 269	GCA_002798465.1	60.8	6.5
Pseudomonas sp.	S11A4	GCA_023098285.2	59.9	6.4
Pseudomonas sp.	S11P7	GCA_023195435.2	60.0	6.3
Pseudomonas sp.	S30	GCA_016518545.1	64.0	5.5
Pseudomonas sp.	S37	GCA_016658545.1	62.3	6.7
Pseudomonas sp.	SA3-5	GCA_027988105.1	62.5	5.2
Pseudomonas sp.	SbB1	GCA_013112475.1	62.0	6.0
Pseudomonas sp.	SCT	GCA_003864275.1	62.5	4.8
Pseudomonas sp.	SDM007_2	GCA_016924785.1	60.1	6.2
Pseudomonas sp.	sia0905	GCA_019219245.1	63.6	4.9
Pseudomonas sp.	SID14000	GCA_002165135.1	62.7	5.4
Pseudomonas sp.	SLBN-26	GCA_006715895.1	67.2	6.3
Pseudomonas sp.	SLFW	GCA_009905615.1	60.2	6.5
Pseudomonas sp.	SMT-1	GCA_003204195.1	61.6	6.1
Pseudomonas sp.	SR18	GCA_023701505.1	60.5	6.0
Pseudomonas sp.	SST3	GCA_003325755.2	59.9	5.6
Pseudomonas sp.	SWI36_1	GCA_004153505.1	62.0	5.8
Pseudomonas sp.	SWI36_2	GCA_004153435.1	62.0	5.8
Pseudomonas sp.	SWRI 103	GCA_012844475.1	60.8	6.0

Pseudomonas sp.	SWRI18	GCA_014268345.1	60.9	6.0
Pseudomonas sp.	SWRI22	GCA_014268395.2	60.1	5.9
Pseudomonas sp.	SWRI51	GCA_014268825.1	62.5	5.3
Pseudomonas sp.	SWRI59	GCA_014269115.1	62.6	5.9
Pseudomonas sp.	SWRI67	GCA_014269235.1	62.3	6.2
Pseudomonas sp.	SWRI68	GCA_014269165.1	62.6	5.9
Pseudomonas sp.	SWRI92	GCA_014268685.1	60.7	5.9
Pseudomonas sp.	SWRI111	GCA_014268555.1	60.0	6.1
Pseudomonas sp.	SWRI144	GCA_014268285.2	59.9	6.2
Pseudomonas sp.	T1.Ur	GCA_023516725.1	61.1	6.4
Pseudomonas sp.	T34	GCA_017815145.1	62.0	6.0
Pseudomonas sp.	TAA207	GCA_001529285.1	57.3	4.9
Pseudomonas sp.	TAD18	GCA_001529305.1	57.2	4.9
Pseudomonas sp.	TH03	GCA_016651375.1	58.9	6.3
Pseudomonas sp.	TH04	GCA_016651355.1	60.2	6.3
Pseudomonas sp.	TH06	GCA_016651305.1	59.2	6.6
Pseudomonas sp.	TH07	GCA_016651285.1	62.5	7.3
Pseudomonas sp.	TH08	GCA_016651275.1	59.1	6.6
Pseudomonas sp.	TH09	GCA_016651205.1	59.1	6.3
Pseudomonas sp.	TH15	GCA_016651105.1	59.2	6.3
Pseudomonas sp.	TH20	GCA_016651045.1	59.1	6.3
Pseudomonas sp.	TH21	GCA_016651025.1	61.7	6.8
Pseudomonas sp.	TH32	GCA_016650855.1	61.1	7.2
Pseudomonas sp.	TH35	GCA_016650765.1	59.1	6.3
Pseudomonas sp.	TH37	GCA_016650735.1	59.1	6.3
Pseudomonas sp.	TH38	GCA_016650745.1	59.1	6.3
Pseudomonas sp.	TH39(2020)	GCA_016650715.1	58.8	7.2
Pseudomonas sp.	TH40	GCA_016650725.1	59.1	6.3
Pseudomonas sp.	TH41	GCA_016650695.1	58.7	6.8
Pseudomonas sp.	TH43	GCA_016650635.1	59.0	6.5
Pseudomonas sp.	TH49	GCA_016650625.1	60.0	6.6
Pseudomonas sp.	TH71	GCA_016650535.1	59.1	6.3
Pseudomonas sp.	TKO14	GCA_003225705.1	63.1	6.9
Pseudomonas sp.	TKO26	GCA_003225665.1	63.1	6.9
Pseudomonas sp.	TKO29	GCA_003225735.1	63.1	6.9
Pseudomonas sp.	TKO30	GCA_003225695.1	63.1	6.9
Pseudomonas sp.	TMW22080	GCA_022496745.1	58.3	5.0
Pseudomonas sp.	TMW22090	GCA_022496665.1	59.9	6.2
Pseudomonas sp.	TMW22091	GCA_022496705.1	56.5	5.0
Pseudomonas sp.	TREG-RG-20F-10-E-5-01	GCA_022662415.1	60.5	6.5
Pseudomonas sp.	TREG-RG-20F-10-E-6-01	GCA_022662455.1	60.5	6.5
Pseudomonas sp.	TTU2014-066ASC	GCA_001446915.1	63.1	4.3
Pseudomonas sp.	TTU2014-080ASC	GCA_001446935.1	56.7	4.5
Pseudomonas sp.	TTU2014-096BSC	GCA_001446945.1	63.2	4.2
Pseudomonas sp.	UC 17F4	GCA_900101695.1	62.8	5.9
Pseudomonas sp.	UMC65	GCA_014170415.1	63.3	7.2

Pseudomonas sp.	UME65	GCA_014170225.1	63.3	7.1
Pseudomonas sp.	UME83	GCA_014170285.1	67.3	7.0
Pseudomonas sp.	USTB-Z	GCA_019691255.1	61.7	5.9
Pseudomonas sp.	UYIF39	GCA_029963765.1	58.7	6.6
Pseudomonas sp.	V88_4	GCA_029967985.1	60.5	6.3
Pseudomonas sp.	V104_10	GCA_029967945.1	62.3	5.6
Pseudomonas sp.	v388	GCA_003935425.1	61.0	5.5
Pseudomonas sp.	VA159-1	GCA_024127465.1	64.9	5.4
Pseudomonas sp.	VA159-2	GCA_024126635.1	64.9	5.4
Pseudomonas sp.	VB3	GCA_027864745.1	60.5	6.1
Pseudomonas sp.	VE 196-7	GCA_025599315.1	60.3	6.2
Pseudomonas sp.	VE 267-6A	GCA_024126555.1	64.4	5.6
Pseudomonas sp.	VS38	GCA_018587945.1	61.1	6.8
Pseudomonas sp.	VS40	GCA_018587885.1	60.8	6.5
Pseudomonas sp.	VS59	GCA_018587895.1	60.8	6.5
Pseudomonas sp.	WAC2	GCA_030403525.1	55.4	5.0
Pseudomonas sp.	WCS365	GCA_002796855.1	61.0	6.6
Pseudomonas sp.	WN033	GCA_002287035.1	59.9	4.2
Pseudomonas sp.	WS 5010	GCA_012985565.1	61.0	6.2
Pseudomonas sp.	WS 5011	GCA_012985775.1	59.5	4.6
Pseudomonas sp.	WS 5013	GCA_012985845.1	65.5	4.5
Pseudomonas sp.	WS 5019	GCA_012985785.1	62.7	5.2
Pseudomonas sp.	WS 5021	GCA_012985535.1	60.7	6.3
Pseudomonas sp.	WS 5027	GCA_012985695.1	60.1	6.2
Pseudomonas sp.	WS 5051	GCA_012985715.1	58.1	5.1
Pseudomonas sp.	WS 5059	GCA_012985525.1	61.0	6.2
Pseudomonas sp.	WS 5078	GCA_012985745.1	57.6	4.7
Pseudomonas sp.	WS 5079	GCA_012985465.1	60.9	6.3
Pseudomonas sp.	WS 5086	GCA_012985665.1	60.1	6.1
Pseudomonas sp.	WS 5111	GCA_012985485.1	61.0	6.3
Pseudomonas sp.	WS 5146	GCA_012985495.1	60.1	6.5
Pseudomonas sp.	WS 5354	GCA_012985675.1	57.6	4.7
Pseudomonas sp.	WS 5406	GCA_012985375.1	60.0	6.4
Pseudomonas sp.	WS 5410	GCA_012985645.1	60.6	6.3
Pseudomonas sp.	WS 5411	GCA_012985595.1	60.8	5.9
Pseudomonas sp.	WS 5412	GCA_012985615.1	60.5	6.2
Pseudomonas sp.	WS 5413	GCA_012985575.1	60.4	6.4
Pseudomonas sp.	WS 5503	GCA_012985385.1	59.4	6.7
Pseudomonas sp.	WS 5532	GCA_012985445.1	59.6	7.4
Pseudomonas sp.	Y24-6	GCA_020295325.1	59.7	6.2
Pseudomonas sp.	YY-1	GCA_002843095.1	62.2	5.0
Pseudomonas sp.	ZM23	GCA_024259745.1	64.0	6.2
Pseudomonas sp.	ZM24	GCA_024259765.1	63.6	6.5
Pseudomonas sp.	ZM25	GCA_024259725.1	63.5	6.5
Pseudomonas sp.	0qhubNDpsD_bin.31.MAG	GCA_946223245.1	60.3	5.8
Pseudomonas sp.	07-Jan	GCA_000742775.1	62.0	4.9

Pseudomonas sp.	1D4	GCA_001728925.1	66.3	5.8
Pseudomonas sp.	2VD	GCA_005503585.1	66.3	6.4
Pseudomonas sp.	3PA37B6	GCA_005503545.1	65.7	7.1
Pseudomonas sp.	5Ae-yellow	GCA_014077575.1	56.2	4.2
Pseudomonas sp.	5P_5.1_Bac1	GCA_025466015.1	63.2	6.5
Pseudomonas sp.	06C 126	GCA_001855215.1	60.6	6.0
Pseudomonas sp.	6D_7.1_Bac1	GCA_025465945.1	59.0	6.0
Pseudomonas sp.	7P_10.2_Bac1	GCA_025465935.1	57.7	5.4
Pseudomonas sp.	10-1B	GCA_000935045.1	62.4	6.3
Pseudomonas sp.	13B_2.1_Bac1	GCA_025466155.1	61.6	6.8
Pseudomonas sp.	13B_3.2_Bac1	GCA_025466205.1	60.4	6.2
Pseudomonas sp.	14P_5.3_Bac1	GCA_025466135.1	60.6	6.3
Pseudomonas sp.	14P_8.1_Bac3	GCA_025466085.1	60.5	6.5
Pseudomonas sp.	20P_3.2_Bac4	GCA_025466075.1	63.3	6.5
Pseudomonas sp.	20P_3.2_Bac5	GCA_025466035.1	63.3	6.5
Pseudomonas sp.	21C1	GCA_001728865.1	62.0	5.3
Pseudomonas sp.	21LCFQ010	GCA_024129905.1	60.5	6.3
Pseudomonas sp.	91RF	GCA_003576265.1	60.5	6.4
Pseudomonas sp.	313	GCA_000316965.1	65.3	5.2
Pseudomonas sp.	18047_Pseudomonas3_ Ancestor	GCA_902706265.1	59.4	6.4
Pseudomonas sp.	18058_Pseudomonas2_ Ancestor	GCA_902706155.1	58.9	5.8
Pseudomonas sp.	18091276	GCA_024718425.1	62.4	6.9
Pseudomonas sp.	A(2018)	GCA_005502595.1	64.3	4.4
Pseudomonas sp.	A25	GCA_002001065.1	60.9	4.6
Pseudomonas sp.	A46	GCA_002196875.1	64.7	6.4
Pseudomonas sp.	A192_concoct.bin.7	GCA_019459105.1	63.7	5.8
Pseudomonas sp.	AA27	GCA_021462485.1	64.8	6.5
Pseudomonas sp.	A-B-19	GCA_019947515.1	59.5	7.1
Pseudomonas sp.	ABAC61	GCA_001467175.1	63.3	6.3
Pseudomonas sp.	ACN8	GCA_002303925.1	59.8	6.7
Pseudomonas sp.	AF1	GCA_016820215.1	66.0	5.9
Pseudomonas sp.	AF32	GCA_022342325.1	61.1	5.2
Pseudomonas sp.	AFG_SD02_1510_Pfu_092	GCA_003331055.1	63.8	6.7
Pseudomonas sp.	AFW1	GCA_016820235.1	66.0	7.3
Pseudomonas sp.	Ag1	GCA_000278565.1	60.5	5.6
Pseudomonas sp.	ANIsp_001	GCA_021768505.1	63.3	6.1
Pseudomonas sp.	ANT_H14	GCA_008369285.1	58.6	6.2
Pseudomonas sp.	ANT_J28	GCA_008370005.1	58.9	7.4
Pseudomonas sp.	AP19	GCA_001728855.1	59.4	7.0
Pseudomonas sp.	A-R-19	GCA_019956575.1	59.9	6.3
Pseudomonas sp.	A-R-26	GCA_019947565.1	59.4	6.8
Pseudomonas sp.	A-RE-15	GCA_019955925.1	60.9	6.2
Pseudomonas sp.	A-RE-19	GCA_019956555.1	59.0	6.8
Pseudomonas sp.	A-RE-22	GCA_019956035.1	60.9	6.9
Pseudomonas sp.	A-RE-23	GCA_019955975.1	60.7	5.9
Pseudomonas sp.	A-RE-26	GCA_019947495.1	60.8	6.8

Pseudomonas sp.	A-RE-6	GCA_019955915.1	60.9	6.8
Pseudomonas sp.	A-RE-7	GCA_019947185.1	60.9	6.8
Pseudomonas sp.	A-RE-8	GCA_019955875.1	60.9	2.2
Pseudomonas sp.	AS08sgBPME_395	GCA_012841125.1	48.8	7.2
Pseudomonas sp.	ASV14	GCA_016429135.1	62.8	6.8
Pseudomonas sp.	AU8050	GCA_011290325.1	60.0	7.3
Pseudomonas sp.	AU11447	GCA_001672555.2	64.6	6.1
Pseudomonas sp.	AU12215	GCA_001675465.2	64.1	6.2
Pseudomonas sp.	B10	GCA_004153525.1	60.8	5.5
Pseudomonas sp.	BAV 2493	GCA_009765535.1	65.4	2.6
Pseudomonas sp.	BC.3.46	GCA_013823765.1	59.3	4.2
Pseudomonas sp.	BC.3.70	GCA_013823775.1	58.8	5.9
Pseudomonas sp.	BC115LW	GCA_009905775.1	61.0	5.4
Pseudomonas sp.	BDAL1	GCA_001723935.1	58.4	6.4
Pseudomonas sp.	BDPW	GCA_016820275.1	66.3	4.4
Pseudomonas sp.	BDu8dbSWIw_bin.47.MAG	GCA_943913815.1	62.1	6.0
Pseudomonas sp.	BF61	GCA_018904795.1	60.6	6.6
Pseudomonas sp.	BF-B-19	GCA_019956505.1	59.5	6.5
Pseudomonas sp.	BF-B-25	GCA_019955795.1	59.5	6.7
Pseudomonas sp.	BF-B-26	GCA_019956495.1	58.6	6.2
Pseudomonas sp.	BF-B-27	GCA_019955815.1	59.8	6.2
Pseudomonas sp.	BF-B-28	GCA_019955775.1	59.8	6.3
Pseudomonas sp.	BF-B-30	GCA_019956305.1	59.7	6.4
Pseudomonas sp.	BF-R-12	GCA_019955855.1	59.6	6.5
Pseudomonas sp.	BF-R-16	GCA_019946635.1	59.6	6.8
Pseudomonas sp.	BF-R-19	GCA_019956445.1	59.8	6.2
Pseudomonas sp.	BF-R-21	GCA_019947355.1	59.9	6.2
Pseudomonas sp.	BF-R-24	GCA_019947335.1	59.9	6.5
Pseudomonas sp.	BF-R-25	GCA_019956235.1	59.6	6.3
Pseudomonas sp.	BF-R-30	GCA_019956175.1	59.8	6.6
Pseudomonas sp.	BF-RE-03	GCA_019947155.1	59.5	6.4
Pseudomonas sp.	BF-RE-04	GCA_019955825.1	59.6	6.4
Pseudomonas sp.	BF-RE-09	GCA_019946575.1	59.6	6.2
Pseudomonas sp.	BF-RE-19	GCA_019956435.1	59.9	6.7
Pseudomonas sp.	BF-RE-21	GCA_019947305.1	59.5	6.5
Pseudomonas sp.	BF-RE-22	GCA_019956155.1	59.6	6.6
Pseudomonas sp.	BF-RE-24	GCA_019956095.1	60.9	6.4
Pseudomonas sp.	BF-RE-25	GCA_019956105.1	59.6	6.4
Pseudomonas sp.	BF-RE-26	GCA_019956475.1	59.9	6.6
Pseudomonas sp.	BF-RE-28	GCA_019956115.1	59.5	6.4
Pseudomonas sp.	BF-RE-29	GCA_019947295.1	59.8	8.0
Pseudomonas sp.	BGI-2	GCA_004310295.1	58.9	5.6
Pseudomonas sp.	BGr12	GCA_030272895.1	63.6	5.3
Pseudomonas sp.	Bi70	GCA_918698015.1	63.0	7.3
Pseudomonas sp.	BIGb0450	GCA_024807645.1	60.9	7.3
Pseudomonas sp.	BIGb0558	GCA_024807515.1	60.9	6.7
Pseudomonas sp.	BIS	GCA_016820195.1	66.0	6.7

Pseudomonas sp.	BIS1	GCA_016820175.1	66.1	6.3
Pseudomonas sp.	BP01	GCA_022760795.1	62.2	3.1
Pseudomonas sp.	BRH_c35	GCA_000961835.1	61.1	6.2
Pseudomonas sp.	BSL-kmcMAG004	GCA_016785205.1	60.6	6.1
Pseudomonas sp.	BTN1	GCA_001907715.1	60.1	6.0
Pseudomonas sp.	BW16M2	GCA_014268865.1	64.8	6.7
Pseudomonas sp.	C32_concoct.bin.14	GCA_019459245.1	62.4	4.0
Pseudomonas sp.	C42_metabat.bin.8	GCA_019459405.1	64.3	6.2
Pseudomonas sp.	C5pp	GCA_000814065.1	62.3	5.7
Pseudomonas sp.	CAH-1	GCA_009668315.1	62.1	5.8
Pseudomonas sp.	CES	GCA_012979115.1	62.6	5.9
Pseudomonas sp.	CFII68	GCA_000416195.1	60.8	6.2
Pseudomonas sp.	CH235	GCA_004368175.1	60.8	6.7
Pseudomonas sp.	CHM02	GCA_000612585.1	60.9	6.1
Pseudomonas sp.	D(2018)	GCA_005502555.1	63.5	6.3
Pseudomonas sp.	DCB_AW	GCA_026240895.1	62.3	6.3
Pseudomonas sp.	DCB_BZ	GCA_026241035.1	61.6	6.3
Pseudomonas sp.	DCB_CB	GCA_026241055.1	61.6	6.0
Pseudomonas sp.	DCB_E	GCA_026241065.1	61.8	4.0
Pseudomonas sp.	DF_1_3.23	GCA_019105025.1	64.9	5.7
Pseudomonas sp.	DP16D-E2	GCA_002884365.1	64.2	6.6
Pseudomonas sp.	DP16D-L5	GCA_002881915.1	60.8	7.0
Pseudomonas sp.	DP16D-R1	GCA_002901625.1	59.2	6.9
Pseudomonas sp.	DP16D-R1	GCA_017351935.1	59.3	4.7
Pseudomonas sp.	DS1.001	GCA_013823455.1	65.0	5.8
Pseudomonas sp.	DVZ24	GCA_028728275.1	64.8	5.8
Pseudomonas sp.	DVZ6	GCA_024259445.1	64.8	5.7
Pseudomonas sp.	efr-133-TYG-23	GCA_030209215.1	60.7	5.1
Pseudomonas sp.	EGD-AK9	GCA_000465935.2	65.6	5.8
Pseudomonas sp.	ENNP23	GCA_001728945.1	66.3	6.1
Pseudomonas sp.	ES3-33	GCA_000952175.1	59.7	4.4
Pseudomonas sp.	FEMGT703P	GCA_002786345.1	59.9	5.3
Pseudomonas sp.	FFUP_PS_473	GCA_002858665.1	59.8	7.1
Pseudomonas sp.	FH1	GCA_000510895.2	60.1	5.3
Pseudomonas sp.	Fl4BN2	GCA_009908305.1	59.0	6.0
Pseudomonas sp.	fls2-241-TYG-175	GCA_030209155.1	59.4	5.4
Pseudomonas sp.	FSL R10-0056	GCA_009600105.1	58.0	6.1
Pseudomonas sp.	FSL R10-0071	GCA_009600185.1	57.7	6.2
Pseudomonas sp.	FSL R10-0765	GCA_009600045.1	57.9	5.7
Pseudomonas sp.	FSL R10-1350	GCA_009600525.1	58.1	5.9
Pseudomonas sp.	FSL R10-2172	GCA_009600365.1	58.1	5.8
Pseudomonas sp.	FSL R10-2189	GCA_009600325.1	58.2	6.1
Pseudomonas sp.	FSL R10-2245	GCA_009600385.1	58.0	5.1
Pseudomonas sp.	FSL R10-2964	GCA_009600035.1	58.4	6.3
Pseudomonas sp.	FSL W5-0203	GCA_001896155.1	59.7	7.1
Pseudomonas sp.	FSL W5-0299	GCA_002005125.1	59.2	7.1
Pseudomonas sp.	FSL W5-0299	GCA_005930655.1	59.2	7.1

Pseudomonas sp.	FW126-L8	GCA_002883425.1	62.7	7.0
Pseudomonas sp.	FW215-E1	GCA_002882485.1	60.3	6.5
Pseudomonas sp.	FW215-E1	GCA_017351875.1	60.3	6.5
Pseudomonas sp.	FW215-L1	GCA_002882435.1	60.3	6.5
Pseudomonas sp.	FW215-R2	GCA_017351855.1	60.3	6.5
Pseudomonas sp.	FW215-R3	GCA_017351835.1	59.4	6.5
Pseudomonas sp.	FW215-R3	GCA_002883895.1	59.4	6.3
Pseudomonas sp.	FW215-R4	GCA_002883275.1	60.3	6.3
Pseudomonas sp.	FW215-R4	GCA_017351795.1	60.3	6.5
Pseudomonas sp.	FW215-T2	GCA_017351805.1	59.4	6.3
Pseudomonas sp.	FW215-T2	GCA_002883935.1	59.4	6.4
Pseudomonas sp.	FW300-E2	GCA_002884205.1	60.3	6.5
Pseudomonas sp.	FW300-N1A1	GCA_002901565.1	60.5	6.5
Pseudomonas sp.	FW300-N1A5	GCA_002883815.1	58.8	6.4
Pseudomonas sp.	FW303-C2	GCA_002883505.1	59.4	6.4
Pseudomonas sp.	FW305-113	GCA_017351565.1	64.2	5.9
Pseudomonas sp.	FW305-117	GCA_017351575.1	60.3	5.6
Pseudomonas sp.	FW305-121	GCA_017351555.1	59.4	6.4
Pseudomonas sp.	FW305-122	GCA_017351535.1	59.0	6.4
Pseudomonas sp.	FW305-122	GCA_002881725.1	59.0	6.2
Pseudomonas sp.	FW305-124	GCA_002883585.1	60.3	5.6
Pseudomonas sp.	FW305-124	GCA_017351515.1	60.3	5.8
Pseudomonas sp.	FW305-127	GCA_002884395.1	64.2	5.8
Pseudomonas sp.	FW305-127	GCA_017351465.1	64.2	5.6
Pseudomonas sp.	FW305-130	GCA_002884175.1	62.0	6.4
Pseudomonas sp.	FW305-131	GCA_002884305.1	59.4	6.2
Pseudomonas sp.	FW305-136	GCA_017351475.1	60.3	6.2
Pseudomonas sp.	FW305-17	GCA_002884415.1	64.2	5.9
Pseudomonas sp.	FW305-17	GCA_017351455.1	64.2	5.6
Pseudomonas sp.	FW305-20	GCA_017351435.1	59.0	5.8
Pseudomonas sp.	FW305-20	GCA_002881715.1	59.0	6.2
Pseudomonas sp.	FW305-25	GCA_017351415.1	62.7	5.7
Pseudomonas sp.	FW305-25	GCA_002883455.1	62.7	7.0
Pseudomonas sp.	FW305-3-2-15-A-LB2	GCA_017351345.1	60.8	5.6
Pseudomonas sp.	FW305-3-2-15-A-LB2	GCA_002881955.1	60.8	6.6
Pseudomonas sp.	FW305-3-2-15-A-R2A1	GCA_002883235.1	59.2	7.1
Pseudomonas sp.	FW305-3-2-15-A-R2A1	GCA_002883055.1	59.1	6.4
Pseudomonas sp.	FW305-3-2-15-A-R2A1	GCA_017351335.1	59.1	7.2
Pseudomonas sp.	FW305-3-2-15-A-R2A2	GCA_017351315.1	60.8	5.8
Pseudomonas sp.	FW305-3-2-15-A-TSA1	GCA_017351375.1	60.8	5.7
Pseudomonas sp.	FW305-3-2-15-A-TSA3	GCA_017351295.1	60.8	5.6
Pseudomonas sp.	FW305-3-2-15-C-LB1	GCA_002882055.1	60.8	6.2
Pseudomonas sp.	FW305-3-2-15-C-LB1	GCA_017351215.1	60.8	6.6
Pseudomonas sp.	FW305-3-2-15-C-LB3	GCA_017351245.1	60.8	6.2
Pseudomonas sp.	FW305-3-2-15-C-LB3	GCA_002881975.1	60.8	6.6
Pseudomonas sp.	FW305-3-2-15-C-R2A1	GCA_017351255.1	60.8	6.9
Pseudomonas sp.	FW305-3-2-15-C-R2A1	GCA_002882925.1	60.8	6.6

Pseudomonas sp.	FW305-3-2-15-C-TSA2	GCA_017351225.1	60.8	7.0
Pseudomonas sp.	FW305-3-2-15-C-TSA3	GCA_017351135.1	60.9	6.6
Pseudomonas sp.	FW305-3-2-15-C-TSA3	GCA_002881995.1	60.9	6.5
Pseudomonas sp.	FW305-3-2-15-E-TSA2	GCA_002901605.1	60.2	6.6
Pseudomonas sp.	FW305-3-2-15-E-TSA2	GCA_017351195.1	60.3	6.2
Pseudomonas sp.	FW305-3-2-15-E-TSA4	GCA_002901465.1	60.3	7.1
Pseudomonas sp.	FW305-33	GCA_017351125.1	59.0	7.2
Pseudomonas sp.	FW305-33	GCA_002882695.1	59.0	6.3
Pseudomonas sp.	FW305-34	GCA_017351115.1	60.3	7.2
Pseudomonas sp.	FW305-42	GCA_002883975.1	64.2	6.6
Pseudomonas sp.	FW305-42	GCA_017351095.1	64.2	6.5
Pseudomonas sp.	FW305-47B	GCA_017351075.1	59.0	6.6
Pseudomonas sp.	FW305-47B	GCA_002881635.1	59.0	6.2
Pseudomonas sp.	FW305-53	GCA_017351055.1	59.4	6.6
Pseudomonas sp.	FW305-53	GCA_002883475.1	59.4	6.4
Pseudomonas sp.	FW305-55	GCA_017351035.1	60.8	6.6
Pseudomonas sp.	FW305-56	GCA_017350995.1	59.4	6.6
Pseudomonas sp.	FW305-60	GCA_017351015.1	59.0	6.6
Pseudomonas sp.	FW305-60	GCA_002882675.1	59.0	6.2
Pseudomonas sp.	FW305-62	GCA_002883495.1	59.4	6.6
Pseudomonas sp.	FW305-62	GCA_017350955.1	59.4	6.4
Pseudomonas sp.	FW305-67	GCA_002884115.1	59.4	6.6
Pseudomonas sp.	FW305-67	GCA_017350935.1	59.4	6.4
Pseudomonas sp.	FW305-70	GCA_017350975.1	60.2	6.6
Pseudomonas sp.	FW305-70	GCA_002883915.1	60.2	6.3
Pseudomonas sp.	FW305-76	GCA_017350875.1	62.7	6.9
Pseudomonas sp.	FW305-76	GCA_002884075.1	62.7	7.0
Pseudomonas sp.	FW305-90	GCA_017350905.1	60.3	6.5
Pseudomonas sp.	FW305-96	GCA_017350895.1	64.2	6.2
Pseudomonas sp.	FW305-BF15	GCA_002883945.1	60.7	6.2
Pseudomonas sp.	FW305-BF6	GCA_002884325.1	59.6	6.2
Pseudomonas sp.	FW305-BF8	GCA_002883215.1	59.3	6.2
Pseudomonas sp.	FW305-E2	GCA_017350855.1	61.8	6.3
Pseudomonas sp.	FW305-E2	GCA_002901725.1	61.8	6.1
Pseudomonas sp.	FW306-02-F02-AA	GCA_017350835.1	58.8	5.8
Pseudomonas sp.	FW306-02-F02-AA	GCA_002883635.1	58.8	6.4
Pseudomonas sp.	FW306-02-F02-AB	GCA_002883655.1	58.8	6.4
Pseudomonas sp.	FW306-02-F02-AB	GCA_017350815.1	58.8	6.3
Pseudomonas sp.	FW306-02-F04-AA	GCA_002884235.1	58.8	6.4
Pseudomonas sp.	FW306-02-F04-AA	GCA_017350785.1	58.8	6.3
Pseudomonas sp.	FW306-02-F04-BA	GCA_002883695.1	58.8	6.4
Pseudomonas sp.	FW306-02-F04-BA	GCA_017350745.1	58.8	6.3
Pseudomonas sp.	FW306-02-F08-AA	GCA_002883595.1	58.9	6.4
Pseudomonas sp.	FW306-02-F08-AA	GCA_017350755.1	58.8	6.3
Pseudomonas sp.	FW306-02-H05-AA	GCA_017350735.1	58.8	6.4
Pseudomonas sp.	FW306-02-H05-AA	GCA_002883735.1	58.8	6.4
Pseudomonas sp.	FW306-02-H05-AB	GCA_002883845.1	58.8	6.6

Pseudomonas sp.	FW306-02-H05-BA	GCA_002884255.1	58.8	6.4
Pseudomonas sp.	FW306-02-H05-BA	GCA_017350705.1	58.8	6.3
Pseudomonas sp.	FW306-02-H06B	GCA_002883775.1	58.8	6.4
Pseudomonas sp.	FW306-02-H06B	GCA_017350675.1	58.8	6.3
Pseudomonas sp.	FW306-02-H06C	GCA_002883675.1	58.9	6.4
Pseudomonas sp.	FW306-02-H06C	GCA_017350635.1	58.8	6.2
Pseudomonas sp.	FW306-07-L	GCA_002882175.1	59.3	6.4
Pseudomonas sp.	FW306-1C-G01A	GCA_002882345.1	59.3	6.4
Pseudomonas sp.	FW306-2-11AA	GCA_002883785.1	58.9	6.4
Pseudomonas sp.	FW306-2-11AB	GCA_002883745.1	58.8	6.4
Pseudomonas sp.	FW306-2-11AC	GCA_002883715.1	58.8	6.2
Pseudomonas sp.	FW306-2-11AC	GCA_017350555.1	58.8	6.3
Pseudomonas sp.	FW306-2-11AD	GCA_017350575.1	58.8	6.3
Pseudomonas sp.	FW306-2-11AD	GCA_002883835.1	58.9	6.5
Pseudomonas sp.	FW306-2-11BA	GCA_002884275.1	58.9	6.4
Pseudomonas sp.	FW306-2-11BA	GCA_017350595.1	58.8	6.3
Pseudomonas sp.	FW306-2-1A-C05A	GCA_002882095.1	59.3	6.9
Pseudomonas sp.	FW306-2-2C-A10BC	GCA_002884085.1	59.4	5.7
Pseudomonas sp.	FW306-2-2C-B10A	GCA_002882155.1	59.3	5.7
Pseudomonas sp.	FW306-2-2C-D06C	GCA_002883375.1	62.9	6.3
Pseudomonas sp.	FW507-12TSA	GCA_017350535.1	63.6	6.8
Pseudomonas sp.	FW507-12TSA	GCA_002901645.1	63.6	6.9
Pseudomonas sp.	G(2018)	GCA_005502415.1	59.0	6.9
Pseudomonas sp.	G5	GCA_000408945.1	59.3	6.0
Pseudomonas sp.	Gammapro4	GCA_002862505.1	57.1	6.1
Pseudomonas sp.	GBPI_506	GCA_024339145.1	59.7	6.2
Pseudomonas sp.	GC01	GCA_013002835.1	58.6	6.4
Pseudomonas sp.	GD03651	GCA_029845165.1	61.6	6.3
Pseudomonas sp.	GD03689	GCA_029844385.1	64.7	6.4
Pseudomonas sp.	GD03691	GCA_029844365.1	64.8	6.3
Pseudomonas sp.	GD03696	GCA_029844065.1	60.6	6.4
Pseudomonas sp.	GD03722	GCA_029843605.1	62.3	6.4
Pseudomonas sp.	GD03730	GCA_029843415.1	61.6	6.3
Pseudomonas sp.	GD03766	GCA_029842685.1	61.8	6.3
Pseudomonas sp.	GD03817	GCA_029841705.1	61.6	6.4
Pseudomonas sp.	GD03860	GCA_029840985.1	61.9	6.3
Pseudomonas sp.	GD03862	GCA_029840855.1	61.8	6.4
Pseudomonas sp.	GD03867	GCA_029840765.1	64.4	6.4
Pseudomonas sp.	GD03869	GCA_029840705.1	66.4	6.3
Pseudomonas sp.	GD03875	GCA_029840585.1	66.3	6.4
Pseudomonas sp.	GD03985	GCA_029838465.1	66.3	6.3
Pseudomonas sp.	GD04015	GCA_029837675.1	66.4	6.4
Pseudomonas sp.	GD04045	GCA_029837115.1	65.0	6.2
Pseudomonas sp.	GD04058	GCA_029836855.1	63.3	6.4
Pseudomonas sp.	GD04087	GCA_029836255.1	64.8	6.9
Pseudomonas sp.	GL93	GCA_003408955.1	60.0	6.9
Pseudomonas sp.	GL-B-12	GCA_019955895.1	59.6	6.4

Pseudomonas sp.	GL-B-16	GCA_019947245.1	59.4	6.4
Pseudomonas sp.	GL-B-19	GCA_019956415.1	58.5	6.4
Pseudomonas sp.	GL-B-26	GCA_019947405.1	59.0	6.3
Pseudomonas sp.	GL-R-19	GCA_019956375.1	59.5	6.3
Pseudomonas sp.	GL-RE-19	GCA_019956245.1	59.5	6.5
Pseudomonas sp.	GL-RE-20	GCA_019947235.1	59.6	6.3
Pseudomonas sp.	GL-RE-26	GCA_019956275.1	59.9	6.4
Pseudomonas sp.	GL-RE-29	GCA_019947275.1	59.0	6.9
Pseudomonas sp.	GM102	GCA_000282555.1	59.0	6.4
Pseudomonas sp.	GM16	GCA_000282155.1	59.2	6.9
Pseudomonas sp.	GM18	GCA_000282195.1	59.5	6.8
Pseudomonas sp.	GM25	GCA_000282255.1	60.9	6.9
Pseudomonas sp.	GM49	GCA_000282355.1	59.6	6.8
Pseudomonas sp.	GM50	GCA_000282375.1	59.0	6.6
Pseudomonas sp.	GM55	GCA_000282395.1	59.7	7.2
Pseudomonas sp.	GM67	GCA_000282435.1	59.6	5.0
Pseudomonas sp.	GM74	GCA_000282455.1	60.1	6.6
Pseudomonas sp.	GM79	GCA_000282495.1	58.8	4.7
Pseudomonas sp.	GM84	GCA_000282535.1	63.2	6.7
Pseudomonas sp.	GP01-A1	GCA_002882775.1	60.9	7.4
Pseudomonas sp.	GP01-A1	GCA_017350495.1	60.9	7.3
Pseudomonas sp.	GP01-A11	GCA_017350455.1	60.9	6.8
Pseudomonas sp.	GP01-A13	GCA_017350465.1	60.9	6.3
Pseudomonas sp.	GP01-A14	GCA_002881785.1	60.9	7.4
Pseudomonas sp.	GP01-A14	GCA_017350435.1	60.9	7.2
Pseudomonas sp.	GP01-A15	GCA_017350415.1	60.9	6.2
Pseudomonas sp.	GP01-A4	GCA_002881845.1	61.0	7.5
Pseudomonas sp.	GP01-A4	GCA_017350375.1	60.9	7.2
Pseudomonas sp.	GP01-A5	GCA_017350355.1	60.9	6.2
Pseudomonas sp.	GP01-A5	GCA_002882735.1	60.9	6.1
Pseudomonas sp.	GP01-A6	GCA_017350325.1	60.9	6.0
Pseudomonas sp.	GV_Bin_12	GCA_009360675.1	63.6	5.8
Pseudomonas sp.	GW101-1A09	GCA_002882985.1	59.3	5.4
Pseudomonas sp.	GW123-5C08	GCA_002882975.1	60.8	5.5
Pseudomonas sp.	GW123-5D08	GCA_002882955.1	60.8	5.5
Pseudomonas sp.	GW456-11-11-14-LB1	GCA_002883875.1	58.9	5.4
Pseudomonas sp.	GW456-11-11-14-LB2	GCA_002882415.1	59.2	4.8
Pseudomonas sp.	GW456-11-11-14-TSB2	GCA_017350245.1	59.2	6.2
Pseudomonas sp.	GW456-12-10-14-LB2	GCA_002884345.1	60.9	6.8
Pseudomonas sp.	GW456-12-10-14-TSB6	GCA_017350185.1	59.2	6.2
Pseudomonas sp.	GW456-12-10-14-TSB6	GCA_017350185.1	59.2	7.0
Pseudomonas sp.	GW456-12-1-14-LB2	GCA_002883095.1	59.4	6.7
Pseudomonas sp.	GW456-12-1-14-TSB1	GCA_017350215.1	59.1	7.0
Pseudomonas sp.	GW456-12-1-14-TSB1	GCA_002882295.1	59.1	7.2
Pseudomonas sp.	GW456-12-1-14-TSB6	GCA_017350165.1	60.6	6.3
Pseudomonas sp.	GW456-12-1-14-TSB6	GCA_002901495.1	60.6	6.3
Pseudomonas sp.	GW456-E6	GCA_017350155.1	59.2	6.9

Pseudomonas sp.	GW456-E6	GCA_002883035.1	59.2	7.0
Pseudomonas sp.	GW456-E7	GCA_002884295.1	59.2	6.5
Pseudomonas sp.	GW456-L12	GCA_002883415.1	62.7	7.0
Pseudomonas sp.	GW456-L12	GCA_017350135.1	62.7	7.0
Pseudomonas sp.	GW456-L14	GCA_017350085.1	62.7	6.8
Pseudomonas sp.	GW456-L14	GCA_002883395.1	62.8	7.0
Pseudomonas sp.	GW456-R21	GCA_002901545.1	58.9	6.4
Pseudomonas sp.	GW458-11-11-14-TSB1	GCA_017350035.1	59.4	6.4
Pseudomonas sp.	GW458-11-26-14-LB3	GCA_017350075.1	59.3	6.7
Pseudomonas sp.	GW458-11-26-14-LB4	GCA_017350015.1	60.7	6.6
Pseudomonas sp.	GW458-11-26-14-TSB3	GCA_017349975.1	59.3	6.3
Pseudomonas sp.	GW458-12-2-14-LB2	GCA_017349955.1	59.3	6.4
Pseudomonas sp.	GW458-12-2-14-TSB1	GCA_017349995.1	59.3	6.6
Pseudomonas sp.	GW458-12-9-14-LB1	GCA_017349915.1	59.3	6.7
Pseudomonas sp.	GW458-12-9-14-TSB2	GCA_017349935.1	59.3	6.5
Pseudomonas sp.	GW460-11-11-14-LB11	GCA_002882235.1	59.3	6.5
Pseudomonas sp.	GW460-11-11-14-LB11	GCA_017349895.1	59.4	6.7
Pseudomonas sp.	GW460-12-10-14-LB1	GCA_017349835.1	59.3	6.1
Pseudomonas sp.	GW460-12-10-14-TSB1	GCA_017349775.1	62.9	6.7
Pseudomonas sp.	GW460-12-1-14-LB3	GCA_002882395.1	59.4	6.7
Pseudomonas sp.	GW460-12-1-14-LB3	GCA_017349865.1	59.4	6.7
Pseudomonas sp.	GW460-13	GCA_002883155.1	59.5	7.4
Pseudomonas sp.	GW460-13	GCA_017349805.1	59.2	7.0
Pseudomonas sp.	GW460-4	GCA_002882015.1	60.8	7.4
Pseudomonas sp.	GW460-5	GCA_002884155.1	61.8	7.3
Pseudomonas sp.	GW460-7	GCA_002882195.1	59.2	7.1
Pseudomonas sp.	GW460-7	GCA_017349755.1	59.2	7.0
Pseudomonas sp.	GW460-8	GCA_002882315.1	59.2	7.1
Pseudomonas sp.	GW460-8	GCA_017349715.1	59.2	7.0
Pseudomonas sp.	GW460-C3	GCA_002883195.1	59.2	7.1
Pseudomonas sp.	GW460-C3	GCA_017349735.1	59.2	7.0
Pseudomonas sp.	GW460-C8	GCA_002882075.1	59.2	7.3
Pseudomonas sp.	GW460-E13	GCA_017349635.1	59.2	7.0
Pseudomonas sp.	GW460-E13	GCA_002882335.1	59.2	7.1
Pseudomonas sp.	GW460-LB5	GCA_017349655.1	61.8	7.5
Pseudomonas sp.	GW460-R15	GCA_002901575.1	59.0	7.3
Pseudomonas sp.	GW531-E2	GCA_002883995.1	64.2	7.4
Pseudomonas sp.	GW531-R1	GCA_002901675.1	60.5	5.7
Pseudomonas sp.	GW531-R1	GCA_017349665.1	60.5	5.6
Pseudomonas sp.	GW531-T4	GCA_002901665.1	63.1	6.7
Pseudomonas sp.	GW531-T4	GCA_017349615.1	63.1	6.7
Pseudomonas sp.	GW704-E2	GCA_017349595.1	61.0	6.9
Pseudomonas sp.	GW704-F2	GCA_002881895.1	60.9	6.7
Pseudomonas sp.	GW704-F2	GCA_017349515.1	60.9	6.6
Pseudomonas sp.	GW704-F3	GCA_017349545.1	61.0	6.6
Pseudomonas sp.	GW704-F3	GCA_002882855.1	60.9	6.7
Pseudomonas sp.	GW704-F5	GCA_017349525.1	61.0	6.6

Pseudomonas sp.	GW704-F5	GCA_002882895.1	60.9	6.6
Pseudomonas sp.	GXM4	GCA_008923895.1	60.0	7.1
Pseudomonas sp.	H2	GCA_000763225.1	62.6	7.0
Pseudomonas sp.	HD6421	GCA_025377045.1	61.6	6.2
Pseudomonas sp.	HD6422	GCA_025377065.1	61.9	7.1
Pseudomonas sp.	ICMP17674	GCA_000467045.1	63.4	7.0
Pseudomonas sp.	ID1025	GCA_028656935.1	61.3	6.7
Pseudomonas sp.	ID291	GCA_028657115.1	61.3	7.1
Pseudomonas sp.	ID609	GCA_028657075.1	61.3	7.2
Pseudomonas sp.	ID656	GCA_028656975.1	61.9	6.3
Pseudomonas sp.	ID681	GCA_028656955.1	61.9	6.3
Pseudomonas sp.	ITEM 17296	GCA_028895205.1	62.2	7.0
Pseudomonas sp.	JGI 0001012-A11	GCA_000484855.1	56.4	6.9
Pseudomonas sp.	K2	GCA_014889495.1	61.1	7.2
Pseudomonas sp.	K2I15	GCA_002204795.1	60.4	7.0
Pseudomonas sp.	K5	GCA_014889415.1	61.2	7.0
Pseudomonas sp.	K8	GCA_014889455.1	61.1	6.8
Pseudomonas sp.	L3_058_000G1_dasL3_058_000G1_concoct_33	GCA_018373475.1	62.4	7.0
Pseudomonas sp.	M30B71	GCA_018401165.1	62.6	6.2
Pseudomonas sp.	M47T1	GCA_000263855.1	62.5	6.4
Pseudomonas sp.	MAG-102	GCA_014359465.1	64.6	6.8
Pseudomonas sp.	Marseille-Q1929	GCA_017316265.1	61.0	6.3
Pseudomonas sp.	MCMED-G45	GCA_013911245.1	63.5	6.8
Pseudomonas sp.	MCMED-G46	GCA_013911255.1	61.9	6.8
Pseudomonas sp.	MCPNR19-03	GCA_022869205.1	60.5	6.8
Pseudomonas sp.	MD195_PC81_125	GCA_014060985.1	59.7	6.8
Pseudomonas sp.	MDMC17	GCA_003269375.2	62.7	6.8
Pseudomonas sp.	MEJ086	GCA_030209025.1	62.4	6.9
Pseudomonas sp.	MEJ108	GCA_030208985.1	59.3	6.7
Pseudomonas sp.	ME-P-057	GCA_030208975.1	60.2	6.8
Pseudomonas sp.	MF6394	GCA_002018845.1	59.5	6.7
Pseudomonas sp.	MF6396	GCA_002018915.1	63.9	6.7
Pseudomonas sp.	MF6751	GCA_016613265.1	60.0	6.7
Pseudomonas sp.	MF6768	GCA_016405545.1	60.6	7.0
Pseudomonas sp.	MF7448	GCA_016613085.1	60.7	7.4
Pseudomonas sp.	MGG07886_1680015496	GCA_949840575.1	60.7	6.6
Pseudomonas sp.	MGYG-HGUT-01279	GCA_902373485.1	62.1	6.6
Pseudomonas sp.	MLJ.1	GCA_023253375.1	60.8	7.0
Pseudomonas sp.	MN1F	GCA_009391885.1	62.9	7.1
Pseudomonas sp.	MNR3A	GCA_009650575.1	63.4	7.0
Pseudomonas sp.	MPBD4-3	GCA_002882875.1	60.9	7.1
Pseudomonas sp.	MPBD7-1	GCA_002901485.1	61.3	7.0
Pseudomonas sp.	MPR-ANC1	GCA_002901475.1	59.3	7.1
Pseudomonas sp.	MPR-AND1A	GCA_002883175.1	59.2	7.1
Pseudomonas sp.	MPR-AND1B	GCA_002884185.1	60.3	7.1
Pseudomonas sp.	MPR-E5	GCA_002883015.1	59.3	7.0
Pseudomonas sp.	MPR-LB3	GCA_002883315.1	60.3	7.4

Pseudomonas sp.	MPR-LB3	GCA_017349495.1	60.4	7.4
Pseudomonas sp.	MPR-LB5	GCA_002884025.1	60.3	7.5
Pseudomonas sp.	MPR-LB5	GCA_017349475.1	60.3	7.4
Pseudomonas sp.	MPR-R1B	GCA_002884015.1	64.2	5.8
Pseudomonas sp.	MPR-R2A2	GCA_002883065.1	59.3	5.6
Pseudomonas sp.	MPR-R2A3	GCA_002883105.1	60.5	5.7
Pseudomonas sp.	MPR-R2A3	GCA_017349435.1	60.5	6.7
Pseudomonas sp.	MPR-R2A4	GCA_017349415.1	60.3	6.7
Pseudomonas sp.	MPR-R2A4	GCA_002882575.1	60.3	6.6
Pseudomonas sp.	MPR-R2A5	GCA_017349395.1	60.3	6.7
Pseudomonas sp.	MPR-R2A6	GCA_002882635.1	60.4	6.6
Pseudomonas sp.	MPR-R2A7	GCA_017349455.1	60.3	6.6
Pseudomonas sp.	MPR-R3A	GCA_002883555.1	60.3	6.7
Pseudomonas sp.	MPR-R3B	GCA_002884125.1	59.4	6.6
Pseudomonas sp.	MPR-TSA4	GCA_002882115.1	59.3	6.6
Pseudomonas sp.	MWU12-2115	GCA_002909835.2	60.4	6.1
Pseudomonas sp.	MWU12-2534b	GCA_002915355.2	63.3	5.8
Pseudomonas sp.	MWU13-2100	GCA_023242365.1	61.3	5.9
Pseudomonas sp.	MWU15-20650	GCA_023242475.1	59.9	5.8
Pseudomonas sp.	N40	GCA_014472415.1	58.9	5.1
Pseudomonas sp.	NBRC 111119	GCA_001320125.1	62.3	5.9
Pseudomonas sp.	NBRC 111121	GCA_001320165.1	61.5	5.9
Pseudomonas sp.	NBRC 111122	GCA_001753915.1	61.7	6.0
Pseudomonas sp.	NBRC 111123	GCA_001320205.1	62.9	6.1
Pseudomonas sp.	NBRC 111124	GCA_001320245.1	62.4	6.1
Pseudomonas sp.	NBRC 111125	GCA_001320295.1	61.6	6.3
Pseudomonas sp.	NBRC 111127	GCA_001320345.1	62.5	0.2
Pseudomonas sp.	NBRC 111130	GCA_001320485.1	62.8	6.6
Pseudomonas sp.	NBRC 111131	GCA_001320525.1	61.8	6.6
Pseudomonas sp.	NBRC 111132	GCA_001320565.1	61.9	6.9
Pseudomonas sp.	NBRC 111133	GCA_001320605.1	61.4	6.7
Pseudomonas sp.	NBRC 111134	GCA_001320655.1	62.0	5.7
Pseudomonas sp.	NBRC 111135	GCA_001320705.1	64.5	3.8
Pseudomonas sp.	NBRC 111136	GCA_001320745.1	61.5	6.3
Pseudomonas sp.	NBRC 111137	GCA_001320785.1	59.8	4.3
Pseudomonas sp.	NBRC 111139	GCA_001753955.1	61.4	6.4
Pseudomonas sp.	NBRC 111141	GCA_001753975.1	62.0	3.0
Pseudomonas sp.	NBRC 111142	GCA_001320925.1	62.8	2.2
Pseudomonas sp.	NBRC 111143	GCA_001320965.1	62.8	6.1
Pseudomonas sp.	NDM	GCA_003105095.1	60.2	6.5
Pseudomonas sp.	NMI760_13	GCA_021283245.1	64.2	5.5
Pseudomonas sp.	NMI795_08	GCA_021282765.1	64.1	5.9
Pseudomonas sp.	NORP239	GCA_016764935.1	57.5	5.9
Pseudomonas sp.	NORP330	GCA_016763285.1	61.0	5.7
Pseudomonas sp.	NW5	GCA_023544715.1	65.3	6.9
Pseudomonas sp.	ODNR1LW	GCA_009905675.1	62.2	6.0
Pseudomonas sp.	OF001	GCA_904426495.1	68.0	6.2

Pseudomonas sp.	PA-1-2A	GCA_021609725.1	60.1	6.0
Pseudomonas sp.	PA-1-3F	GCA_021609665.1	60.1	6.1
Pseudomonas sp.	PA-1-5A	GCA_021609645.1	60.1	6.0
Pseudomonas sp.	PA-1-6A	GCA_021609505.1	60.1	4.7
Pseudomonas sp.	PA-1-6B	GCA_021609545.1	60.1	7.6
Pseudomonas sp.	PA-1-8C	GCA_021609475.1	60.1	6.1
Pseudomonas sp.	PA-3-10C	GCA_021608985.1	59.3	5.6
Pseudomonas sp.	PA-3-11C	GCA_021609035.1	59.4	6.6
Pseudomonas sp.	PA-3-5D	GCA_021608765.1	59.3	7.0
Pseudomonas sp.	PA-3-6H	GCA_021607225.1	59.3	6.7
Pseudomonas sp.	PA-4-8C	GCA_021606625.1	60.2	7.2
Pseudomonas sp.	PA-5-4B	GCA_021606215.1	60.0	5.9
Pseudomonas sp.	PA-5-4G	GCA_021606185.1	60.0	6.9
Pseudomonas sp.	PA-5-4H	GCA_021606165.1	60.0	7.4
Pseudomonas sp.	PA-6-1D	GCA_021605985.1	59.9	7.4
Pseudomonas sp.	PA-6-1H	GCA_021605925.1	59.9	7.4
Pseudomonas sp.	PA-6-2E	GCA_021605935.1	59.9	7.5
Pseudomonas sp.	PA-6-3C	GCA_021605825.1	59.9	5.7
Pseudomonas sp.	PA-6-3F	GCA_021605845.1	59.9	6.9
Pseudomonas sp.	PA-7-1E	GCA_021605325.1	60.0	7.3
Pseudomonas sp.	PAH14	GCA_018551555.1	65.1	7.3
Pseudomonas sp.	PAMC 25886	GCA_000242655.2	61.2	7.4
Pseudomonas sp.	ParCl1	GCA_947311875.1	62.5	7.5
Pseudomonas sp.	PB101	GCA_009764265.1	60.5	7.6
Pseudomonas sp.	PB103	GCA_009764635.1	59.2	7.4
Pseudomonas sp.	PB105	GCA_009764655.1	60.5	7.5
Pseudomonas sp.	PB120	GCA_009761815.1	59.8	7.4
Pseudomonas sp.	PDC86	GCA_900107325.1	60.6	5.9
Pseudomonas sp.	PDM13	GCA_025685765.1	66.3	6.4
Pseudomonas sp.	PE-S1G-1	GCA_002179905.1	61.1	6.9
Pseudomonas sp.	PGPPP1	GCA_002255865.1	60.1	6.7
Pseudomonas sp.	PGPR40	GCA_022014505.1	58.5	6.6
Pseudomonas sp.	PI1	GCA_000812405.1	66.7	6.1
Pseudomonas sp.	PIC25	GCA_002286785.1	63.6	6.1
Pseudomonas sp.	PICF141	GCA_002286815.1	58.8	5.4
Pseudomonas sp.	PNP	GCA_017897275.1	61.7	6.3
Pseudomonas sp.	PS01296	GCA_029959765.1	60.1	6.1
Pseudomonas sp.	PS01299	GCA_029960145.1	59.7	5.7
Pseudomonas sp.	PS01300	GCA_029960155.1	64.2	6.1
Pseudomonas sp.	PS01301	GCA_029960215.1	62.0	6.3
Pseudomonas sp.	PS01303	GCA_029959385.1	58.7	5.8
Pseudomonas sp.	PS02285	GCA_029959515.1	59.9	5.7
Pseudomonas sp.	PS02286	GCA_029959825.1	59.8	6.2
Pseudomonas sp.	PS02303	GCA_029959925.1	58.9	6.1
Pseudomonas sp.	PSB00027	GCA_016008985.1	61.7	6.5
Pseudomonas sp.	PSB00046	GCA_016337145.1	64.2	6.1
Pseudomonas sp.	PSB11	GCA_014596605.1	59.7	7.0

Pseudomonas sp.	JV551A1	GCA_900277145.1	62.8	6.6
Pseudomonas sp.	nbed6b101	GCA_902168275.1	62.6	6.7
Pseudomonas sp.	R2.Fl	GCA_023516795.1	62.7	6.4
Pseudomonas sp.	R2F_R2FSRR_metabat.47	GCA_023701025.1	72.4	6.2
Pseudomonas sp.	R2F_R2FSRR_metabat.60	GCA_023700865.1	67.1	6.3
Pseudomonas sp.	REP124	GCA_020164475.1	60.4	6.3
Pseudomonas sp.	RGIG3665	GCA_017455265.1	66.5	5.9
Pseudomonas sp.	RGIG627	GCA_017418485.1	62.6	6.4
Pseudomonas sp.	RGIG8546	GCA_017625305.1	58.2	6.2
Pseudomonas sp.	RGIG8787	GCA_017620955.1	58.7	3.9
Pseudomonas sp.	RGIG8810	GCA_017941955.1	57.7	3.2
Pseudomonas sp.	RGIG8834	GCA_017941465.1	58.8	2.6
Pseudomonas sp.	RGIG8837	GCA_017941425.1	59.0	9.4
Pseudomonas sp.	RGIG9111	GCA_017935815.1	58.8	4.5
Pseudomonas sp.	RGIG9204	GCA_017635815.1	57.9	6.0
Pseudomonas sp.	RGM3321	GCA_022836805.1	58.4	5.9
Pseudomonas sp.	RIT623	GCA_004519305.1	64.4	6.0
Pseudomonas sp.	RIT-PI-AD	GCA_025642785.1	66.2	6.0
Pseudomonas sp.	RL	GCA_000647775.1	65.6	6.0
Pseudomonas sp.	RQ_Bin_2	GCA_009360625.1	62.1	6.0
Pseudomonas sp.	RS261_metabat.bin.8	GCA_024709455.1	64.9	6.8
Pseudomonas sp.	RW10S2	GCA_014268985.1	63.3	6.7
Pseudomonas sp.	S 311-6	GCA_024128275.1	63.7	6.8
Pseudomonas sp.	S13.1.2	GCA_000292285.1	62.0	6.7
Pseudomonas sp.	S1C108_SP426	GCA_913777725.1	63.3	6.5
Pseudomonas sp.	S1C77_SP397	GCA_913775925.1	66.4	6.7
Pseudomonas sp.	S2C11432_SP223	GCA_913775285.1	63.4	6.6
Pseudomonas sp.	S2C311_SP75	GCA_913777285.1	64.5	6.7
Pseudomonas sp.	S2C3242_SP226	GCA_913776055.1	66.3	6.5
Pseudomonas sp.	S2C78296_SP133	GCA_913778435.1	63.3	6.5
Pseudomonas sp.	S31	GCA_016648105.1	63.5	6.5
Pseudomonas sp.	S32	GCA_016648095.1	62.2	6.5
Pseudomonas sp.	S33	GCA_016624455.1	66.4	6.5
Pseudomonas sp.	S36	GCA_016647875.1	62.3	6.4
Pseudomonas sp.	S3C519_SP188	GCA_913773655.1	60.8	7.5
Pseudomonas sp.	S44	GCA_016624805.1	63.4	7.0
Pseudomonas sp.	S60	GCA_016647865.1	62.3	5.3
Pseudomonas sp.	S68	GCA_016658555.1	66.4	6.8
Pseudomonas sp.	S75	GCA_016625345.1	63.9	6.7
Pseudomonas sp.	SDI	GCA_003105155.1	62.9	5.9
Pseudomonas sp.	SH10-3B	GCA_019891415.1	60.7	6.4
Pseudomonas sp.	SHC52	GCA_000801235.1	61.0	7.0
Pseudomonas sp.	SO_2017_LW2 bin 68	GCA_023228505.1	49.1	6.7
Pseudomonas sp.	SP131_2_metabat2 _genome_mining.13	GCA_913778735.1	66.1	7.0
Pseudomonas sp.	SP152_1_metabat2 _genome_mining.5	GCA_913776115.1	66.7	6.9
Pseudomonas sp.	SP200_1_metabat2	GCA_913777955.1	63.3	4.9

	_genome_mining.44			
Pseudomonas sp.	SP236_1_metabat2 _genome_mining.8	GCA_913775855.1	63.3	6.3
Pseudomonas sp.	SP29_3_metabat2 _genome_mining.23	GCA_913777085.1	66.5	7.2
Pseudomonas sp.	SP3_3_metabat2 _genome_mining.12	GCA_913774555.1	66.8	6.0
Pseudomonas sp.	SP403_2_metabat2 _genome_mining.12	GCA_913774965.1	66.7	6.0
Pseudomonas sp.	SP421_3_metabat2 _genome_mining.10	GCA_913778655.1	66.5	6.1
Pseudomonas sp.	SP67_1_metabat2 _genome_mining.20	GCA_913777505.1	62.2	6.5
Pseudomonas sp.	SRR17255085_bin.6 _metawrap_v1.3.0_MAG	GCA_946885775.1	60.3	6.9
Pseudomonas sp.	SSM44	GCA_003797905.1	56.2	5.8
Pseudomonas sp.	ST1	GCA_007352665.1	58.0	5.0
Pseudomonas sp.	SW.3.119	GCA_013824355.1	59.9	6.8
Pseudomonas sp.	SWRI154	GCA_014268645.1	60.5	6.5
Pseudomonas sp.	SWRI179	GCA_014268705.1	61.2	6.2
Pseudomonas sp.	SWRI50	GCA_014269095.1	62.4	7.5
Pseudomonas sp.	SWRI77	GCA_014269105.1	62.7	4.8
Pseudomonas sp.	SWRI81	GCA_014268545.1	60.1	5.7
Pseudomonas sp.	SWRI99	GCA_014270125.1	60.0	6.6
Pseudomonas sp.	T	GCA_002079985.1	65.2	5.9
Pseudomonas sp.	TH05	GCA_016651325.1	62.5	5.4
Pseudomonas sp.	TH10	GCA_016651255.1	59.4	9.7
Pseudomonas sp.	TMW22089	GCA_022496645.1	58.7	1.8
Pseudomonas sp.	TTU2014-105ASC	GCA_001446975.1	61.7	4.0
Pseudomonas sp.	UK4	GCA_000174915.1	60.4	6.7
Pseudomonas sp.	UMA601	GCA_013337485.1	67.4	4.8
Pseudomonas sp.	UMA603	GCA_013337585.1	67.4	2.5
Pseudomonas sp.	UMA643	GCA_013337595.1	67.4	3.7
Pseudomonas sp.	UMC3103	GCA_013337545.1	67.4	4.5
Pseudomonas sp.	UMC3106	GCA_013337565.1	67.5	3.2
Pseudomonas sp.	UMC3129	GCA_013337495.1	67.4	4.8
Pseudomonas sp.	UMC631	GCA_013337505.1	67.4	3.0
Pseudomonas sp.	UMC76	GCA_014170435.1	67.3	2.9
Pseudomonas sp.	V104_6	GCA_029967935.1	61.8	3.7
Pseudomonas sp.	V98_8	GCA_029967975.1	60.0	6.4
Pseudomonas sp.	VE266-1	GCA_024126735.1	64.4	5.4
Pseudomonas sp.	VI4.1	GCA_002029345.1	60.0	4.8
Pseudomonas sp.	W15Feb9B	GCA_000828175.1	60.4	3.8
Pseudomonas sp.	WP001	GCA_002911525.1	60.5	6.3
Pseudomonas sp.	WS 5018	GCA_012985885.1	63.8	4.0
Pseudomonas sp.	WS 5071	GCA_012985765.1	57.9	5.6
Pseudomonas sp.	WS 5407	GCA_012985405.1	59.8	9.8
Pseudomonas sp.	WS 5414	GCA_012985795.1	62.4	5.0

Pseudomonas sp.	Wu6	GCA_019891515.1	60.5	5.4
Pseudomonas sp.	ZH-FAD	GCA_002803095.1	62.2	5.0

Table S3

List of downloaded 509 *Pseudomonas* spp. genomes (Scaffold) to analyze in this study

Species name	Strain	Accession no	GC Content (%)	Genome Length (mb)
Pseudomonas sp.	ICMP 3272	GCA_001466905.1	58.5	5.9
Pseudomonas sp.	COW5	GCA_026240005.1	60.5	6.4
Pseudomonas sp.	LFM046	GCA_000949385.2	64.3	6.0
Pseudomonas sp.	HS-2	GCA_004369025.1	62.1	6.4
Pseudomonas sp.	9AZ	GCA_902506525.1	59.7	4.5
Pseudomonas sp.	NFPP19	GCA_900110785.1	63.4	6.9
Pseudomonas sp.	Irchel 3H9	GCA_900187495.1	59.8	6.1
Pseudomonas sp.	SJZ131	GCA_007828705.1	59.6	6.1
Pseudomonas sp.	Leaf129	GCA_001423465.1	61.9	4.9
Pseudomonas sp.	B40(2017)	GCA_002112855.1	60.8	6.2
Pseudomonas sp.	B38(2017)	GCA_002113625.1	60.7	6.3
Pseudomonas sp.	B34(2017)	GCA_002112915.1	60.7	6.3
Pseudomonas sp.	B32(2017)	GCA_002112925.1	60.7	6.3
Pseudomonas sp.	NFPP16	GCA_900119675.1	63.5	6.8
Pseudomonas sp.	C9	GCA_002000165.1	58.9	6.4
Pseudomonas sp.	P116	GCA_020149425.1	58.9	6.2
Pseudomonas sp.	MG-9	GCA_018729515.1	60.3	6.2
Pseudomonas sp.	Irchel s3a10	GCA_900187525.1	60.3	6.0
Pseudomonas sp.	R41(2017)	GCA_002112365.1	60.3	6.3
Pseudomonas sp.	R38(2017)	GCA_002112395.1	60.3	6.3
Pseudomonas sp.	79_C	GCA_017491755.1	60.2	6.2
Pseudomonas sp.	NFPP25	GCA_900116505.1	63.5	6.8
Pseudomonas sp.	NFPP18	GCA_900109335.1	63.5	6.8
Pseudomonas sp.	NFPP14	GCA_900119635.1	63.5	6.8
Pseudomonas sp.	8O	GCA_902506495.1	62.5	5.2
Pseudomonas sp.	B28(2017)	GCA_002113025.1	59.9	5.9
Pseudomonas sp.	WPR_5_2	GCA_003634475.1	59.5	6.8
Pseudomonas sp.	R40(2017)	GCA_002113365.1	60.3	6.3
Pseudomonas sp.	R33(2017)	GCA_002112455.1	60.3	6.3
Pseudomonas sp.	Leaf127	GCA_001423155.1	62.2	6.4
Pseudomonas sp.	NFPP33	GCA_900103725.1	62.6	5.3
Pseudomonas sp.	BLCC-B112	GCA_028550695.1	61.9	6.8
Pseudomonas sp.	B30(2017)	GCA_002112995.1	60.7	6.3
Pseudomonas sp.	NFR02	GCA_900119375.1	60.3	5.8
Pseudomonas sp.	R43(2017)	GCA_002112345.1	60.6	6.4
Pseudomonas sp.	Irchel 3F6	GCA_900187595.1	60.0	5.9
Pseudomonas sp.	GV047	GCA_003058245.1	61.0	6.9
Pseudomonas sp.	NFPP13	GCA_900111855.1	63.5	6.8
Pseudomonas sp.	Irchel 3F3	GCA_900187545.1	62.1	6.0
Pseudomonas sp.	JAI111	GCA_024809425.1	59.0	6.8
Pseudomonas sp.	8Z	GCA_902506535.1	60.9	4.8
Pseudomonas sp.	R36(2017)	GCA_002112425.1	60.3	6.3
Pseudomonas sp.	LP_7_YM	GCA_004364435.1	58.8	5.4
Pseudomonas sp.	8AS	GCA_902506505.1	65.8	4.3

Pseudomonas sp.	CFBP8771	GCA_014841695.1	62.1	4.8
Pseudomonas sp.	B22(2017)	GCA_002113105.1	62.8	5.6
Pseudomonas sp.	NFPP15	GCA_900103335.1	63.5	6.8
Pseudomonas sp.	TH34	GCA_016650865.1	60.5	7.0
Pseudomonas sp.	R9.37	GCA_003014915.1	60.6	6.6
Pseudomonas sp.	HPB0071	GCA_000478505.2	55.1	5.7
Pseudomonas sp.	OG7	GCA_014192825.1	62.0	5.8
Pseudomonas sp.	Snoq117.2	GCA_900110545.1	64.8	5.5
Pseudomonas sp.	LAP_36	GCA_019456785.1	60.0	6.1
Pseudomonas sp.	2848	GCA_003253945.1	64.2	5.8
Pseudomonas sp.	203-8	GCA_020708595.1	66.4	6.5
Pseudomonas sp.	NFR16	GCA_900108875.1	60.2	6.1
Pseudomonas sp.	R34(2017)	GCA_002113425.1	60.6	6.4
Pseudomonas sp.	CFBP13528	GCA_005233615.1	60.4	6.0
Pseudomonas sp.	MYb185	GCA_002979975.1	61.5	4.1
Pseudomonas sp.	C 49-2	GCA_003970195.1	60.1	6.4
Pseudomonas sp.	Irchel s3b2	GCA_900187445.1	58.7	6.2
Pseudomonas sp.	18081308	GCA_024718375.1	66.4	6.4
Pseudomonas sp.	31 E 5	GCA_900005815.1	60.8	5.9
Pseudomonas sp.	MYb60	GCA_002979555.1	61.1	5.6
Pseudomonas sp.	95_A	GCA_017491685.1	60.0	6.1
Pseudomonas sp.	Root562	GCA_001427125.1	59.0	6.2
Pseudomonas sp.	R23(2017)	GCA_002112575.1	60.5	6.3
Pseudomonas sp.	R24(2017)	GCA_002113465.1	60.5	6.3
Pseudomonas sp.	R15(2017)	GCA_002112685.1	60.5	6.3
Pseudomonas sp.	Irchel 3A5	GCA_900187575.1	57.1	6.4
Pseudomonas sp.	43MFCvi1.1	GCA_900115935.1	60.9	6.7
Pseudomonas sp.	NFPP04	GCA_900113815.1	61.0	6.3
Pseudomonas sp.	Irchel 3F5	GCA_900187625.1	62.3	5.9
Pseudomonas sp.	TH31	GCA_016650815.1	59.0	7.2
Pseudomonas sp.	Irchel s3h14	GCA_900187425.1	58.9	6.7
Pseudomonas sp.	RGB	GCA_007713445.1	60.5	6.3
Pseudomonas sp.	MS15	GCA_013277805.1	65.1	5.2
Pseudomonas sp.	BRG-100	GCA_000737955.1	59.6	6.2
Pseudomonas sp.	35 E 8	GCA_900005945.1	59.8	6.4
Pseudomonas sp.	R35(2017)	GCA_002112445.1	60.6	6.4
Pseudomonas sp.	AAC	GCA_000725445.1	67.0	7.1
Pseudomonas sp.	JAI115	GCA_014200835.1	60.1	6.5
Pseudomonas sp.	MYb13	GCA_002980075.1	60.9	6.1
Pseudomonas sp.	GV105	GCA_003058185.1	61.2	6.6
Pseudomonas sp.	Irchel s3f10	GCA_900187515.1	59.1	6.5
Pseudomonas sp.	Irchel 3E20	GCA_900187645.1	62.3	5.3
Pseudomonas sp.	Irchel 3A18	GCA_900187465.1	58.2	5.9
Pseudomonas sp.	L-22-4S-12	GCA_009799935.1	65.8	4.5
Pseudomonas sp.	OK266	GCA_900110195.1	59.2	6.6
Pseudomonas sp.	SJZ103	GCA_007828335.1	60.4	6.7
Pseudomonas sp.	R11(2017)	GCA_002112695.1	63.8	6.0

Pseudomonas sp.	B5(2017)	GCA_002112825.1	63.4	5.9
Pseudomonas sp.	GV085	GCA_003050925.1	59.6	6.4
Pseudomonas sp.	R28(2017)	GCA_002112505.1	63.8	6.0
Pseudomonas sp.	NFPP07	GCA_900115645.1	62.9	6.7
Pseudomonas sp.	R17(2017)	GCA_002112665.1	63.8	6.0
Pseudomonas sp.	R9(2017)	GCA_002113325.1	63.8	6.0
Pseudomonas sp.	N040	GCA_015637705.1	63.0	3.7
Pseudomonas sp.	JUb52	GCA_004341115.1	65.5	5.1
Pseudomonas sp.	NFACC25	GCA_900100935.1	61.2	6.7
Pseudomonas sp.	SbOxS1	GCA_013416725.1	60.4	7.4
Pseudomonas sp.	Irchel s3a12	GCA_900187485.1	59.1	6.5
Pseudomonas sp.	10B238	GCA_000970615.1	60.2	4.9
Pseudomonas sp.	Leaf83	GCA_001422075.1	62.4	5.5
Pseudomonas sp.	SJZ094	GCA_007828435.1	60.4	6.7
Pseudomonas sp.	21_B	GCA_017490865.1	60.3	6.2
Pseudomonas sp.	B6(2017)	GCA_002112765.1	63.4	7.1
Pseudomonas sp.	69_B	GCA_017490845.1	60.1	6.1
Pseudomonas sp.	Irchel s3h9	GCA_900187475.1	59.1	6.5
Pseudomonas sp.	SCPG-7	GCA_002156405.1	60.1	6.3
Pseudomonas sp.	Leaf48	GCA_001421885.1	59.4	5.7
Pseudomonas sp.	R-28-1W-6	GCA_009799925.1	65.8	4.3
Pseudomonas sp.	R5(2017)	GCA_002112255.1	64.8	6.2
Pseudomonas sp.	SBB6	GCA_024213215.1	62.4	5.7
Pseudomonas sp.	Irchel s3f19	GCA_900187555.1	62.6	6.0
Pseudomonas sp.	Root569	GCA_001427465.1	59.9	6.2
Pseudomonas sp.	B7(2017)	GCA_002112745.1	64.7	6.3
Pseudomonas sp.	R21(2017)	GCA_002112635.1	64.8	6.2
Pseudomonas sp.	R12(2017)	GCA_002112705.1	64.8	6.2
Pseudomonas sp.	R27(2017)	GCA_002113445.1	64.8	6.2
Pseudomonas sp.	R19(2017)	GCA_002113485.1	64.8	6.2
Pseudomonas sp.	86_A	GCA_017491705.1	60.3	6.2
Pseudomonas sp.	DE0216	GCA_007677725.1	62.5	5.1
Pseudomonas sp.	NFACC32-1	GCA_900101215.1	62.0	5.9
Pseudomonas sp.	NFACC08-1	GCA_900106735.1	60.6	6.7
Pseudomonas sp.	P20	GCA_016463925.1	65.9	6.8
Pseudomonas sp.	MCal1	GCA_026341415.1	60.5	6.2
Pseudomonas sp.	B707	GCA_022496685.1	60.7	6.3
Pseudomonas sp.	MS19	GCA_013277615.1	57.2	4.5
Pseudomonas sp.	Irchel s3h17	GCA_900187505.1	60.5	5.5
Pseudomonas sp.	B26(2017)	GCA_002113045.1	60.4	6.2
Pseudomonas sp.	ICMP22404	GCA_006227205.1	60.7	6.7
Pseudomonas sp.	B23(2017)	GCA_002113725.1	63.4	5.9
Pseudomonas sp.	R16(2017)	GCA_002113495.1	64.7	6.3
Pseudomonas sp.	MT4	GCA_015956425.1	60.7	4.7
Pseudomonas sp.	PD9R	GCA_019200975.1	58.4	6.9
Pseudomonas sp.	B3(2017)	GCA_002113595.1	64.8	6.2
Pseudomonas sp.	R14(2017)	GCA_002113525.1	63.8	6.0

Pseudomonas sp.	R10(2017)	GCA_002113545.1	63.7	6.0
Pseudomonas sp.	EpSL25	GCA_001512295.1	65.5	5.4
Pseudomonas sp.	CP-1	GCA_021213715.1	65.6	7.0
Pseudomonas sp.	NP21570	GCA_020523865.1	62.4	4.7
Pseudomonas sp.	DE0038	GCA_007680295.1	61.7	4.7
Pseudomonas sp.	R20(2017)	GCA_002112625.1	64.8	6.2
Pseudomonas sp.	R29(2017)	GCA_002112515.1	64.8	6.2
Pseudomonas sp.	R44(2017)	GCA_002113335.1	64.8	6.2
Pseudomonas sp.	R25(2017)	GCA_002112565.1	64.8	6.2
Pseudomonas sp.	NFACC39-1	GCA_900110355.1	60.4	6.1
Pseudomonas sp.	Irchel 3A7	GCA_900187565.1	59.5	6.5
Pseudomonas sp.	P22	GCA_016464635.1	65.9	6.8
Pseudomonas sp.	B15(2017)	GCA_002113215.1	60.4	6.4
Pseudomonas sp.	71_D	GCA_017490875.1	60.0	6.1
Pseudomonas sp.	100_A	GCA_017491715.1	60.1	6.1
Pseudomonas sp.	ICMP 561	GCA_002699985.1	57.0	6.3
Pseudomonas sp.	78_B	GCA_017490815.1	60.1	6.4
Pseudomonas sp.	R37(2017)	GCA_002113375.1	60.1	6.0
Pseudomonas sp.	FBF18	GCA_024261405.1	64.0	5.7
Pseudomonas sp.	o96	GCA_003935455.1	62.4	5.0
Pseudomonas sp.	R26(2017)	GCA_002112545.1	63.4	7.1
Pseudomonas sp.	Irchel s3b6	GCA_900187415.1	60.4	5.4
Pseudomonas sp.	50_B	GCA_017491625.1	62.4	6.9
Pseudomonas sp.	DE0021	GCA_007680535.1	61.7	4.9
Pseudomonas sp.	B20(2017)	GCA_002113125.1	60.5	6.1
Pseudomonas sp.	B35(2017)	GCA_002113645.1	64.7	6.2
Pseudomonas sp.	B19(2017)	GCA_002113165.1	63.0	5.6
Pseudomonas sp.	B18(2017)	GCA_002113185.1	63.0	5.6
Pseudomonas sp.	CK-NBRI-02	GCA_008124495.1	62.5	5.3
Pseudomonas sp.	B17(2017)	GCA_002113765.1	60.5	6.1
Pseudomonas sp.	B2(2017)	GCA_002113685.1	64.7	6.3
Pseudomonas sp.	BN505	GCA_029873175.1	62.2	6.3
Pseudomonas sp.	32.2.56	GCA_024655605.1	60.6	4.4
Pseudomonas sp.	NFACC36	GCA_900119445.1	62.0	6.0
Pseudomonas sp.	Root329	GCA_001424925.1	59.1	6.7
Pseudomonas sp.	GZJR-8	GCA_002916705.1	60.0	5.9
Pseudomonas sp.	RV120224-01b	GCA_003208145.1	62.4	6.0
Pseudomonas sp.	RV120224-01c	GCA_003208135.1	62.4	6.0
Pseudomonas sp.	58(2021)	GCA_017491585.1	60.4	6.3
Pseudomonas sp.	OK272	GCA_900109995.1	61.4	5.3
Pseudomonas sp.	MIL9	GCA_016887925.1	59.5	6.8
Pseudomonas sp.	B11(2017)	GCA_002113285.1	64.8	6.2
Pseudomonas sp.	EZ-C24	GCA_014525325.1	62.2	5.2
Pseudomonas sp.	KSR10	GCA_022355455.1	61.1	4.9
Pseudomonas sp.	MYb2	GCA_002979955.1	59.3	6.4
Pseudomonas sp.	NFACC09-4	GCA_900119215.1	62.0	6.0
Pseudomonas sp.	PLB05	GCA_021106615.1	65.2	5.1

Pseudomonas sp.	8BK	GCA_902506475.1	60.2	4.5
Pseudomonas sp.	BRM28	GCA_002939405.1	64.4	5.9
Pseudomonas sp.	JAI120	GCA_014207255.1	60.4	6.9
Pseudomonas sp.	SJZ073	GCA_014207275.1	60.4	6.9
Pseudomonas sp.	9Ag	GCA_902506445.1	60.5	4.7
Pseudomonas sp.	NFPP12	GCA_900103205.1	63.3	7.0
Pseudomonas sp.	HR1	GCA_030175875.1	66.1	5.1
Pseudomonas sp.	NFPP17	GCA_900102935.1	63.5	6.8
Pseudomonas sp.	NFPP09	GCA_900119575.1	63.4	7.0
Pseudomonas sp.	NFPP05	GCA_900114815.1	63.3	7.0
Pseudomonas sp.	NFPP10	GCA_900109535.1	63.4	7.0
Pseudomonas sp.	NFPP08	GCA_900113795.1	63.4	7.0
Pseudomonas sp.	P42	GCA_017973755.1	58.8	6.1
Pseudomonas sp.	P179	GCA_000478485.2	65.6	6.9
Pseudomonas sp.	D2002	GCA_013385735.1	60.3	6.6
Pseudomonas sp.	B8(2017)	GCA_002113575.1	63.4	5.9
Pseudomonas sp.	OV226	GCA_003148705.1	58.6	6.8
Pseudomonas sp.	74_A	GCA_017491885.1	60.0	6.1
Pseudomonas sp.	SJZ083	GCA_007828235.1	59.3	6.6
Pseudomonas sp.	SJZ077	GCA_007993905.1	59.3	6.6
Pseudomonas sp.	B13(2017)	GCA_002113245.1	63.5	6.0
Pseudomonas sp.	B12(2017)	GCA_002113785.1	63.5	6.0
Pseudomonas sp.	B4(2017)	GCA_002113565.1	63.5	5.9
Pseudomonas sp.	NCCP-436	GCA_019656335.1	61.4	3.7
Pseudomonas sp.	NFACC05-1	GCA_900100135.1	60.7	6.7
Pseudomonas sp.	NFACC04-2	GCA_900119265.1	61.3	6.4
Pseudomonas sp.	B10(2017)	GCA_002113295.1	63.1	5.7
Pseudomonas sp.	31 E 6	GCA_900005935.1	60.7	5.9
Pseudomonas sp.	B9(2017)	GCA_002112755.1	63.0	5.6
Pseudomonas sp.	5	GCA_000955815.1	59.6	5.6
Pseudomonas sp.	NBRC 100443	GCA_030268185.1	67.0	7.2
Pseudomonas sp.	OK602	GCA_900114855.1	61.4	5.3
Pseudomonas sp.	BBP2017	GCA_003024385.1	60.3	5.0
Pseudomonas sp.	B24(2017)	GCA_002113085.1	63.4	5.9
Pseudomonas sp.	BN605	GCA_029873135.1	61.9	6.6
Pseudomonas sp.	52 E 6	GCA_900004655.1	60.0	6.3
Pseudomonas sp.	NFACC14	GCA_900106885.1	61.3	6.8
Pseudomonas sp.	LS-2	GCA_003605735.1	59.1	6.9
Pseudomonas sp.	MYb3	GCA_002979735.1	59.3	6.4
Pseudomonas sp.	24 R 17	GCA_900004795.1	59.8	6.4
Pseudomonas sp.	B14(2017)	GCA_002113255.1	63.4	5.9
Pseudomonas sp.	ARP3	GCA_001029595.1	60.3	6.1
Pseudomonas sp.	DE0157	GCA_007678615.1	61.7	4.6
Pseudomonas sp.	IPO3747	GCA_013385245.1	60.0	6.5
Pseudomonas sp.	BN414	GCA_029871275.1	63.1	6.7
Pseudomonas sp.	NFACC13-1	GCA_900104425.1	61.2	7.0
Pseudomonas sp.	TN43	GCA_014192635.1	58.8	6.0

Pseudomonas sp.	478	GCA_003254125.1	58.9	6.4
Pseudomonas sp.	NFPP24	GCA_900112095.1	61.0	6.3
Pseudomonas sp.	NFPP11	GCA_900113895.1	61.0	6.3
Pseudomonas sp.	Irchel 3H7	GCA_900187605.1	59.0	6.5
Pseudomonas sp.	Irchel 3E13	GCA_900187455.1	62.7	7.2
Pseudomonas sp.	B16(2017)	GCA_002113205.1	60.7	6.2
Pseudomonas sp.	B7	GCA_016735645.1	60.7	6.3
Pseudomonas sp.	CBMAI 2609	GCA_029872515.1	65.5	5.3
Pseudomonas sp.	MB04B	GCA_023094205.1	62.0	6.0
Pseudomonas sp.	24 E 1	GCA_900005875.1	60.0	6.4
Pseudomonas sp.	Root68	GCA_001427765.1	60.0	6.3
Pseudomonas sp.	Root71	GCA_001429045.1	60.0	6.2
Pseudomonas sp.	NFACC10-1	GCA_900119195.1	59.4	5.8
Pseudomonas sp.	58 R 12	GCA_900005865.1	59.9	6.3
Pseudomonas sp.	NFR09	GCA_900111535.1	60.7	6.6
Pseudomonas sp.	KD5	GCA_012932895.1	59.3	6.5
Pseudomonas sp.	P9(2020)	GCA_017973695.1	58.7	6.5
Pseudomonas sp.	MG-2	GCA_018729535.1	62.9	5.8
Pseudomonas sp.	HMSC057H01	GCA_001836405.1	66.1	6.7
Pseudomonas sp.	21615526	GCA_013386245.1	61.1	6.8
Pseudomonas sp.	P7779	GCA_013386315.1	61.1	6.8
Pseudomonas sp.	C3-2018	GCA_021083555.1	61.4	5.7
Pseudomonas sp.	AU10	GCA_025699425.1	59.8	6.5
Pseudomonas sp.	HMSC072F09	GCA_001809805.1	66.0	6.8
Pseudomonas sp.	AKS31	GCA_023634765.1	58.6	6.7
Pseudomonas sp.	44 R 15	GCA_900004965.1	60.5	6.1
Pseudomonas sp.	CG7	GCA_023701885.1	60.8	6.8
Pseudomonas sp.	HMSC16B01	GCA_001808305.1	66.1	6.9
Pseudomonas sp.	EGD-AKN5	GCA_001669755.1	67.0	7.1
Pseudomonas sp.	BN417	GCA_029871205.1	64.6	6.6
Pseudomonas sp.	Sample_10	GCA_004321495.1	59.1	6.0
Pseudomonas sp.	p99	GCA_003936655.1	62.4	5.6
Pseudomonas sp.	HMSC076A11	GCA_001810205.1	66.3	6.0
Pseudomonas sp.	BIOMIG1N	GCA_001705835.1	62.2	7.5
Pseudomonas sp.	34 E 7	GCA_900005715.1	60.4	6.3
Pseudomonas sp.	P7758	GCA_013386485.1	61.2	6.9
Pseudomonas sp.	BIOMIG1BDMA	GCA_001705845.1	62.2	7.5
Pseudomonas sp.	Sample_16	GCA_004307665.1	59.1	6.3
Pseudomonas sp.	DrBHI1	GCA_002205115.1	64.3	5.7
Pseudomonas sp.	24 E 13	GCA_900004715.1	60.5	6.0
Pseudomonas sp.	BIOMIG1BD	GCA_001705885.1	62.2	7.5
Pseudomonas sp.	TJI-51	GCA_000190455.3	62.1	5.8
Pseudomonas sp.	HMWF034	GCA_003060935.1	59.8	6.1
Pseudomonas sp.	RW407	GCA_003176655.1	67.4	7.3
Pseudomonas sp.	Irchel s3a18	GCA_900187535.1	60.4	5.5
Pseudomonas sp.	SJZ080	GCA_007828295.1	59.2	6.8
Pseudomonas sp.	R4(2017)	GCA_002112285.1	64.7	6.8

Pseudomonas sp.	30_B	GCA_017491605.1	65.0	6.2
Pseudomonas sp.	58 R 3	GCA_900006605.1	60.1	6.7
Pseudomonas sp.	IPO3749	GCA_013387085.1	60.5	6.9
Pseudomonas sp.	25 R 14	GCA_900004755.1	60.2	6.3
Pseudomonas sp.	W2Aug9	GCA_023127755.1	59.9	6.0
Pseudomonas sp.	SZ57	GCA_009495975.1	59.2	6.0
Pseudomonas sp.	NFACC07-1	GCA_900109015.1	60.8	6.7
Pseudomonas sp.	BN606	GCA_029873115.1	62.7	6.4
Pseudomonas sp.	TDA1	GCA_009737195.1	62.5	5.9
Pseudomonas sp.	p106	GCA_003936645.1	61.9	5.6
Pseudomonas sp.	PA27	GCA_001945445.1	63.1	5.4
Pseudomonas sp.	MYb115	GCA_002980155.1	60.5	6.3
Pseudomonas sp.	382	GCA_002759075.1	62.4	6.3
Pseudomonas sp.	B6002	GCA_013385985.1	61.3	6.7
Pseudomonas sp.	S12(2018)	GCA_024355235.1	64.3	5.5
Pseudomonas sp.	H3(2019)	GCA_007713465.1	58.8	5.9
Pseudomonas sp.	W2Jun17	GCA_023127795.1	59.9	6.1
Pseudomonas sp.	Root401	GCA_001425105.1	60.5	7.0
Pseudomonas sp.	CC120222-01a	GCA_003097235.1	62.7	6.2
Pseudomonas sp.	ICMP 460	GCA_002723595.1	59.5	6.1
Pseudomonas sp.	51_B	GCA_017491595.1	60.7	5.3
Pseudomonas sp.	Pse79	GCA_024519615.1	61.2	6.6
Pseudomonas sp.	ML96	GCA_000761545.1	64.8	4.8
Pseudomonas sp.	1 R 17	GCA_900067035.1	60.6	7.0
Pseudomonas sp.	D4002	GCA_013385665.1	60.9	7.1
Pseudomonas sp.	AF76	GCA_003732845.1	62.0	5.9
Pseudomonas sp.	NFACC24-1	GCA_900115235.1	61.1	6.6
Pseudomonas sp.	N2-11	GCA_024213525.1	61.6	5.1
Pseudomonas sp.	KBW05	GCA_003852405.1	60.9	6.5
Pseudomonas sp.	Larv2_ips	GCA_003590475.1	60.5	6.7
Pseudomonas sp.	NFACC15-1	GCA_900103555.1	61.3	6.8
Pseudomonas sp.	EKM23D	GCA_011800525.1	60.6	5.8
Pseudomonas sp.	Irchel 3H3	GCA_900187635.1	61.2	6.0
Pseudomonas sp.	B6001	GCA_013386015.1	61.1	6.9
Pseudomonas sp.	Irchel s3f7	GCA_900187615.1	59	6.1
Pseudomonas sp.	LB-090624	GCA_003205205.1	62.1	5.7
Pseudomonas sp.	AL03	GCA_029960815.1	58.9	5.9
Pseudomonas sp.	Q11	GCA_024494355.1	60.2	6.2
Pseudomonas sp.	s199	GCA_003935435.1	63.1	4.3
Pseudomonas sp.	SJZ079	GCA_007993865.1	62.2	5.0
Pseudomonas sp.	C6002	GCA_013385805.1	61.1	6.8
Pseudomonas sp.	PA1	GCA_001945375.1	63.1	5.3
Pseudomonas sp.	BN607	GCA_029871175.1	62.1	5.9
Pseudomonas sp.	CF161	GCA_000416215.1	62.6	6.9
Pseudomonas sp.	NCIMB 10586	GCA_023127895.1	59.8	6.2
Pseudomonas sp.	21	GCA_000955805.1	65.6	6.3
Pseudomonas sp.	BN415	GCA_029871195.1	63.2	6.1

Pseudomonas sp.	21LCFQ02	GCA_024129895.1	60.7	6.4
Pseudomonas sp.	NFACC37-1	GCA_900101575.1	61.0	6.7
Pseudomonas sp.	Irchel s3b5	GCA_900187435.1	58.9	6.0
Pseudomonas sp.	C8002	GCA_013385765.1	61.0	6.9
Pseudomonas sp.	W15Feb34	GCA_023127765.1	59.8	6.1
Pseudomonas sp.	BCRC 81390	GCA_030341915.1	63.1	5.5
Pseudomonas sp.	Pse55	GCA_024519575.1	61.2	6.6
Pseudomonas sp.	MR 02	GCA_002797475.1	62.6	5.9
Pseudomonas sp.	B329	GCA_023127835.1	59.6	6.4
Pseudomonas sp.	D6002	GCA_013385625.1	61.1	6.8
Pseudomonas sp.	HMWF032	GCA_003060885.1	60.0	4.4
Pseudomonas sp.	LJDD11	GCA_024584215.1	60.6	6.3
Pseudomonas sp.	BN411	GCA_029871235.1	63.6	6.4
Pseudomonas sp.	SJZ101	GCA_007828415.1	60.7	6.1
Pseudomonas sp.	TNT19	GCA_023072615.1	58.6	6.5
Pseudomonas sp.	PAGU 2196	GCA_022179005.1	62.3	6.2
Pseudomonas sp.	37 R 15	GCA_900004865.1	60.4	6.3
Pseudomonas sp.	SJZ074	GCA_007828215.1	60.7	6.2
Pseudomonas sp.	CF150	GCA_000416175.1	59.8	6.1
Pseudomonas sp.	E6002	GCA_013385565.1	61.1	6.9
Pseudomonas sp.	460	GCA_004346485.1	58.8	6.6
Pseudomonas sp.	FeS53a	GCA_000931385.1	67.3	5.9
Pseudomonas sp.	UFMG81	GCA_015354575.1	64.0	6.1
Pseudomonas sp.	P7759	GCA_013386445.1	60.2	6.7
Pseudomonas sp.	DE0010	GCA_007680745.1	61.8	4.8
Pseudomonas sp.	SJZ085	GCA_007828275.1	60.7	6.2
Pseudomonas sp.	NFACC02	GCA_900110765.1	59.6	6.2
Pseudomonas sp.	AL15	GCA_029960625.1	59.9	6.4
Pseudomonas sp.	BN102	GCA_029871245.1	64.6	6.5
Pseudomonas sp.	RW409	GCA_003184125.1	62.5	7.2
Pseudomonas sp.	SJZ075	GCA_007828175.1	60.7	6.1
Pseudomonas sp.	SJZ124	GCA_007828495.1	60.7	6.1
Pseudomonas sp.	A4002	GCA_013386105.1	61.1	6.8
Pseudomonas sp.	NFACC06-1	GCA_900112875.1	61.0	6.4
Pseudomonas sp.	C1C7	GCA_013359595.1	60.9	6.7
Pseudomonas sp.	IPO3775	GCA_013385215.1	61.0	6.9
Pseudomonas sp.	QTF5	GCA_000512695.2	58.7	6.0
Pseudomonas sp.	Sample_22	GCA_004307635.1	59.0	6.2
Pseudomonas sp.	AL10	GCA_029960685.1	59.9	6.4
Pseudomonas sp.	165	GCA_030328985.1	61.6	6.8
Pseudomonas sp.	RW405	GCA_003184135.1	61.8	5.9
Pseudomonas sp.	43(2021)	GCA_017490805.1	62.9	5.9
Pseudomonas sp.	D5002	GCA_013385605.1	61.0	7.2
Pseudomonas sp.	8 R 14	GCA_900005955.1	60.9	6.9
Pseudomonas sp.	BN515	GCA_029873155.1	63.9	6.3
Pseudomonas sp.	F8002	GCA_013385495.1	61.0	7.0
Pseudomonas sp.	G1002	GCA_013385455.1	61.1	6.9

Pseudomonas sp.	SJZ078	GCA_007828255.1	60.6	6.2
Pseudomonas sp.	G5001	GCA_013385475.1	60.9	7.0
Pseudomonas sp.	LP_4_YM	GCA_004346335.1	61.4	5.3
Pseudomonas sp.	TAE6080	GCA_018732265.1	60.0	6.4
Pseudomonas sp.	Root9	GCA_001429205.1	59.7	6.6
Pseudomonas sp.	Pse35	GCA_024519715.1	60.1	5.9
Pseudomonas sp.	25 E 4	GCA_900004645.1	60.9	5.6
Pseudomonas sp.	F9001	GCA_013385435.1	61.1	6.8
Pseudomonas sp.	OA65	GCA_017498305.1	60.8	6.8
Pseudomonas sp.	ICMP 8385	GCA_002699915.1	60.5	6.8
Pseudomonas sp.	UMAB-08	GCA_900095225.1	57.9	5.6
Pseudomonas sp.	D8002	GCA_013385695.1	61.0	6.9
Pseudomonas sp.	RIT778	GCA_019718735.1	60.7	6.4
Pseudomonas sp.	I8001	GCA_013385345.1	61.1	6.9
Pseudomonas sp.	28 E 9	GCA_900004785.1	60.5	6.2
Pseudomonas sp.	IPO3774	GCA_013386835.1	60.8	7.1
Pseudomonas sp.	PA15	GCA_001945395.1	63.2	5.5
Pseudomonas sp.	UMAB-40	GCA_900095275.1	59.0	6.9
Pseudomonas sp.	F01002	GCA_004402115.1	58.7	6.5
Pseudomonas sp.	IPO3778	GCA_013386825.1	60.9	7.1
Pseudomonas sp.	NFACC16-2	GCA_900119205.1	60.6	6.6
Pseudomonas sp.	HMSC060F12	GCA_001836625.1	66.3	6.6
Pseudomonas sp.	P7548	GCA_013386585.1	61.1	7.0
Pseudomonas sp.	HMSC08G10	GCA_001808425.1	62.5	5.2
Pseudomonas sp.	IPO3779	GCA_013386785.1	60.9	7.1
Pseudomonas sp.	BMS12	GCA_001592875.1	64.0	4.5
Pseudomonas sp.	HMSC065H01	GCA_001815235.1	66.0	6.9
Pseudomonas sp.	NFACC23-1	GCA_900109275.1	60.6	6.6
Pseudomonas sp.	R5(2019)	GCA_009905435.1	60.3	6.0
Pseudomonas sp.	SBT1-2	GCA_028640875.1	62.9	7.0
Pseudomonas sp.	NFACC17-2	GCA_900104075.1	60.6	6.6
Pseudomonas sp.	YL2	GCA_023063025.1	60.2	6.2
Pseudomonas sp.	Irchel 3E19	GCA_900187585.1	59.1	6.5
Pseudomonas sp.	Xaverov 83	GCA_019352905.1	60.7	6.8
Pseudomonas sp.	TNT3	GCA_010095445.2	58.6	6.5
Pseudomonas sp.	HMSC066A08	GCA_001813025.1	66.0	6.8
Pseudomonas sp.	HMSC065H02	GCA_001813325.1	66.0	7.0
Pseudomonas sp.	HMSC067G02	GCA_001813485.1	66.3	6.6
Pseudomonas sp.	HMSC063H08	GCA_001813645.1	65.9	7.1
Pseudomonas sp.	MB-090624	GCA_003205245.1	62.0	6.1
Pseudomonas sp.	Xaverov 259	GCA_019352915.1	59.9	6.8
Pseudomonas sp.	S25	GCA_022790535.1	57.1	5.9
Pseudomonas sp.	NCPPB 3149	GCA_013386605.1	60.5	6.9
Pseudomonas sp.	297	GCA_003935465.1	62.0	5.2
Pseudomonas sp.	C2B4	GCA_013359695.1	59.7	6.8
Pseudomonas sp.	Sample_11	GCA_004307505.1	60.1	5.9
Pseudomonas sp.	L13	GCA_009935965.1	60.0	6.4

Pseudomonas sp.	Sample_23	GCA_004307595.1	60.0	6.1
Pseudomonas sp.	PNPG3	GCA_023093815.1	62.3	6.6
Pseudomonas sp.	Sample_14	GCA_004307515.1	60.1	6.0
Pseudomonas sp.	Sample_20	GCA_004307585.1	60.0	5.9
Pseudomonas sp.	B2021	GCA_018135665.1	60.3	6.1
Pseudomonas sp.	AD21	GCA_002878485.1	60.1	6.4
Pseudomonas sp.	57B-090624	GCA_003234055.1	66.4	7.1
Pseudomonas sp.	JG-B	GCA_009662095.1	62.9	6.0
Pseudomonas sp.	R81	GCA_000257625.1	60.3	6.2
Pseudomonas sp.	Sample_21	GCA_004307525.1	60.0	6.1
Pseudomonas sp.	31 R 17	GCA_900004915.1	60.7	6.1
Pseudomonas sp.	361	GCA_003935475.1	62.4	5.4
Pseudomonas sp.	HMSC058C05	GCA_001809775.1	65.9	7.0
Pseudomonas sp.	S4_EA_1b	GCA_016307435.1	59.9	7.5
Pseudomonas sp.	SG-MS2	GCA_009933855.1	61.8	5.6
Pseudomonas sp.	GM24	GCA_000282235.1	59.1	6.5
Pseudomonas sp.	PICF6	GCA_009831705.1	60.4	5.9
Pseudomonas sp.	18.1.10	GCA_024655625.1	61.1	6.6
Pseudomonas sp.	Sample_24	GCA_004307605.1	59.0	6.2
Pseudomonas sp.	F1002	GCA_013385525.1	60.5	7.7
Pseudomonas sp.	Sample_9	GCA_004307535.1	60.0	6.1
Pseudomonas sp.	HMWF021	GCA_003061005.1	60.3	6.0
Pseudomonas sp.	CM27	GCA_013179515.1	62.7	5.9
Pseudomonas sp.	HMWF011	GCA_003094675.1	59.9	6.0
Pseudomonas sp.	CM25	GCA_013179705.1	62.5	6.2
Pseudomonas sp.	TNT11	GCA_023072625.1	60.4	5.9
Pseudomonas sp.	HMSC75E02	GCA_001838695.1	66.9	6.9
Pseudomonas sp.	R62	GCA_000257605.1	58.9	6.6
Pseudomonas sp.	HMWF006	GCA_003061205.1	59.9	6.3
Pseudomonas sp.	K5002	GCA_013386655.1	61.0	6.8
Pseudomonas sp.	N24CT	GCA_015234375.1	61.0	4.0
Pseudomonas sp.	Gw_Bodden_bin_241	GCA_017992845.1	60.1	6.0
Pseudomonas sp.	K-2018 MAG007	GCA_029945815.1	59.1	6.7
Pseudomonas sp.	BIN42	GCA_017744435.1	63.4	5.6
Pseudomonas sp.	NID84	GCA_014333415.1	58.9	6.4
Pseudomonas sp.	SupBloom_Metag_081	GCA_016462755.1	60.0	6.0
Pseudomonas sp.	L2_037_039G1_dasL2_037_039G1_maxbin2.maxbin.014sta_sub	GCA_018371705.1	58.8	4.8
Pseudomonas sp.	UBA11276	GCA_003535575.1	58.3	5.6
Pseudomonas sp.	ALOHA_A2.5_105	GCA_022448005.1	60.1	5.0
Pseudomonas sp.	UBA11304	GCA_003531165.1	58.7	4.7
Pseudomonas sp.	Gw_Prim_bin_4	GCA_018063765.1	69.5	1.4
Pseudomonas sp.	Go_UH_bin_221	GCA_018001355.1	62.2	1.3
Pseudomonas sp.	Gw_Prim_bin_24	GCA_018063935.1	60.3	1.9
Pseudomonas sp.	nzgw589	GCA_023265965.1	60.6	5.9
Pseudomonas sp.	Gw_UH_bin_155	GCA_018055685.1	62.8	3.1
Pseudomonas sp.	Desfum bin_8_GBBB05	GCA_016742115.1	63.3	3.9

Pseudomonas sp.	Go_LeineEff_bin_115	GCA_018006235.1	58.9	1.8
Pseudomonas sp.	Go_SlPrim_bin_81	GCA_018006995.1	63.4	2.6
Pseudomonas sp.	Gw_Bodden_bin_212	GCA_018006215.1	63.3	1.6
Pseudomonas sp.	Gw_Inlet_bin_138	GCA_018006475.1	58.2	1.7
Pseudomonas sp.	Go_SlPrim_bin_198	GCA_018006795.1	61.8	2.1
Pseudomonas sp.	N17CT	GCA_015234435.1	63.0	4.8
Pseudomonas sp.	MAG_21	GCA_018780705.1	62.4	3.4
Pseudomonas sp.	9.1(2019)	GCA_009910445.1	58.7	5.0
Pseudomonas sp.	JI-2	GCA_022808215.1	64.0	4.5
Pseudomonas sp.	81_B	GCA_017491745.1	60.3	3.5
Pseudomonas sp.	UBA6549	GCA_002434525.1	55.4	4.6
Pseudomonas sp.	2(2015)	GCA_000955865.1	62.9	5.9
Pseudomonas sp.	UBA7233	GCA_002473025.1	55.3	5.0
Pseudomonas sp.	UBA3149	GCA_002364655.1	55.4	5.2
Pseudomonas sp.	UBA4102	GCA_002383245.1	55.6	5.1
Pseudomonas sp.	UBA7500	GCA_002478435.1	64.3	5.5
Pseudomonas sp.	PGPPP2	GCA_002255825.1	59.7	6.1
Pseudomonas sp.	UBA7721	GCA_002482145.1	61.9	4.7
Pseudomonas sp.	UBA5666	GCA_002420785.1	61.3	4.9
Pseudomonas sp.	UBA7456	GCA_002479855.1	61.8	4.6
Pseudomonas sp.	UBA7336	GCA_002494295.1	62.2	5.5
Pseudomonas sp.	16_A	GCA_017491665.1	64.2	5.9
Pseudomonas sp.	UBA6753	GCA_002453565.1	63.4	5.4
Pseudomonas sp.	32_A	GCA_017490905.1	62.8	5.8
Pseudomonas sp.	UBA5568	GCA_002425525.1	61.8	4.6
Pseudomonas sp.	UBA3153	GCA_002364635.1	65.5	4.3
Pseudomonas sp.	UBA6718	GCA_002454255.1	65.6	4.0
Pseudomonas sp.	UBA4034	GCA_002383725.1	61.0	5.4
Pseudomonas sp.	UBA6310	GCA_002438225.1	65.6	4.6
Pseudomonas sp.	22 E 5	GCA_900004705.1	60.2	14.1
Pseudomonas sp.	UBA6699	GCA_002454615.1	62.6	5.1
Pseudomonas sp.	UBA2522	GCA_002342925.1	61.8	4.2
Pseudomonas sp.	UBA2311	GCA_002345345.1	62.9	4.7
Pseudomonas sp.	HMWF010	GCA_003061425.1	61.4	5.9
Pseudomonas sp.	UBA6315	GCA_002438125.1	64.6	5.4
Pseudomonas sp.	UBA6276	GCA_002438365.1	62.4	4.5
Pseudomonas sp.	UBA6554	GCA_002434405.1	62.8	5.4
Pseudomonas sp.	UBA2628	GCA_002360105.1	62.7	4.6
Pseudomonas sp.	UBA800	GCA_002296625.1	61.9	5.6
Pseudomonas sp.	UBA2047	GCA_002333285.1	62.7	4.6
Pseudomonas sp.	CrR14	GCA_013179595.1	60.7	3.1
Pseudomonas sp.	CrR25	GCA_013179485.1	63.9	5.4
Pseudomonas sp.	OA3	GCA_016463995.1	62.6	3.5

Table S4

Information on Genome sequences of 299 uncharacterized *Pseudomonas* spp. whose taxonomic affiliations were predicted by the AppIndels web server

Species name	Strain	Assembly level	Accession no	GC Content (%)	Genome Length (mb)	Predicted affiliation	No. of shared CSIs
**Assembly level: Chr**-Chromosome, **Comp**-Complete, **Con**- Contigs, **Scaff**-Scaffold							
**Strains predicted as *P. aeruginosa* (64 strains)**							
Pseudomonas sp.	2VD	Con	GCA_005503585.1	66.3	6.4	*P. aeruginosa*	7
Pseudomonas sp.	3PA37B6	Con	GCA_005503545.1	65.7	7.1	*P. aeruginosa*	7
Pseudomonas sp.	17023526	Con	GCA_024718355.1	66.2	6.8	*P. aeruginosa*	7
Pseudomonas sp.	17023671	Con	GCA_024714085.1	66.2	6.8	*P. aeruginosa*	7
Pseudomonas sp.	17033095	Con	GCA_024714025.1	66.1	6.7	*P. aeruginosa*	7
Pseudomonas sp.	17053182	Con	GCA_024714065.1	66.1	6.8	*P. aeruginosa*	7
Pseudomonas sp.	17053418	Con	GCA_024718335.1	65.9	7.1	*P. aeruginosa*	7
Pseudomonas sp.	17053703	Con	GCA_024714105.1	66.5	6.3	*P. aeruginosa*	7
Pseudomonas sp.	17063399	Con	GCA_024718315.1	66.1	6.9	*P. aeruginosa*	7
Pseudomonas sp.	17072548	Con	GCA_024714045.1	66.1	6.9	*P. aeruginosa*	7
Pseudomonas sp.	17073326	Con	GCA_024714155.1	66.2	6.4	*P. aeruginosa*	7
Pseudomonas sp.	17102422	Con	GCA_024714005.1	66.5	6.3	*P. aeruginosa*	7
Pseudomonas sp.	17103552	Con	GCA_024718395.1	65.9	7.0	*P. aeruginosa*	7
Pseudomonas sp.	17104299	Con	GCA_024714125.1	66.4	6.4	*P. aeruginosa*	7
Pseudomonas sp.	18073667	Con	GCA_024714305.1	66.1	7.0	*P. aeruginosa*	7
Pseudomonas sp.	18082547	Con	GCA_024714345.1	66.0	6.9	*P. aeruginosa*	7
Pseudomonas sp.	18082551	Con	GCA_024718465.1	66.0	6.8	*P. aeruginosa*	7
Pseudomonas sp.	18082574	Con	GCA_024718505.1	65.6	7.0	*P. aeruginosa*	7
Pseudomonas sp.	18083194	Con	GCA_024714255.1	66.3	6.6	*P. aeruginosa*	7
Pseudomonas sp.	18083202	Con	GCA_024714225.1	66.0	7.0	*P. aeruginosa*	7
Pseudomonas sp.	18083259	Con	GCA_024714235.1	66.2	6.6	*P. aeruginosa*	7
Pseudomonas sp.	18083286	Con	GCA_024714245.1	66.4	6.4	*P. aeruginosa*	7
Pseudomonas sp.	18084127	Con	GCA_024714165.1	66.0	7.1	*P. aeruginosa*	5
Pseudomonas sp.	18092229	Con	GCA_024718485.1	66.0	6.8	*P. aeruginosa*	7
Pseudomonas sp.	18093371	Con	GCA_024714315.1	65.9	6.3	*P. aeruginosa*	6
Pseudomonas sp.	18101001-2	Con	GCA_024714145.1	65.6	7.0	*P. aeruginosa*	7
Pseudomonas sp.	18102011	Con	GCA_024718435.1	65.6	7.0	*P. aeruginosa*	7
Pseudomonas sp.	18103014	Con	GCA_024714205.1	65.9	6.9	*P. aeruginosa*	7
Pseudomonas sp.	18113298	Con	GCA_024714365.1	66.5	6.3	*P. aeruginosa*	7
Pseudomonas sp.	19062259	Con	GCA_024714385.1	66.1	6.3	*P. aeruginosa*	6
Pseudomonas sp.	19064969	Con	GCA_024714425.1	66.1	6.7	*P. aeruginosa*	7
Pseudomonas sp.	19072337-2	Con	GCA_024714405.1	66.1	6.8	*P. aeruginosa*	7
Pseudomonas sp.	19082381	Con	GCA_024714445.1	66.1	6.8	*P. aeruginosa*	7
Pseudomonas sp.	AF1	Con	GCA_016820215.1	66.0	5.9	*P. aeruginosa*	7
Pseudomonas sp.	AFW1	Con	GCA_016820235.1	66.0	7.3	*P. aeruginosa*	7
Pseudomonas sp.	AK6U	Comp	GCA_002843285.1	65.8	6.9	*P. aeruginosa*	7
Pseudomonas sp.	B111	Comp	GCA_026428275.1	66.1	6.9	*P. aeruginosa*	7
Pseudomonas sp.	BDPW	Con	GCA_016820275.1	66.3	4.4	*P. aeruginosa*	7
Pseudomonas sp.	BIS	Con	GCA_016820195.1	66.0	6.7	*P. aeruginosa*	6
Pseudomonas sp.	BIS1	Con	GCA_016820175.1	66.1	6.3	*P. aeruginosa*	6
Pseudomonas sp.	FDAARGOS_761	Comp	GCA_013343155.1	66.4	6.4	*P. aeruginosa*	7
Pseudomonas sp.	PAH14	Con	GCA_018551555.1	65.1	7.3	*P. aeruginosa*	7
Pseudomonas sp.	Pseudomonas _assembly	Con	GCA_950101725.1	66.5	6.3	*P. aeruginosa*	7
Pseudomonas sp.	PS1(2021)	Comp	GCA_020405165.1	65.5	7.8	*P. aeruginosa*	7
Pseudomonas sp.	RGIG3665	Con	GCA_017455265.1	66.5	5.9	*P. aeruginosa*	6
Pseudomonas sp.	S33	Con	GCA_016624455.1	66.4	6.5	*P. aeruginosa*	7

Pseudomonas sp.	S68	Con	GCA_016658555.1	66.4	6.8	*P. aeruginosa*	7
Pseudomonas sp.	203-8	Scaff	GCA_020708595.1	66.4	6.5	*P. aeruginosa*	7
Pseudomonas sp.	18081308	Scaff	GCA_024718375.1	66.4	6.4	*P. aeruginosa*	7
Pseudomonas sp.	P20	Scaff	GCA_016463925.1	65.9	6.8	*P. aeruginosa*	7
Pseudomonas sp.	CP-1	Scaff	GCA_021213715.1	65.6	7.0	*P. aeruginosa*	7
Pseudomonas sp.	P22	Scaff	GCA_016464635.1	65.9	6.8	*P. aeruginosa*	7
Pseudomonas sp.	P179	Scaff	GCA_000478485.2	65.6	6.9	*P. aeruginosa*	7
Pseudomonas sp.	HMSC057H01	Scaff	GCA_001836405.1	66.1	6.7	*P. aeruginosa*	7
Pseudomonas sp.	HMSC072F09	Scaff	GCA_001809805.1	66.0	6.8	*P. aeruginosa*	6
Pseudomonas sp.	HMSC16B01	Scaff	GCA_001808305.1	66.1	6.9	*P. aeruginosa*	7
Pseudomonas sp.	HMSC076A11	Scaff	GCA_001810205.1	66.3	6.0	*P. aeruginosa*	7
Pseudomonas sp.	HMSC060F12	Scaff	GCA_001836625.1	66.3	6.6	*P. aeruginosa*	7
Pseudomonas sp.	HMSC065H01	Scaff	GCA_001815235.1	66.0	6.9	*P. aeruginosa*	6
Pseudomonas sp.	HMSC066A08	Scaff	GCA_001813025.1	66.0	6.8	*P. aeruginosa*	5
Pseudomonas sp.	HMSC065H02	Scaff	GCA_001813325.1	66.0	7.0	*P. aeruginosa*	6
Pseudomonas sp.	HMSC067G02	Scaff	GCA_001813485.1	66.3	6.6	*P. aeruginosa*	7
Pseudomonas sp.	HMSC063H08	Scaff	GCA_001813645.1	65.9	7.1	*P. aeruginosa*	7
Pseudomonas sp.	HMSC058C05	Scaff	GCA_001809775.1	65.9	7.0	*P. aeruginosa*	7
**Strains predicted as *Pseudomonas sensu stricto* (46 strains)**							
Pseudomonas sp.	273	Comp	GCA_029201425.1	67.5	7.5	*Pseudomonas sensu stricto*	6
Pseudomonas sp.	ADPe	Comp	GCA_014843515.1	66.9	7.2	*Pseudomonas sensu stricto*	6
Pseudomonas sp.	ATCC 13867	Comp	GCA_000349845.1	65.2	5.7	*Pseudomonas sensu stricto*	6
Pseudomonas sp.	AU11447	Con	GCA_001672555.2	64.6	6.1	*Pseudomonas sensu stricto*	6
Pseudomonas sp.	AU12215	Con	GCA_001675465.2	64.1	6.2	*Pseudomonas sensu stricto*	6
Pseudomonas sp.	BJa5	Con	GCA_030272875.1	64.9	6.9	*Pseudomonas sensu stricto*	6
Pseudomonas sp.	GCEP-101	Comp	GCA_025133575.1	66.3	6.2	*Pseudomonas sensu stricto*	6
Pseudomonas sp.	GD03691	Con	GCA_029844365.1	64.8	6.3	*Pseudomonas sensu stricto*	6
Pseudomonas sp.	GD03903	Con	GCA_029840045.1	64.8	6.9	*Pseudomonas sensu stricto*	6
Pseudomonas sp.	GD04087	Con	GCA_029836255.1	64.8	6.9	*Pseudomonas sensu stricto*	6
Pseudomonas sp.	LA21	Con	GCA_022819105.1	65.3	6.0	*Pseudomonas sensu stricto*	5
Pseudomonas sp.	M1	Comp	GCA_022760135.1	67.3	7.0	*Pseudomonas sensu stricto*	6
Pseudomonas sp.	NBRC 111135	Con	GCA_001320705.1	64.5	3.8	*Pseudomonas sensu stricto*	6
Pseudomonas sp.	PDM17	Con	GCA_014851985.1	65.2	6.6	*Pseudomonas sensu stricto*	6
Pseudomonas sp.	PDM18	Con	GCA_014852585.1	64.9	6.4	*Pseudomonas sensu stricto*	6
Pseudomonas sp.	PDM19	Con	GCA_014852215.1	65.3	6.8	*Pseudomonas sensu stricto*	6
Pseudomonas sp.	PDM20	Con	GCA_014852575.1	65.4	6.7	*Pseudomonas sensu stricto*	6
Pseudomonas sp.	PDM21	Con	GCA_014852505.1	65.3	6.7	*Pseudomonas sensu stricto*	6
Pseudomonas sp.	PDM22	Con	GCA_014851965.1	65.4	6.7	*Pseudomonas sensu stricto*	6

Pseudomonas sp.	PDM23	Con	GCA_014852225.1	65.3	6.7	*Pseudomonas sensu stricto*	6
Pseudomonas sp.	PDM33	Con	GCA_019219265.1	65.5	6.4	*Pseudomonas sensu stricto*	6
Pseudomonas sp.	PDNC002	Comp	GCA_016919445.1	64.9	6.4	*Pseudomonas sensu stricto*	6
Pseudomonas sp.	PI1	Con	GCA_000812405.1	66.7	6.1	*Pseudomonas sensu stricto*	6
Pseudomonas sp.	PSE14	Comp	GCA_029203285.1	65.2	6.0	*Pseudomonas sensu stricto*	6
Pseudomonas sp.	R3.Fl	Con	GCA_023516765.1	67.2	7.5	*Pseudomonas sensu stricto*	6
Pseudomonas sp.	SCB32	Comp	GCA_009189165.1	64.6	6.3	*Pseudomonas sensu stricto*	5
Pseudomonas sp.	UMA601	Con	GCA_013337485.1	67.4	4.8	*Pseudomonas sensu stricto*	6
Pseudomonas sp.	UMA603	Con	GCA_013337585.1	67.4	2.5	*Pseudomonas sensu stricto*	6
Pseudomonas sp.	UMA643	Con	GCA_013337595.1	67.4	3.7	*Pseudomonas sensu stricto*	6
Pseudomonas sp.	UMC3103	Con	GCA_013337545.1	67.4	4.5	*Pseudomonas sensu stricto*	6
Pseudomonas sp.	UMC3106	Con	GCA_013337565.1	67.5	3.2	*Pseudomonas sensu stricto*	6
Pseudomonas sp.	UMC3129	Con	GCA_013337495.1	67.4	4.8	*Pseudomonas sensu stricto*	6
Pseudomonas sp.	UMC631	Con	GCA_013337505.1	67.4	3.0	*Pseudomonas sensu stricto*	6
Pseudomonas sp.	UMC76	Con	GCA_014170435.1	67.3	2.9	*Pseudomonas sensu stricto*	6
Pseudomonas sp.	UME83	Con	GCA_014170285.1	67.3	7.0	*Pseudomonas sensu stricto*	6
Pseudomonas sp.	ZM23	Con	GCA_026686735.1	64.0	6.2	*Pseudomonas sensu stricto*	5
Pseudomonas sp.	ZM24	Con	GCA_024259765.1	63.6	6.5	*Pseudomonas sensu stricto*	5
Pseudomonas sp.	ZM25	Con	GCA_024259725.1	63.5	6.5	*Pseudomonas sensu stricto*	5
Pseudomonas sp.	HS-18	Comp	GCA_020401845.1	64.6	6.6	*Pseudomonas sensu stricto*	6
Pseudomonas sp.	AAC	Scaff	GCA_000725445.1	67.0	7.1	*Pseudomonas sensu stricto*	6
Pseudomonas sp.	NBRC 100443	Scaff	GCA_030268185.1	67.0	7.2	*Pseudomonas sensu stricto*	6
Pseudomonas sp.	EGD-AKN5	Scaff	GCA_001669755.1	67.0	7.1	*Pseudomonas sensu stricto*	6
Pseudomonas sp.	RW407	Scaff	GCA_003176655.1	67.4	7.3	*Pseudomonas sensu stricto*	6
Pseudomonas sp.	30_B	Scaff	GCA_017491605.1	65.0	6.2	*Pseudomonas sensu stricto*	5
Pseudomonas sp.	21	Scaff	GCA_000955805.1	65.6	6.3	*Pseudomonas sensu stricto*	6
Pseudomonas sp.	HMSC75E02	Scaff	GCA_001838695.1	66.9	6.9	*Pseudomonas sensu stricto*	6
**Strains predicted as *Aquipseudomonas* (21 strains)**							
Pseudomonas sp.	GD03869	Con	GCA_029840705.1	66.4	6.3	*Aquipseudomonas*	6
Pseudomonas sp.	J452	Chr	GCA_024666525.1	63.2	4.9	*Aquipseudomonas*	6

Pseudomonas sp.	BLCC-B13	Con	GCA_028550635.1	65.4	4.5	*Aquipseudomonas*	6
Pseudomonas sp.	F(2018)	Con	GCA_005508865.1	65.6	4.7	*Aquipseudomonas*	6
Pseudomonas sp.	GD03875	Con	GCA_029840585.1	66.3	6.4	*Aquipseudomonas*	6
Pseudomonas sp.	GD03985	Con	GCA_029838465.1	66.3	6.3	*Aquipseudomonas*	6
Pseudomonas sp.	GD04015	Con	GCA_029837675.1	66.4	6.4	*Aquipseudomonas*	6
Pseudomonas sp.	GD04019	Con	GCA_029837695.1	65.0	4.8	*Aquipseudomonas*	6
Pseudomonas sp.	GD04042	Con	GCA_029837175.1	66.4	5.4	*Aquipseudomonas*	6
Pseudomonas sp.	GD04045	Con	GCA_029837115.1	65.0	6.2	*Aquipseudomonas*	6
Pseudomonas sp.	GOM6	Con	GCA_029537485.1	62.8	4.3	*Aquipseudomonas*	5
Pseudomonas sp.	PDM15	Con	GCA_014851565.1	64.8	4.8	*Aquipseudomonas*	6
Pseudomonas sp.	PDM16	Con	GCA_014851865.1	63.2	5.1	*Aquipseudomonas*	6
Pseudomonas sp.	WS 5013	Con	GCA_012985845.1	65.5	4.5	*Aquipseudomonas*	6
Pseudomonas sp.	SO81	Comp	GCA_030374125.1	65.4	4.8	*Aquipseudomonas*	6
Pseudomonas sp.	8AS	Scaff	GCA_902506505.1	65.8	4.3	*Aquipseudomonas*	4
Pseudomonas sp.	L-22-4S-12	Scaff	GCA_009799935.1	65.8	4.5	*Aquipseudomonas*	4
Pseudomonas sp.	R-28-1W-6	Scaff	GCA_009799925.1	65.8	4.3	*Aquipseudomonas*	4
Pseudomonas sp.	ML96	Scaff	GCA_000761545.1	64.8	4.8	*Aquipseudomonas*	6
Pseudomonas sp.	BMS12	Scaff	GCA_001592875.1	64.0	4.5	*Aquipseudomonas*	6
Pseudomonas sp.	UBA6718	Scaff	GCA_002454255.1	65.6	4.0	*Aquipseudomonas*	6
**Strain predicted as *Caenipseudomonas* (1 strain)**							
Pseudomonas sp.	Go_SlPrim_bin_81	Scaff	GCA_018006995.1	63.4	2.6	*Caenipseudomonas*	7
**Strains predicted as *Chryseomonas* (32 strains)**							
Pseudomonas sp.	313	Con	GCA_000316965.1	65.3	5.2	*Chryseomonas*	11
Pseudomonas sp.	AS2.8	Con	GCA_014190935.1	66.3	4.9	*Chryseomonas*	11
Pseudomonas sp.	BAV 2493	Con	GCA_009765535.1	65.4	2.6	*Chryseomonas*	11
Pseudomonas sp.	BAV 4579	Con	GCA_009765395.1	65.4	5.5	*Chryseomonas*	11
Pseudomonas sp.	GM_Psu_1	Con	GCA_026544185.1	66.0	5.0	*Chryseomonas*	11
Pseudomonas sp.	GM_Psu_2	Con	GCA_026541025.1	66.0	5.3	*Chryseomonas*	11
Pseudomonas sp.	HUK17	Con	GCA_001566765.1	65.5	2.6	*Chryseomonas*	6
Pseudomonas sp.	LTJR-52	Comp	GCA_003691465.1	55.2	5.5	*Chryseomonas*	11
Pseudomonas sp.	MAG002Y	Con	GCA_019400845.1	55.4	5.5	*Chryseomonas*	11
Pseudomonas sp.	PS02302	Con	GCA_029959585.1	65.5	5.4	*Chryseomonas*	11
Pseudomonas sp.	RIT 411	Con	GCA_003057735.2	66.2	5.3	*Chryseomonas*	11
Pseudomonas sp.	S1C77_SP397	Con	GCA_913775925.1	66.4	6.7	*Chryseomonas*	11
Pseudomonas sp.	S2C3242_SP226	Con	GCA_913776055.1	66.3	6.5	*Chryseomonas*	11
Pseudomonas sp.	SP131_2_metabat2_genome_mining.13	Con	GCA_913778735.1	66.1	7.0	*Chryseomonas*	11
Pseudomonas sp.	SP152_1_metabat2_genome_mining.5	Con	GCA_913776115.1	66.7	6.9	*Chryseomonas*	11
Pseudomonas sp.	SP29_3_metabat2_genome_mining.23	Con	GCA_913777085.1	66.5	7.2	*Chryseomonas*	10
Pseudomonas sp.	SP3_3_metabat2_genome_mining.12	Con	GCA_913774555.1	66.8	6.0	*Chryseomonas*	11
Pseudomonas sp.	SP403_2_metabat2_genome_mining.12	Con	GCA_913774965.1	66.7	6.0	*Chryseomonas*	11
Pseudomonas sp.	SP421_3_metabat2_genome_mining.10	Con	GCA_913778655.1	66.5	6.1	*Chryseomonas*	11
Pseudomonas sp.	WAC2	Con	GCA_030403525.1	55.4	5.0	*Chryseomonas*	11
Pseudomonas sp.	HPB0071	Scaff	GCA_000478505.2	55.1	5.7	*Chryseomonas*	11
Pseudomonas sp.	Snoq117.2	Scaff	GCA_900110545.1	64.8	5.5	*Chryseomonas*	11
Pseudomonas sp.	MS15	Scaff	GCA_013277805.1	65.1	5.2	*Chryseomonas*	11
Pseudomonas sp.	JUb52	Scaff	GCA_004341115.1	65.5	5.1	*Chryseomonas*	11
Pseudomonas sp.	EpSL25	Scaff	GCA_001512295.1	65.5	5.4	*Chryseomonas*	11
Pseudomonas sp.	PLB05	Scaff	GCA_021106615.1	65.2	5.1	*Chryseomonas*	11
Pseudomonas sp.	HR1	Scaff	GCA_030175875.1	66.1	5.1	*Chryseomonas*	11
Pseudomonas sp.	CBMAI 2609	Scaff	GCA_029872515.1	65.5	5.3	*Chryseomonas*	11

Pseudomonas sp.	UBA6549	Scaff	GCA_002434525.1	55.4	4.6	*Chryseomonas*	10
Pseudomonas sp.	UBA7233	Scaff	GCA_002473025.1	55.3	5.0	*Chryseomonas*	10
Pseudomonas sp.	UBA3149	Scaff	GCA_002364655.1	55.4	5.2	*Chryseomonas*	11
Pseudomonas sp.	UBA4102	Scaff	GCA_002383245.1	55.6	5.1	*Chryseomonas*	11
**Strains predicted as *Ectopseudomonas* (46 strains)**							
Pseudomonas sp.	07-Jan	Con	GCA_000742775.1	62.0	4.9	*Ectopseudomonas*	5
Pseudomonas sp.	905_Psudmo1	Comp	GCA_029691665.1	62.5	5.5	*Ectopseudomonas*	5
Pseudomonas sp.	AA-38	Comp	GCA_029457475.1	63.3	5.5	*Ectopseudomonas*	4
Pseudomonas sp.	ALS1131	Con	GCA_007049925.1	62.4	5.6	*Ectopseudomonas*	4
Pseudomonas sp.	ALS1279	Con	GCA_007049955.1	62.5	5.3	*Ectopseudomonas*	4
Pseudomonas sp.	AOB-7	Con	GCA_003696305.1	66.6	4.9	*Ectopseudomonas*	3
Pseudomonas sp.	B11D7D	Comp	GCA_013410155.2	62.5	5.4	*Ectopseudomonas*	4
Pseudomonas sp.	BMW13	Con	GCA_013357275.1	65.0	5.2	*Ectopseudomonas*	5
Pseudomonas sp.	DS1.001	Con	GCA_013823455.1	65.0	5.8	*Ectopseudomonas*	5
Pseudomonas sp.	EGD-AK9	Con	GCA_000465935.2	65.6	5.8	*Ectopseudomonas*	3
Pseudomonas sp.	EggHat1	Con	GCA_014892995.1	62.5	5.3	*Ectopseudomonas*	5
Pseudomonas sp.	GD03721	Comp	GCA_029814955.1	62.0	4.7	*Ectopseudomonas*	5
Pseudomonas sp.	GD03722	Con	GCA_029843605.1	62.3	6.4	*Ectopseudomonas*	5
Pseudomonas sp.	GD03919	Comp	GCA_029814935.1	62.0	4.7	*Ectopseudomonas*	5
Pseudomonas sp.	GD04158	Con	GCA_029834875.1	64.6	5.0	*Ectopseudomonas*	5
Pseudomonas sp.	GOM7	Comp	GCA_026723825.1	63.4	5.4	*Ectopseudomonas*	4
Pseudomonas sp.	GV_Bin_12	Con	GCA_009360675.1	63.6	5.8	*Ectopseudomonas*	5
Pseudomonas sp.	KB-10	Con	GCA_018221585.1	63.0	5.2	*Ectopseudomonas*	5
Pseudomonas sp.	KHPS1	Comp	GCA_024205205.1	64.4	5.1	*Ectopseudomonas*	5
Pseudomonas sp.	LPH1	Comp	GCA_002037565.1	62.7	5.2	*Ectopseudomonas*	5
Pseudomonas sp.	Marseille-Q0931	Con	GCA_940789645.1	62.8	5.2	*Ectopseudomonas*	5
Pseudomonas sp.	MDMC17	Con	GCA_003269375.2	62.7	6.8	*Ectopseudomonas*	4
Pseudomonas sp.	MDMC216	Con	GCA_003269355.2	62.7	5.5	*Ectopseudomonas*	5
Pseudomonas sp.	MDMC224	Con	GCA_003269315.1	62.5	5.6	*Ectopseudomonas*	5
Pseudomonas sp.	MSPm1	Chr	GCA_014109765.1	62.4	5.7	*Ectopseudomonas*	5
Pseudomonas sp.	NFACC19-2	Con	GCA_900119125.1	62.8	5.1	*Ectopseudomonas*	5
Pseudomonas sp.	P818	Con	GCA_000418555.1	63.4	5.1	*Ectopseudomonas*	5
Pseudomonas sp.	phDV1	Chr	GCA_003408635.1	62.3	4.7	*Ectopseudomonas*	5
Pseudomonas sp.	REST10	Chr	GCA_029542605.1	65.0	5.1	*Ectopseudomonas*	5
Pseudomonas sp.	RGIG627	Con	GCA_017418485.1	62.6	6.4	*Ectopseudomonas*	3
Pseudomonas sp.	THAF187a	Comp	GCA_009363395.1	64.8	5.3	*Ectopseudomonas*	5
Pseudomonas sp.	THAF42	Comp	GCA_009363475.1	64.8	5.3	*Ectopseudomonas*	5
Pseudomonas sp.	WS 5019	Con	GCA_012985785.1	62.7	5.2	*Ectopseudomonas*	5
Pseudomonas sp.	YY-1	Con	GCA_002843095.1	62.2	5.0	*Ectopseudomonas*	4
Pseudomonas sp.	Z8(2022)	Comp	GCA_025837155.1	62.9	4.5	*Ectopseudomonas*	4
Pseudomonas sp.	ZH-FAD	Con	GCA_002803095.1	62.2	5.0	*Ectopseudomonas*	5
Pseudomonas sp.	8O	Scaff	GCA_902506495.1	62.5	5.2	*Ectopseudomonas*	5
Pseudomonas sp.	NFPP33	Scaff	GCA_900103725.1	62.6	5.3	*Ectopseudomonas*	5
Pseudomonas sp.	8Z	Scaff	GCA_902506535.1	60.9	4.8	*Ectopseudomonas*	4
Pseudomonas sp.	o96	Scaff	GCA_003935455.1	62.4	5.0	*Ectopseudomonas*	4
Pseudomonas sp.	Leaf83	Scaff	GCA_001422075.1	62.4	5.5	*Ectopseudomonas*	5
Pseudomonas sp.	NCCP-436	Scaff	GCA_019656335.1	61.4	3.7	*Ectopseudomonas*	3
Pseudomonas sp.	HS-2	Scaff	GCA_004369025.1	62.1	6.4	*Ectopseudomonas*	5
Pseudomonas sp.	297	Scaff	GCA_003935465.1	62.0	5.2	*Ectopseudomonas*	5
Pseudomonas sp.	Gw_UH_bin_155	Scaff	GCA_018055685.1	62.8	3.1	*Ectopseudomonas*	4
Pseudomonas sp.	OA3	Scaff	GCA_016463995.1	62.6	3.5	*Ectopseudomonas*	3
**Strains predicted as *Geopseudomonas* (4 strains)**							
Pseudomonas sp.	A-1	Con	GCA_004801855.1	68.2	4.4	*Geopseudomonas*	15
Pseudomonas sp.	OF001	Con	GCA_904426495.1	68.0	6.2	*Geopseudomonas*	14

Pseudomonas sp.	R2F_R2FSRR _metabat.60	Con	GCA_023700865.1	67.1	6.3	*Geopseudomonas*	11
Pseudomonas sp.	Gw_Prim_bin_4	Scaff	GCA_018063765.1	69.5	1.4	*Geopseudomonas*	4
**Strains predicted as *Halopseudomonas* (9 strains)**							
Pseudomonas sp.	5Ae-yellow	Con	GCA_014077575.1	56.2	4.2	*Halopseudomonas*	23
Pseudomonas sp.	FME51	Con	GCA_014897305.1	54.2	4.8	*Halopseudomonas*	23
Pseudomonas sp.	gcc21	Comp	GCA_012844345.1	58.3	4.0	*Halopseudomonas*	23
Pseudomonas sp.	NORP239	Con	GCA_016764935.1	57.5	5.9	*Halopseudomonas*	22
Pseudomonas sp.	NORP330	Con	GCA_016763285.1	61.0	5.7	*Halopseudomonas*	20
Pseudomonas sp.	OIL-1	Comp	GCA_010669185.1	58.3	4.2	*Halopseudomonas*	24
Pseudomonas sp.	SSM44	Con	GCA_003797905.1	56.2	5.8	*Halopseudomonas*	24
Pseudomonas sp.	WN033	Con	GCA_002287035.1	59.9	4.2	*Halopseudomonas*	21
Pseudomonas sp.	MYb185	Scaff	GCA_002979975.1	61.5	4.1	*Halopseudomonas*	24
**Strains predicted as *Metapseudomonas* (22 strains)**							
Pseudomonas sp.	1D4	Con	GCA_001728925.1	66.3	5.8	*Metapseudomonas*	3
Pseudomonas sp.	A46	Con	GCA_002196875.1	64.7	6.4	*Metapseudomonas*	5
Pseudomonas sp.	D(2018)	Con	GCA_005502555.1	63.5	6.3	*Metapseudomonas*	5
Pseudomonas sp.	DY-1	Comp	GCA_003626975.1	62.9	5.9	*Metapseudomonas*	5
Pseudomonas sp.	ENNP23	Con	GCA_001728945.1	66.3	6.1	*Metapseudomonas*	3
Pseudomonas sp.	JM0905a	Con	GCA_014700075.1	64.0	5.8	*Metapseudomonas*	5
Pseudomonas sp.	Pc102	Comp	GCA_018408275.1	66.8	6.7	*Metapseudomonas*	3
Pseudomonas sp.	PDM13	Con	GCA_025685765.1	66.3	6.4	*Metapseudomonas*	3
Pseudomonas sp.	Q1-7	Comp	GCA_028010285.1	64.8	5.8	*Metapseudomonas*	5
Pseudomonas sp.	SLBN-26	Con	GCA_006715895.1	67.2	6.3	*Metapseudomonas*	3
Pseudomonas sp.	TCU-HL1	Comp	GCA_001708505.1	63.2	6.3	*Metapseudomonas*	5
Pseudomonas sp.	LFM046	Scaff	GCA_000949385.2	64.3	6.0	*Metapseudomonas*	5
Pseudomonas sp.	BN414	Scaff	GCA_029871275.1	63.1	6.7	*Metapseudomonas*	5
Pseudomonas sp.	BN417	Scaff	GCA_029871205.1	64.6	6.6	*Metapseudomonas*	5
Pseudomonas sp.	BN606	Scaff	GCA_029873115.1	62.7	6.4	*Metapseudomonas*	5
Pseudomonas sp.	BN415	Scaff	GCA_029871195.1	63.2	6.1	*Metapseudomonas*	5
Pseudomonas sp.	BN411	Scaff	GCA_029871235.1	63.6	6.4	*Metapseudomonas*	5
Pseudomonas sp.	FeS53a	Scaff	GCA_000931385.1	67.3	5.9	*Metapseudomonas*	3
Pseudomonas sp.	BN102	Scaff	GCA_029871245.1	64.6	6.5	*Metapseudomonas*	5
Pseudomonas sp.	BN515	Scaff	GCA_029873155.1	63.9	6.3	*Metapseudomonas*	5
Pseudomonas sp.	57B-090624	Scaff	GCA_003234055.1	66.4	7.1	*Metapseudomonas*	3
Pseudomonas sp.	JG-B	Scaff	GCA_009662095.1	62.9	6.0	*Metapseudomonas*	5
**Strains predicted as *Phytopseudomonas* (17 strains)**							
Pseudomonas sp.	AG1028	Con	GCA_007993955.1	63.2	5.0	*Phytopseudomonas*	12
Pseudomonas sp.	Bi70	Con	GCA_918698015.1	63.0	7.3	*Phytopseudomonas*	12
Pseudomonas sp.	BIGb0408	Con	GCA_025961395.1	62.2	6.0	*Phytopseudomonas*	12
Pseudomonas sp.	CNPSo 3701	Con	GCA_028674975.1	63.1	5.0	*Phytopseudomonas*	12
Pseudomonas sp.	MEJ086	Con	GCA_030209025.1	62.4	6.9	*Phytopseudomonas*	12
Pseudomonas sp.	MM211	Comp	GCA_020386635.1	61.5	5.3	*Phytopseudomonas*	12
Pseudomonas sp.	PDM11	Con	GCA_014851505.1	63.3	4.7	*Phytopseudomonas*	12
Pseudomonas sp.	PDM12	Con	GCA_014852485.1	63.5	5.2	*Phytopseudomonas*	12
Pseudomonas sp.	S2C11432_SP223	Con	GCA_913775285.1	63.4	6.6	*Phytopseudomonas*	12
Pseudomonas sp.	S2C78296_SP133	Con	GCA_913778435.1	63.3	6.5	*Phytopseudomonas*	12
Pseudomonas sp.	sia0905	Con	GCA_019219245.1	63.6	4.9	*Phytopseudomonas*	11
Pseudomonas sp.	SP200_1_metabat2 _genome_mining.4 4	Con	GCA_913777955.1	63.3	4.9	*Phytopseudomonas*	11
Pseudomonas sp.	SP236_1_metabat2	Con	GCA_913775855.1	63.3	6.3	*Phytopseudomonas*	11

	_genome_mining.8						
Pseudomonas sp.	PA27	Scaff	GCA_001945445.1	63.1	5.4	*Phytopseudomonas*	12
Pseudomonas sp.	PA1	Scaff	GCA_001945375.1	63.1	5.3	*Phytopseudomonas*	10
Pseudomonas sp.	PA15	Scaff	GCA_001945395.1	63.2	5.5	*Phytopseudomonas*	12
Pseudomonas sp.	CrR14	Scaff	GCA_013179595.1	60.7	3.1	*Phytopseudomonas*	9
**Strains predicted as *Serpens* (2 strains)**							
Pseudomonas sp.	RL	Con	GCA_000647775.1	65.6	6.0	*Serpens*	3
Pseudomonas sp.	N24CT	Scaff	GCA_015234375.1	61.0	4.0	Serpens	3
**Strains predicted as *Stutzerimonas* (31 strains)**							
Pseudomonas sp.	TTU2014-105ASC	Con	GCA_001446975.1	61.7	4.0	*Stutzerimonas*	7
Pseudomonas sp.	WS 5018	Con	GCA_012985885.1	63.8	4.0	*Stutzerimonas*	7
Pseudomonas sp.	A192_concoct.bin.7	Con	GCA_019459105.1	63.7	5.8	*Stutzerimonas*	7
Pseudomonas sp.	ABC1	Comp	GCA_013395055.1	62.3	4.0	*Stutzerimonas*	6
Pseudomonas sp.	BAY1663	Con	GCA_000582595.1	65.0	4.9	*Stutzerimonas*	5
Pseudomonas sp.	BRH_c35	Con	GCA_000961835.1	61.1	6.2	*Stutzerimonas*	4
Pseudomonas sp.	C42_metabat.bin.8	Con	GCA_019459405.1	64.3	6.2	*Stutzerimonas*	4
Pseudomonas sp.	Choline-3u-10	Con	GCA_002836195.1	60.3	5.0	*Stutzerimonas*	7
Pseudomonas sp.	DF_1_3.23	Con	GCA_019105025.1	64.9	5.7	*Stutzerimonas*	7
Pseudomonas sp.	DNDY-54	Comp	GCA_019880365.1	60.2	4.4	*Stutzerimonas*	7
Pseudomonas sp.	IC_126	Con	GCA_004331835.1	60.8	4.6	*Stutzerimonas*	6
Pseudomonas sp.	M30B71	Con	GCA_018401165.1	62.6	6.2	*Stutzerimonas*	5
Pseudomonas sp.	MCMED-G45	Con	GCA_013911245.1	63.5	6.8	*Stutzerimonas*	5
Pseudomonas sp.	MT-1	Chr	GCA_000828755.1	60.2	4.9	*Stutzerimonas*	7
Pseudomonas sp.	MTM4	Comp	GCA_019355055.1	60.7	4.7	*Stutzerimonas*	6
Pseudomonas sp.	Q2-TVG4-2	Con	GCA_013620795.1	60.6	4.5	*Stutzerimonas*	7
Pseudomonas sp.	RS261_metabat.bin .8	Con	GCA_024709455.1	64.9	6.8	*Stutzerimonas*	6
Pseudomonas sp.	S5(2021)	Con	GCA_020075685.1	64.7	4.4	*Stutzerimonas*	7
Pseudomonas sp.	SCT	Con	GCA_003864275.1	62.5	4.8	*Stutzerimonas*	7
Pseudomonas sp.	SST3	Con	GCA_003325755.2	59.9	5.6	*Stutzerimonas*	7
Pseudomonas sp.	TTU2014-066ASC	Con	GCA_001446915.1	63.1	4.3	*Stutzerimonas*	7
Pseudomonas sp.	TTU2014-096BSC	Con	GCA_001446945.1	63.2	4.2	*Stutzerimonas*	7
Pseudomonas sp.	10B238	Scaff	GCA_000970615.1	60.2	4.9	*Stutzerimonas*	7
Pseudomonas sp.	MT4	Scaff	GCA_015956425.1	60.7	4.7	*Stutzerimonas*	6
Pseudomonas sp.	NP21570	Scaff	GCA_020523865.1	62.4	4.7	*Stutzerimonas*	7
Pseudomonas sp.	KSR10	Scaff	GCA_022355455.1	61.1	4.9	*Stutzerimonas*	7
Pseudomonas sp.	9Ag	Scaff	GCA_902506445.1	60.5	4.7	*Stutzerimonas*	7
Pseudomonas sp.	s199	Scaff	GCA_003935435.1	63.1	4.3	*Stutzerimonas*	7
Pseudomonas sp.	N17CT	Scaff	GCA_015234435.1	63.0	4.8	*Stutzerimonas*	6
Pseudomonas sp.	JI-2	Scaff	GCA_022808215.1	64.0	4.5	*Stutzerimonas*	7
Pseudomonas sp.	ALOHA_A2.5_105	Scaff	GCA_022448005.1	60.1	5.0	*Stutzerimonas*	7
**Strains predicted as *Thiopseudomons* (3 strains)**							
Pseudomonas sp.	AS08sgBPME_395	Con	GCA_012841125.1	48.8	7.2	*Thiopseudomonas*	4
Pseudomonas sp.	C27(2019)	Comp	GCA_008807395.1	48.6	3.2	*Thiopseudomonas*	5
Pseudomonas sp.	SO_2017_LW2 bin 68	Con	GCA_023228505.1	49.1	6.7	*Thiopseudomonas*	5
**Strains predicted as *Zestomons* (1 strain**							
Pseudomonas sp.	LS44	Comp	GCA_024730785.1	62.4	4.5	*Zestomonas*	3
